# Multimodal Mechanism of Antitumoral Ni(II) Thiosemicarbazones: Deep Mechanistic Understanding of ROS Dynamics, Albumin-Mediated Delivery, and DNA Targeting

**DOI:** 10.1021/acsomega.5c12067

**Published:** 2026-05-29

**Authors:** Lorenzo Verderi, Silvana Pinelli, Gloria Cenci, Chiara Maccari, Paola Mozzoni, Simone Fortunati, Marco Giannetto, Valentina Borghesani, Matteo Tegoni, Luca Ronda, Jesus Diaz, Mauro Carcelli, Giorgio Pelosi, Franco Bisceglie

**Affiliations:** 1 Department of Chemistry, Life Sciences and Environmental Sustainability, 9370 University of Parma, Parco Area delle Scienze 17/a, Parma 43124, Italy; 2 Department of Medicine and Surgery, 9370 University of Parma, Via Gramsci 14, Parma 43126, Italy; 3 Institute of Materials for Electronics and Magnetism, National Research Council (IMEM-CNR), Parco Area delle Scienze 37/a, Parma 43124, Italy; 4 Center of Excellence for Toxicological Research (CERT), 9370 University of Parma, Via Gramsci 14, Parma 43126, Italy; 5 Laboratory of Bioorganic Chemistry & Membrane Biophysics (L.O.B.O.). Departamento de Química Orgánica e Inorgánica, 16759 Universidad de Extremadura, Cáceres 10003, Spain

## Abstract

Cancer accounts for nearly one in four deaths (22.8%) due to noncommunicable diseases globally. The urgency for new effective therapies is worsened by resistance. One strategy is to look for multimodal drugs, which undergo different pathways to achieve selective cytotoxicity. Thiosemicarbazones are known to act as antitumoral compounds through multiple modes of action, and Ni­(II) shares some coordination properties with Pt­(II) but is also accessible to redox reactions such as superoxide dismutation catalysis. We found that, in a list of four thiosemicarbazone Ni­(II) complexes all tested on various cancer cell lines, **Ni4** displayed IC_50_ values down to 5 ± 2 μM and over 8-fold selectivity. We investigated how apoptosis was induced, finding at least two different simultaneous mechanisms both involving the Ni­(II) center: first, entrance of the nucleus and coordination of the minor groove of the DNA, modifying its helicity, and second, disruption of the reactive oxygen species (ROS) balance due to the stoichiometric interactions with radical species and catalytic dismutation of hydrogen peroxide, based on the aliphatic N4 substitution that induces a peculiar two-electron exchange reactivity on the Ni­(II) center. Even if we found that **Ni2** (cytotoxic but nonselective) is as efficient as literature catalase-like mimics (*k*_cat_/*K*_M_: 10 ± 2 M^–1^·s^–1^), **Ni4** treatment hits the upregulation of heme oxygenase (HO-1) and mitochondrial superoxide dismutase (SOD-2). Cyclic voltammetry was used to fully characterize both Ni complexes to investigate the mechanisms of the redox processes associated with electron transfer. Clarifying the cytotoxicity mechanisms, we found that the selectivity is related at least to albumin delivery. Albumin, highly concentrated in the mammalian serum, rapidly seizes the compounds and delivers them selectively to the cancer cells. We found that albumin forms a supramolecular complex with the whole coordination compounds, without sequestering the metal ion, and its affinity is highest for **Ni4** (*K*_b_: 1.5 ± 0.9 × 10^6^ M^–1^).

## Graphical abstract

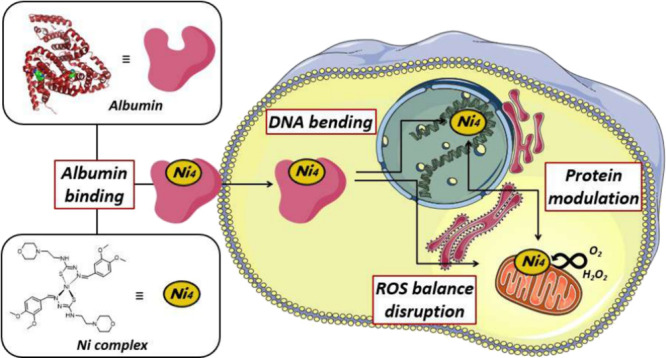

## Introduction

Cancer accounts for almost one in four deaths (22.8%) due to noncommunicable diseases globally. Notably, Europe bears a disproportionately high burden of cancer incidence and mortality, as the continent represents one-fifth of the world’s cancer cases (22.4%) and cancer deaths (20.4%), despite having less than 10% of the global population (9.6%).^1^ In addition, antitumoral drug efficacy is often inhibited by multidrug resistance (MDR).^2^ This underscores the urgent need for continued focus on cancer research and drug discovery. Advancements in cancer treatment have led to several innovative techniques that are transforming patient care such as personalized cancer vaccines,^3^ CAR-T cell therapy,^4^ immune checkpoint inhibitors,^5^ or nanotechnology-based therapies.^6^ Nevertheless, despite the promise of these innovative techniques, traditional compounds remain crucial in cancer treatment, offering proven efficacy and broader accessibility and often serving as the foundation for combination therapies that enhance the effectiveness of newer approaches. Thiosemicarbazones, TSCs (>C=N–NH–C­(S)–N<), and their metallic complexes are well known for their biological activities.^7–12^ Antitumoral applications for TSCs have been explored with great success from the first paper published in 1967 ^13^ to triapine, which has recently been accepted to Phase 3 of clinical trial against cervical and vaginal cancer (NCT02466971).^14^ TSCs display multiple modes of action as, among others, ROS generation, enzyme inhibition, and interaction with DNA. ROS overproduction is related to cell cycle arrest and can trigger DNA damage and promote caspase-9-dependent apoptosis.^15^ This mechanism is exploited by several thiosemicarbazone-based drug candidates: They are often chelated to redox-active metal ions, such as Fe­(III) or Cu­(II), but even as free ligands, their sulfuric moiety is prone to redox reactions, and eventually they can coordinate endogenous metal ions and transport them closer to DNA to highly enhance the ROS damage to the cell. Recent developments^16^ have brought to the attention how thiosemicarbazone complexes induce apoptosis through mitochondrial signaling pathways, and ROS production plays a key role in this mode of action. Enzyme inhibition is another main target for antitumoral-purposed TSCs.^17^ In the past, the main routes were considered to be inhibition of the ribonucleotide reductase^18^ and of the topoisomerase,^19^ but recently, several works highlighted different targets as well such as carbonic anhydrase,^20^ p53,^21^ and COX.^22^ Protein targeting is useful not only to enhance cytotoxicity but to favor selectivity and limit drug resistance as well. Due to their uncontrolled growth, cancer cells absorb a higher amount of transport proteins, like albumin, than healthy cells. In particular, albumin is one of the most abundant proteins in mammalian serum and acts as a host for metal ions and lipophilic molecules.^23,24^ Depending on the strength of its interactions with these guests, it can function as either a sequestrant or a delivery system. It is well established that numerous small molecules bind reversibly to albumin, and several studies have investigated its pharmacokinetic properties in drug delivery.^25–29^ DNA interaction is one of the most common modes of action investigated for TSC cytotoxic activity against cancer cells.^30^ Depending on the metal ion and on the chelating moiety, the interaction between the DNA and TSC complex can be covalent or noncovalent, intercalative, or in the major or minor groove. Such DNA modifications cause the inhibition of cell replication and of protein transcription, and therefore cell death. TSCs with the 3,4-dimethoxy moiety on N1 demonstrated selectivity against cancer cells and higher efficacy in Cu­(II) complexes.^31^ We decided to condense 3,4-dimethoxybenzaldehyde and an array of four thiosemicarbazides differing for the N4 substitution. In addition, we wanted to combine the effect of such ligands with nickel­(II) ion, which displays some interesting characteristics in view of antitumoral therapy:^32^ If properly coordinated, then it can be a reactive center for ROS-mediated modes of action.^33^ Depending on the chelator, it favors square planar coordination geometry, but it is open to the possibility of coordination at the apical positions, which can become attractive for interactions with biomolecules such as proteins or nucleic acids inside the cell.^24^ In summary, we synthesized and characterized four TSC complexes starting from the 3,4-dimethoxy-benzaldehyde group bearing a Ni­(II) center, in place of Cu­(II) and Fe­(III). We found that the lowest cytotoxicity values demonstrated a far greater activity compared to literature^31^ and found evidence about an unprecedented mix of modes of action, which is made possible by the multifaceted nature of the Ni­(II) metal center.

## Results

### Synthesis and Characterization

Chelators (**L1**–**3**) were obtained based on established literature,^31,34,35^ whereas **L4** and complexes **Ni1**–**4** (Scheme 1) are new and were characterized by spectroscopic techniques and by single-crystal X-ray diffraction (see Chart 1, Table 3 of the main text, and Figures S30–S32 of the Supporting Information). Starting from thiosemicarbazides differing for the peripheral N4 group, we could verify the influence of that group on both the chelators and the final complexes. In particular, we used nonsubstituted, methyl, phenyl, and morpholinoethyl (see Scheme 1) N4-substituted thiosemicarbazides. We were therefore able to investigate the role of steric hindrance (**L1** compared to **L3** and **L4**), inductive effect (**L2** and **L4** electron-donating groups compared to the slightly electron-withdrawing phenyl group in **L3**), and polarity (polar **L1** and **L4** compared to **L2** and, most apolar of all, **L3**) in the structural and electronic features of the final compounds.

**Scheme 1:**
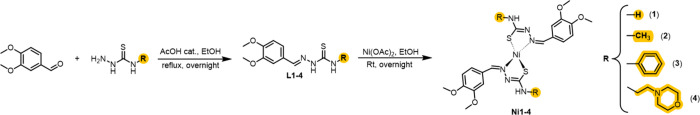
Synthesis of Ligands **L1**–**4** and **Ni1**–**4**

**Chart 1:**
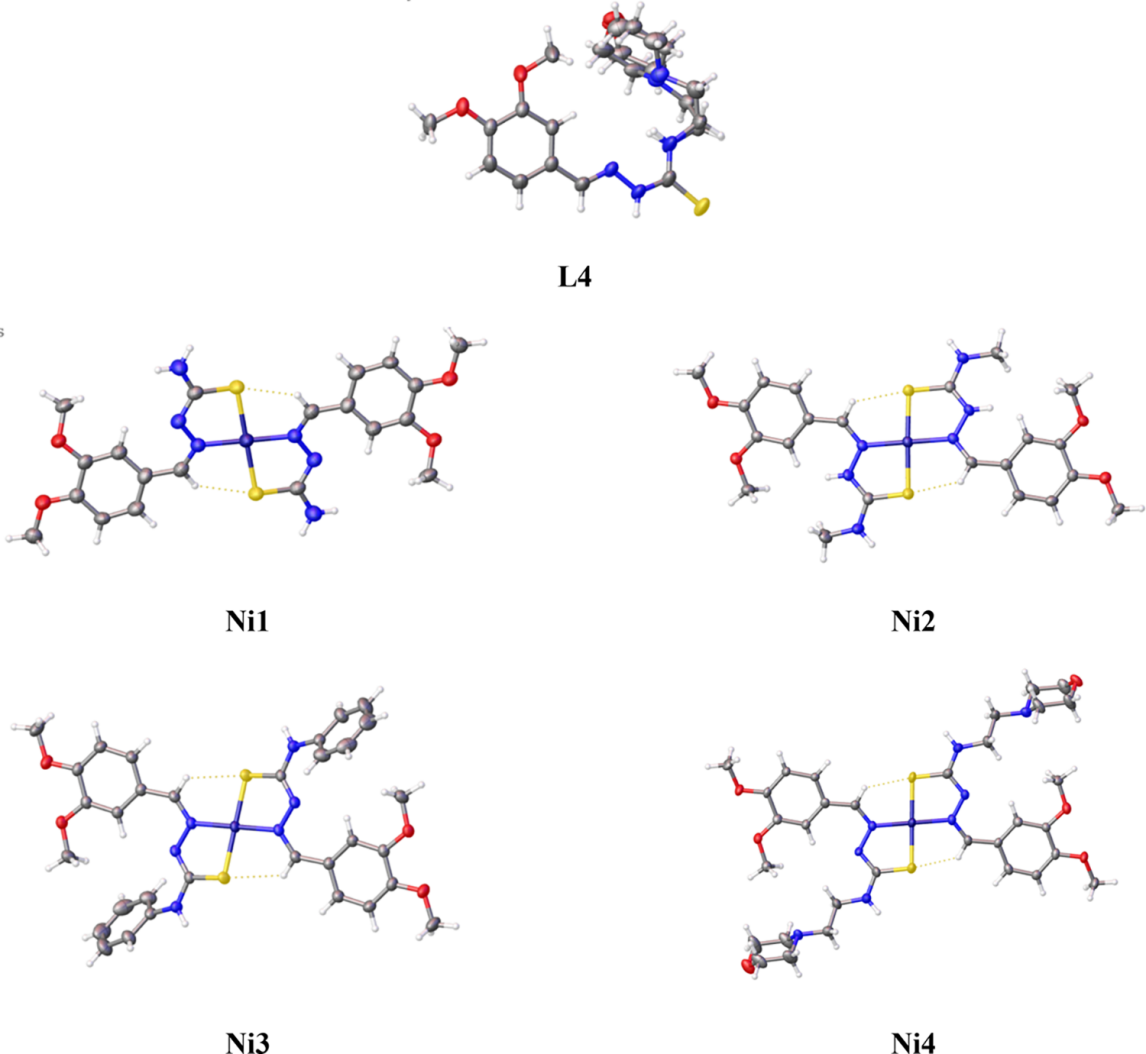
Ball-and-Stick Representation of **L4** and the Complexes

X-ray diffraction allowed us to deepen the structural details of **L4** and complexes **Ni1**–**4** at the solid state (Chart 1). As observed in most thiosemicarbazone structures,^31^ **L4** tends to adopt a conformation that maximizes an internal hydrogen bond between the terminal aminic NH moiety and the *imino* nitrogen. Upon coordination, a rotation occurs around the hydrazine fragment, positioning the sulfur atom in a way that facilitates chelation of the metal center. In the four complexes whose structures have been determined by X-ray diffraction, the chelation mode remains consistent with a square planar geometry with a 2:1 ligand:metal ion stoichiometry and a *trans* configuration on the metal center. However, the extent of distortion in the chelating rings varies (Figures S30–S32).

We then investigated the characteristics of these compounds in the solution. At first, we tested the stability of the complexes and ligands in an aqueous medium simulating *in vivo* conditions, i.e., phosphate-buffered solution (PBS) at pH 7.4, with electrolytes as potassium and sodium chlorides at physiological concentration. Generally, the UV spectra of the ligands remained mostly unvaried, whereas complexes displayed diverse results: If on one side **Ni2** and **Ni3** slightly changed along the 24 h period, then **Ni1** immediately broke and **Ni4** broke only after 1 h (see Figures S33 and S34).

Taking into account the biological data reported from Chart 7 on, we focused on **Ni2** and **Ni4** in solution. We added 100 μL of D_2_O to 500 μL of DMSO-d_6_ NMR samples and incubated for 1 h at 37 °C at atmospheric pressure, in order to have an insight especially on the actual active species (see Chart 2). Comparing the spectra of the complexes before and after the addition of D_2_O and the respective ligands, we hypothesize the following: **Ni2** seems to be overall stable, since the only significant modification on the spectrum is the disappearance of the NH signal due to proton exchange with water; on the contrary, **Ni4** seems to release one of the two ligands, probably forming a square planar complex with two water molecules replacing one **L4** moiety. As a matter of fact, the species remains diamagnetic even after the addition of water, so the coordination configuration does not shift toward a paramagnetic octahedral one. Therefore, we support that in solution, **Ni4** presents itself as a 1:1 complex with a ligand as counteranion.

**Chart 2:**
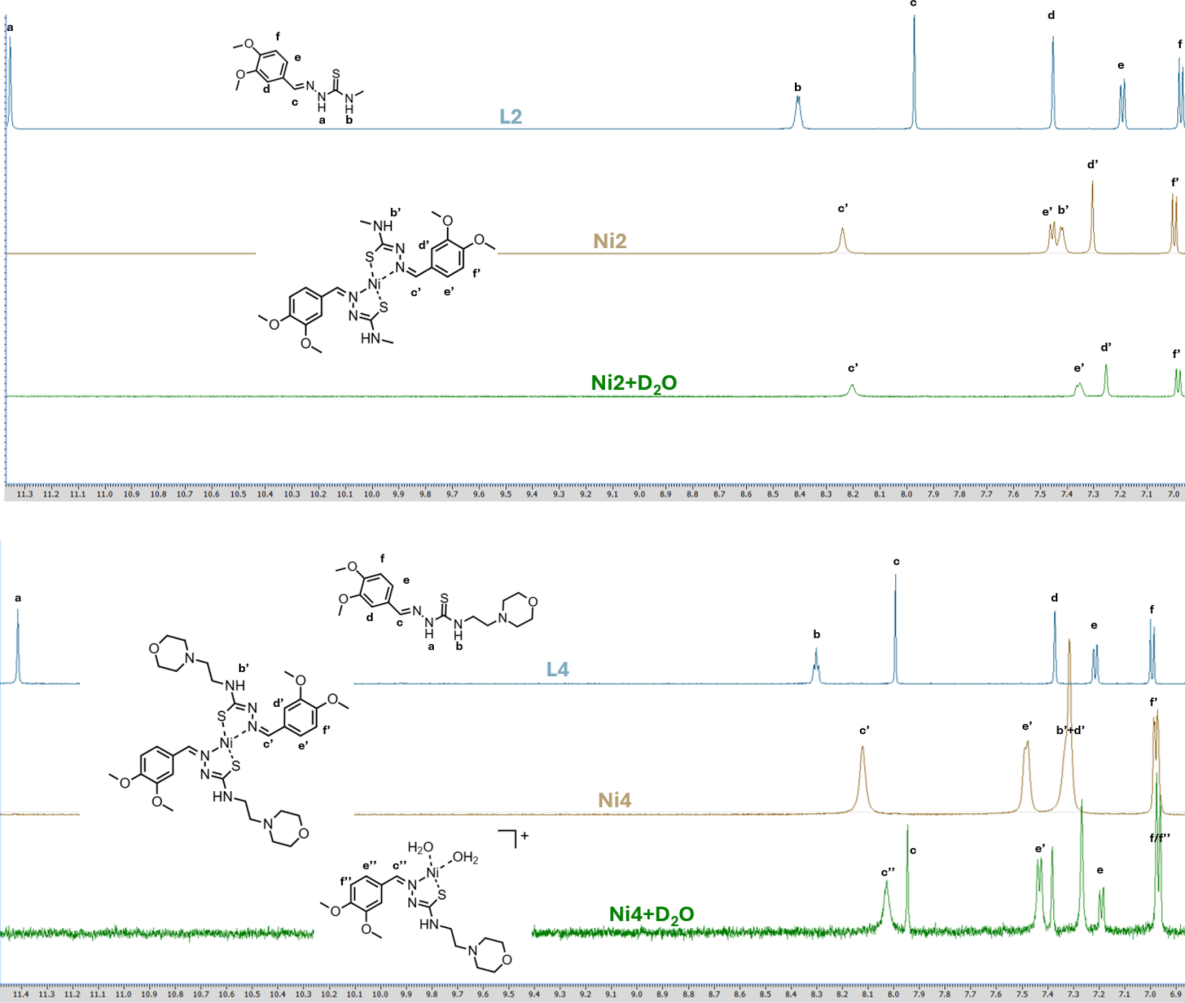
Comparison between the Spectra of **Ni2** and **Ni4** in DMSO-d_6_ before and after the Addition of D_2_O (and Respective Ligands)^a^

a The spectra were taken with a JEOL ECZ600R at 600 MHz, and the compounds were at 10 mM scale concentration.

The stoichiometry of the complexes formed between the ligand (**L1**, **L2**, **L3**, and **L4**) and Ni­(OAc)_2_ was determined using the method of continuous variation (Job’s plot, see Chart 3). Ligand solutions were prepared at a concentration of 10 mM in DMSO, while Ni­(OAc)_2_ were prepared at a concentration of 5 mM in double-distilled water. A series of solutions was prepared by mixing appropriate volumes of the two stock solutions so that the total molar concentration [L] + [Ni^2+^] remained constant at 50 μM in DMSO, while the mole fraction of the ligand (*X*_L_) varied from 0 to 1 in increments of 0.1. The pH of the resulting solutions results to be neutral, around pH 7.0. Each mixture was allowed to equilibrate at room temperature overnight, and the absorbance was recorded. The absorbance at λ = 400 nm was plotted against the mole fraction of the L (*X*_L_). The stoichiometry of the complex was deduced from the position of the maximum in Job’s plot, according to eq 1:

$$\frac{n_{L}}{n_{\mathrm{Ni}}}=\frac{X_{L}}{1-X_{\mathrm{Ni}}}$$ 1

**Chart 3:**
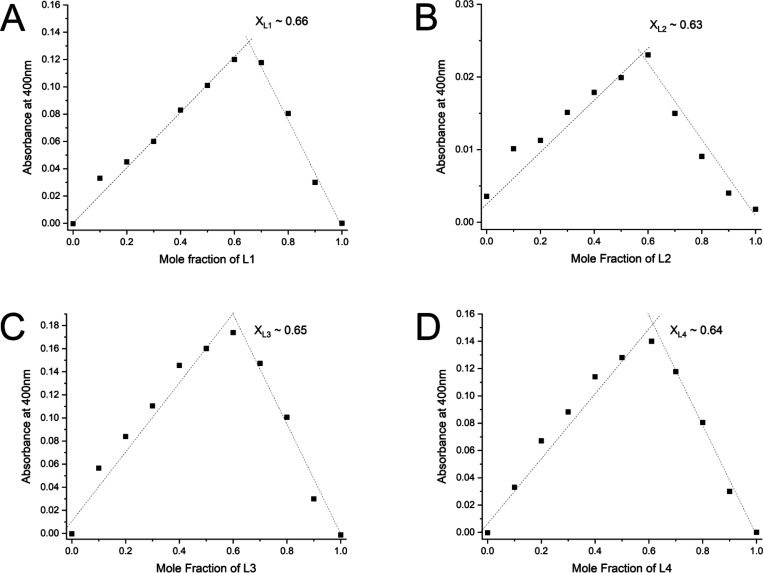
Job’s Plot (Method of Continuous Variation) for the Complexation between ligand **L1** (A), **L2** (B), **L3** (C), and **L4** (D) with Ni­(II) at λ = 400 nm^a^

a Total molar concentration [L] + [Ni^2+^] = 50 μM, in DMSO at pH 7.0; *T* = 25 °C.

The stoichiometry of the complexes in solution was confirmed to be 2:1, the same as that reported with the XRD characterization.

Second, we tested their stability in the presence of reactive oxygen species, in particular O_2_^·–36^, ^·^OH,^36^ and H_2_O_2_,^36^ which are the most important reactive oxygen species present in human cells (see Chart 4 and Figures S35–S36).

**Chart 4:**
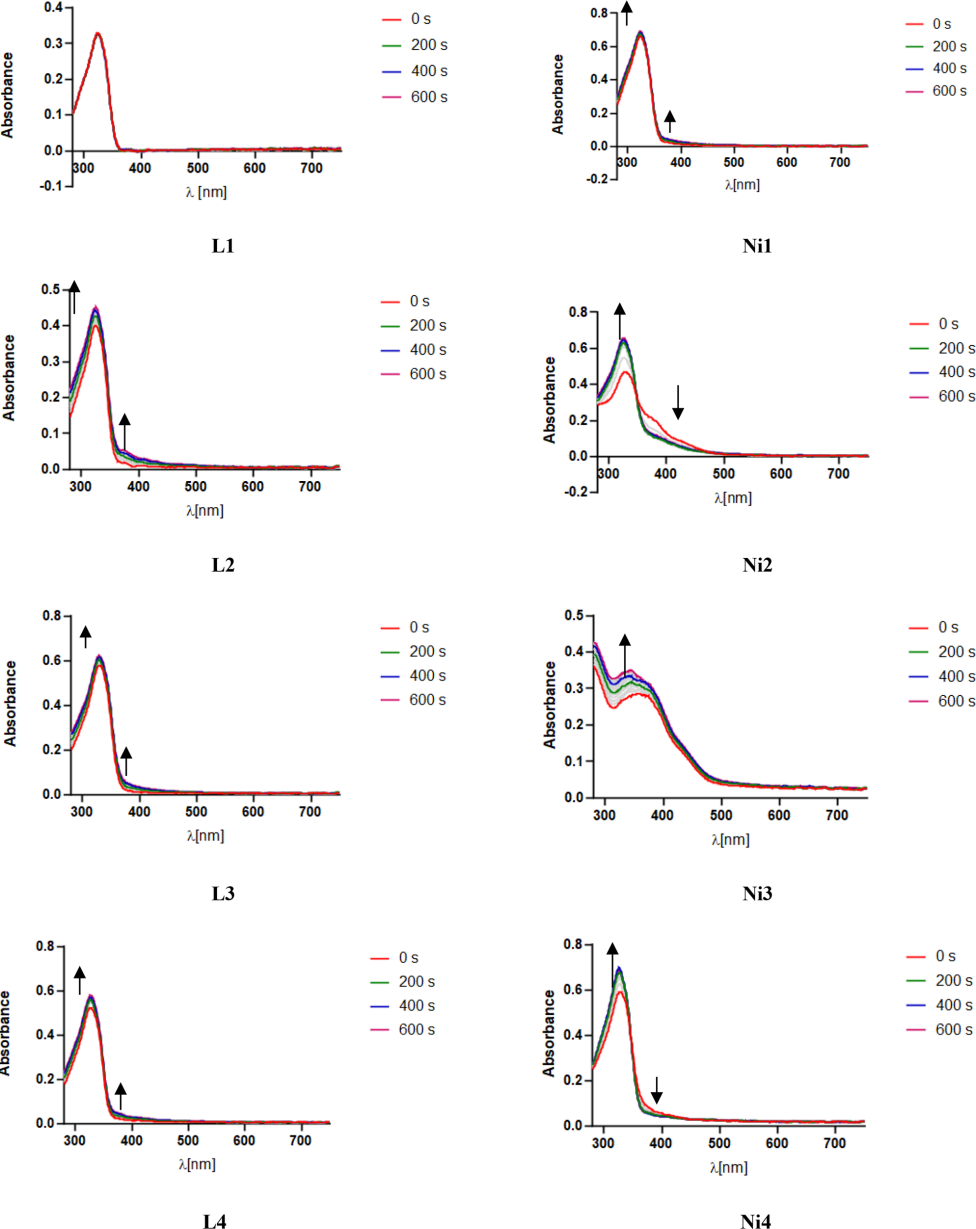
Time-Dependent Electronic Spectra of Ligands and Complexes in the Presence of OH Radical Generated *In Situ* via Fenton Reaction^a^

a In a 45.4 μM solution of FeSO_4_ in water is added a DMSO solution of target compound at 9.1 μM in the final mixture. To this solution, H_2_O_2_ was added to achieve a final concentration of 454 μM. The UV–visible spectra were recorded at every 40 s for around 10 min.

Finally, we tested their reactivity with diphenylpicrydrazyl (DPPH),^36,37^ which is a bulky *N*-radical that enables to use a common colorimetric experiment to estimate the capacity of the target compounds to interact with bulky radicals as lipid peroxides (usually defined as LPO or ROO^·^) (see Chart 5 and Experimental Section). The DPPH assay result is expressed as radical scavenging activity (RSA%), which is proportional to the capacity of a compound to quench this large and stable radical: In general, ligands reported higher RSA% values compared to complexes, which still maintained a valuable reactivity toward DPPH. In comparison, complexes seemed to be degraded more easily by the ^·^OH radical than ligands: In fact, the absorption bands of the complexes, especially the charge transfer bands of **Ni2** and **Ni4**, changed a lot more than the corresponding chelators.

**Chart 5:**
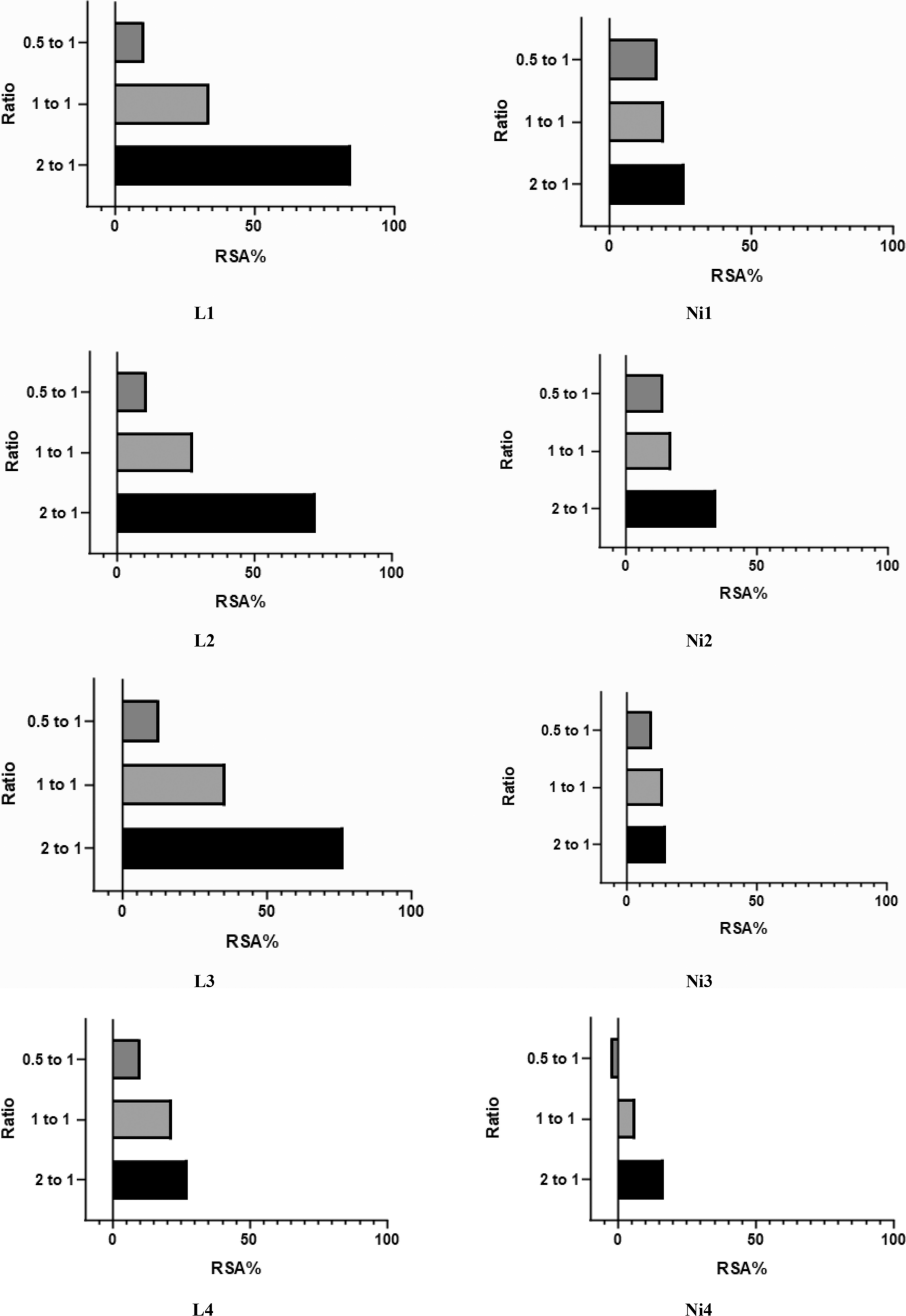
Chart Reporting the Radical Scavenging Assays at Different Compound/DPPH Ratios^a^

a 500 μL of a MeOH 100 μM solution of DPPH in methanol was mixed with 500 μL of DMSO solutions of the compounds at different ratios and incubated for 30 min at 37 °C in the dark. Absorbances of radical DPPH, molecular DPPH, compounds, and compounds/DPPH mixtures were read at 517 nm and used to calculate the radical scavenging activity (RSA%) using eq 1).

Time-dependent H_2_O_2_ degradation assay and pseudotitration with O_2_^·–^ apparently resulted in slight to negligible modifications of the UV–visible spectra. As exception, **Ni1** almost instantly broke with H_2_O_2_, but it must be probably due to its already cited high instability in aqueous media (compare Figures S33–S36).

### Cyclic Voltammetry Characterization

The voltammetric characterization of complexes **Ni1**–**4** was carried out in acetonitrile (ACN) solution, although complete solubility was not achieved, by analyzing the saturated solutions; in fact, lower concentrations were preferred over the use of alternative solvents because ACN provided a higher resolution of the CV peaks compared to DCM or DMF (see Chart 6 and Table S1).

**Chart 6:**
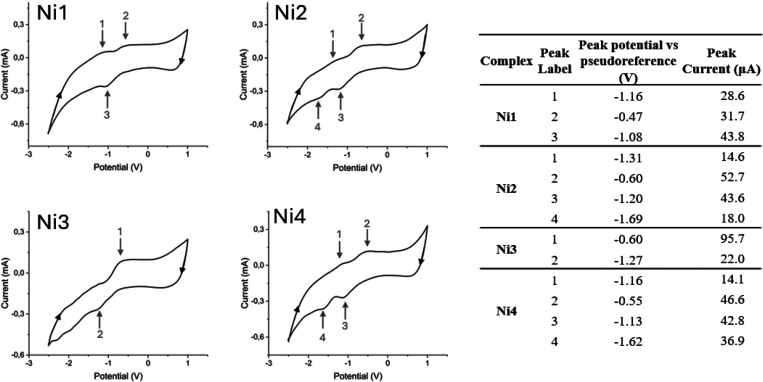
On the Left, CV Pattern of the Ni­(II) Complexes, Acquired on Carbon Screen-Printed Electrodes in Acetonitrile Solutions; Scan Rate = 0.1 V/s^a^

a Complex concentrations ranging from 0.1 to 1 mM in ACN solutions, also containing 0.1 M tetrabutylammonium tetrafluoroborate as the supporting electrolyte. On the right are the peak potentials and currents of the complexes **Ni1-4**. See Table S1 and the Experimental Section for further details.

The CV patterns of **Ni2** and **Ni4** are ascribable to a quasi-reversible double electron transfer process, in which Ni­(II) is oxidized to Ni­(III) (labels 1) and subsequently to Ni­(IV) (labels 2) in the forward scan in the anodic direction, while in the backward scan, we recognize the corresponding Ni­(IV)→Ni­(III) (labels 3) and Ni­(III) → Ni­(II) (labels 4) reductions, coherently with the literature.^38^ Furthermore, the ratios of the absolute values of the anodic over cathodic peak currents (peak current 1/peak current 4 and peak current 2/peak current 3) are almost always close to 1, thereby confirming the quasi-reversibility of the electrode processes. **Ni1**, instead, exhibited an analogous CV pattern for the forward scan (anodic direction), while displaying a single cathodic peak (label 3) at a potential analogous to that observed for the Ni­(IV) → Ni­(III) reduction of **Ni2** and **Ni4**. This finding indicates a reduced degree of reversibility of the electrode-oxidation processes. Interestingly, **Ni3** reveals a different pattern, exhibiting a single anodic peak in the forward scan, which is positioned at potential values congruent with those associated with the Ni­(III) → Ni­(IV) oxidation in the other complexes. Furthermore, the electrodic process occurs starting from Ni­(II) in a single two-electron transfer process, as confirmed by a peak current value double compared to the other compounds studied. In addition, this behavior is reproduced in the cathodic backward scan, but with a reduced degree of reversibility, as highlighted by a ratio between the anodic and cathodic currents very different from 1 (peak 1 current/peak 2 current = 4.35). These data suggest an electron transfer coefficient of >0.5 for the forward anodic process, which may be ascribable to a high degree of thermodynamic stabilization of Ni­(IV) in **Ni3**. From this CV characterization, we interestingly conclude that **Ni2** and **Ni4** seem to be the best candidates to exchange electrons in a catalytic fashion. In addition, we compared these potentials to the literature to understand their electronic behavior toward ROS.

### Computational Study

We performed computational studies to support voltammetric and redox characterization. Both the crystallographic data of the complexes and the optimization made by computation are in excellent agreement with the geometries of the complexes, which validate the chosen method (see the Supporting Information). We found that the energy gap between the HOMO and LUMO is relatively large, indicating that the neutral complexes are quite stable and are not very susceptible to electronic excitation. Even as monocationic charged complexes, they exhibit good stability and poor reactivity in electronic terms with the energy difference decreasing slightly. This suggests that the complexes become more reactive and easier to excite while still maintaining a certain level of stability. On the contrary, when the complexes carry a double positive charge, the HOMO–LUMO gap decreases significantly. As shown in Table 1, these values vary considerably depending on whether the substituents on the ligands are electron donors or electron acceptors. See Figure S37 for the images of the molecular orbitals and further experimental details.

**Table 1:** Computational Energy Values Calculated for **Ni1**–**4** Complexes in Different Charge States (Neutral, Cationic +1, and Cationic +2)^a^

energies of MO (eV) vacuum			
	HOMO	LUMO	HOMO–LUMO gap (kcal/mol) vacuum
**Ni1 neutral**	–0.194820	–0.70436	2.87
**Ni**1 (+1)	–0.302498	–0.196157	2.45
**Ni**1 (+2)	–0.411252	–0.391421	**0.46**
**Ni2 neutral**	**–** **0.188294**	–0.075034	2.61
**Ni**2 (+1)	–0.306535	–0.202309	2.40
**Ni**2 (+2)	–0.404942	–0.384304	**0.48**
**Ni3 neutral**	–0.199986	–0.078259	2.81
**Ni**3 (+1)	–0.296915	–0.186923	2.54
**Ni**3 (+2)	–0.387926	–0.373762	**0.33**
**Ni4 neutral**	**–** **0.187059**	–0.059208	2.95
**Ni**4 (+1)	–0.302844	–0.196879	2.44
**Ni**4 (+2)	–0.382826	–0.371168	**0.27**

a Geometries were optimized in the gas phase using the B3LYP/6-31+G­(**) level of theory. Frequency calculations (at 298.15 K) at the same level of theory were used to confirm the nature of all stationary points as minima and provided values for computing free energies.

### Cytotoxicity Assays

At first, we evaluated the effect of ligands and complexes on various cancer cell lines: adenocarcinomic human alveolar basal epithelial cells A549, human acute promyelocytic leukemia cell line HL60, human colorectal adenocarcinoma cell line HT29, and human epithelial mesothelioma cell line H2052 ([Fig cht7]). We screened **L1**–**4** and **Ni1**–**4** on these cell lines at 100 μM for 24 and 48 h to select the compounds with relevant efficacy.

**Chart 7:**
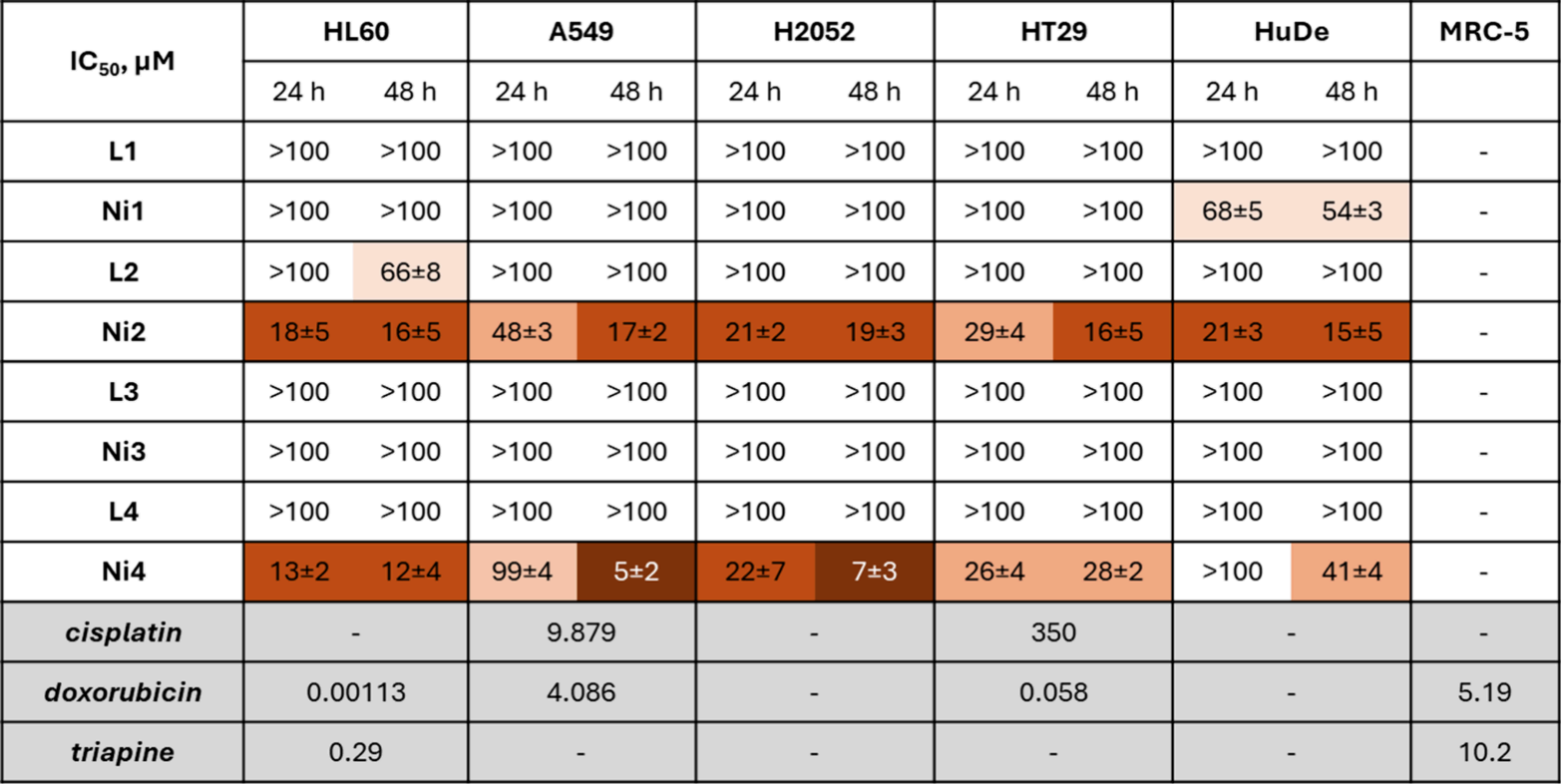
IC_50_ Values at 24 and 48 h for HL60, A549, H2052, and HT29 Cancer Cell Lines and for the HuDe Healthy Cell Line^a^

a The results are shown as means ± SD. Available cisplatin, doxorubicin, and triapine cytotoxicity values are reported for comparison (references in the Main Text).

Subsequently, the compounds that showed an inhibition of proliferation were used at different concentrations (0.1–100 μM) and we calculated the inhibitory concentration (IC_50_) at different times (24 and 48 h). Values for cisplatin (A549 ^39^ and HT29 ^40^), doxorubicin (HL60,^41^ A549,^42^ HT29,^43^ and MRC-5 ^44^ as healthy control), and triapine (HL60 ^45^ and MRC-5 ^14^) are reported for comparison.

Ligands, **Ni1**, and **Ni3** did not inhibit the proliferation of the four cancer cell lines studied up to a concentration of 100 μM, while treatment with **Ni2** and **Ni4** complexes significantly inhibited cell growth and affected cell viability in both concentration- and time-dependent ways (see Chart 7). The selectivity of the compounds was tested using HuDe as a healthy cell comparison: at 48 h, compared to A549, **Ni4** showed a selectivity index (SI) of 8.2, whereas the effect of **Ni2** was comparable to all the other cell lines, revealing a negligible selectivity (see Figure S38 for the IC_50_ fit curves).

The apoptotic nature of the cell death process was assayed for the most active compounds, **Ni2** and **Ni4**, and related ligands on A549 cells (Chart 8). A549 cells treated with **Ni2** and **Ni4** displayed a significantly higher caspase-3 activity compared to free ligands. This activity increased progressively over time, becoming more pronounced at 48 and 72 h, where **Ni4**-treated cells show the highest levels of caspase-3 activation. Treatment with **Ni2** induces the upregulation of p53 at 24 h only, even if in a nonsignificant manner compared to its own ligand, while treatment with **Ni4** can induce the expression of p53 for all the times considered in agreement with the caspase-3 activation.

**Chart 8:**
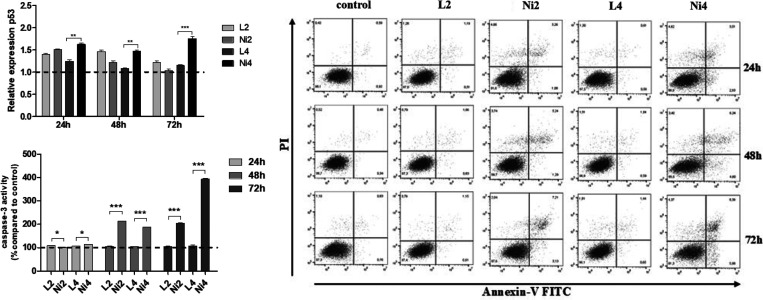
On the Left, Data Related to Apoptosis Are Reported for **Ni2** and **Ni4** (the Most Active Compounds of the List) and Related Ligands: p53 Gene Expression and Caspase-3 Activity in A549 Cells Treated with IC_50_ Concentrations of Compounds for 24, 48, and 72 h^a^

a Values are mean ± SD of three separate experiments, each carried out in triplicate (dashed line = CTR). Statistical significance is indicated as follows: **p < 0.01, ***p < 0.001. On the right, Annexin V/PI flow cytometric analysis of cell death in A549 cells treated with ligands L2 and L4, and their corresponding Ni­(II) complexes (L2Ni and L4Ni), compared with untreated control cells. Dot plots represent apoptosis and necrosis levels after 24, 48, and 72 h of treatment. Annexin V FITC is shown on the *x*-axis (early apoptosis), and PI staining on the *y*-axis (late apoptosis/necrosis).

Flow cytometric analysis of apoptosis using the Annexin V/PI assay confirmed the presence of apoptotic cells following treatment with the Ni complexes. After 72 h of treatment, the percentage of apoptotic cells reached 10.34 and 14.37% in the samples treated with **Ni4**, whereas the corresponding ligands did not induce apoptosis.

### Albumin-Mediated Delivery

To prove the *in vivo* toxicity of the compounds, we employed the *Galleria mellonella* survival test.^39^ Unexpectedly, all of the compounds, even the ones reported to be harmless toward cancer cells (compare Chart 7 and Figure S39) caused larval death. In addition, we noted that larvae often reported necrotic development in the area below the point of injection. This is probably ascribable to precipitation, which was not evident in the culture medium of human cells. We then hypothesized that something else was needed, present in human cell culture medium, absent in insects, and able to convey the compounds and avoid their precipitation: Transport proteins fit this role, and among them, we decided to study albumin, absent in insects as *G. mellonella*,^47^ as a model.

We employed bovine serum albumin (BSA) as it is very close to human serum albumin (HSA)^48,49^ and way cheaper.^50^

We performed circular dichroism (CD) and fluorescence experiments on solutions of BSA and the compounds, to reveal the nature of the interactions. A preliminary CD experiment revealed the Cotton effect of the BSA-Ni­(II) complex between 380 and 525 nm (see Figure S40). To reveal whether the Ni­(II) core was sequestered by the BSA, we performed CD on solutions of BSA and **Ni1**–**4** complexes under the same conditions. We found that the Cotton effect given by the BSA-Ni­(II) complex was absent from all of the spectra, indicating that Ni­(II) was not sequestered by BSA.

Fluorescence titrations were possible due to the presence of two tryptophan residues (W134 and W213; see Chart 9a^41^) close to two binding sites, resulting in fluorescence quenching upon complexation of guests. After elaboration with Stern–Volmer standard and modified plots (see Chart 9), the experiment resulted in a set of thermodynamic and kinetic parameters. The quenching kinetic constants (*k*_q_) reported that all the compounds experienced a static quenching, therefore being complexed by albumin. In addition, binding constants (*K*_b_) expressed a higher affinity between complexes and BSA, the lowest being 9.2 × 10^2^ for **L3** (harmless to cancer cells) and the highest 1.6 × 10^6^ for **Ni4** (which is the most cytotoxic and selective).

**Chart 9:**
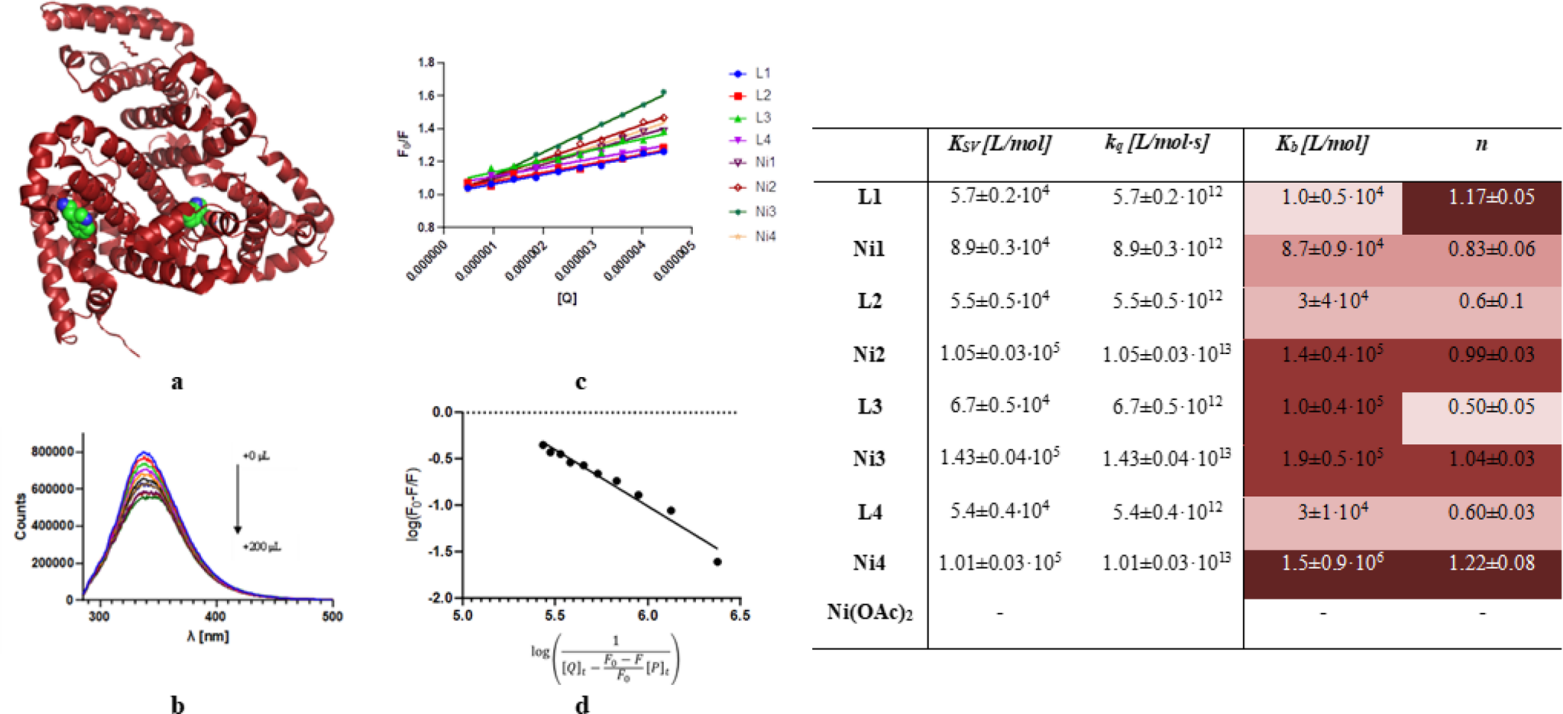
(a) X-ray Diffraction Structure of Bovine Serum Albumin (PDB: 4F5S), in which the Fluorescent Residues W213 and W134 Are Highlighted; (b) Fluorescence Titration Profile of Ni4 (as Example) to BSA in Buffered Solution; (c) Stern–Volmer Plot, with [*Q*] in M; (d) Modified Stern–Volmer Plot with [*Q*] and [*P*] in M^a^

a BSA was dissolved in 2.5 mL of pH 7.4 buffered solution ([HEPES]: 50 mM; [NaCl]: 0.1 M; adjusted to pH: 7.4 using NaOH 2 M) to a 2.5 μM concentration. Stock solutions of compounds were prepared in DMSO at 60 μM. After having recorded the spectra of the starting solution of BSA, more than 10 aliquots of 20 μL each were added and then as many spectra were recorded. On the right are the resulting kinetic and thermodynamic parameters. Kinetic quenching constants (*k*_q_) are all over the static quenching threshold; therefore, the lowering in fluorescence intensity is due to a complexation of the compounds. Thermodynamic parameters, i.e., binding constants (*K*_b_) and the estimated number of binding sites (*n*), are reported. For other data and results, see Unit S4.3.

Combining these two experiments, we deduced that BSA-complexed compounds **Ni1**–**4** supramolecularly without sequestering Ni­(II). The N4 group displayed a relevant influence on the outcome, with the most polar and bulky ethylmorpholino-substituted **Ni4** being the most feasible for this interaction and **Ni1** being the least. Since the phenyl-substituted **Ni3** was the second most complexed compound, size seemed to be less influential than the number of interactions, which favored the complexation with no dependency on polarity.

### DNA Interaction Pathway

DNA is the classic target for inducing cytotoxicity, as modifications to DNA can lead to damage in cell replication and regulation, which are fundamental to the mode of action of the most famous antitumor metal-based drug: cisplatin.^51^ With **Ni1**–**4** being all square planar, we expected that they would have been optimal to interact with DNA. We first performed CD and ethidium bromide (EthBr) displacement fluorescence experiments using calf thymus DNA (CT-DNA) and then compared the results with CD experiments on the DNA extracted from the A549 cells treated with **Ni4** (Charts 10, 11, and 12).

**Chart 10:**
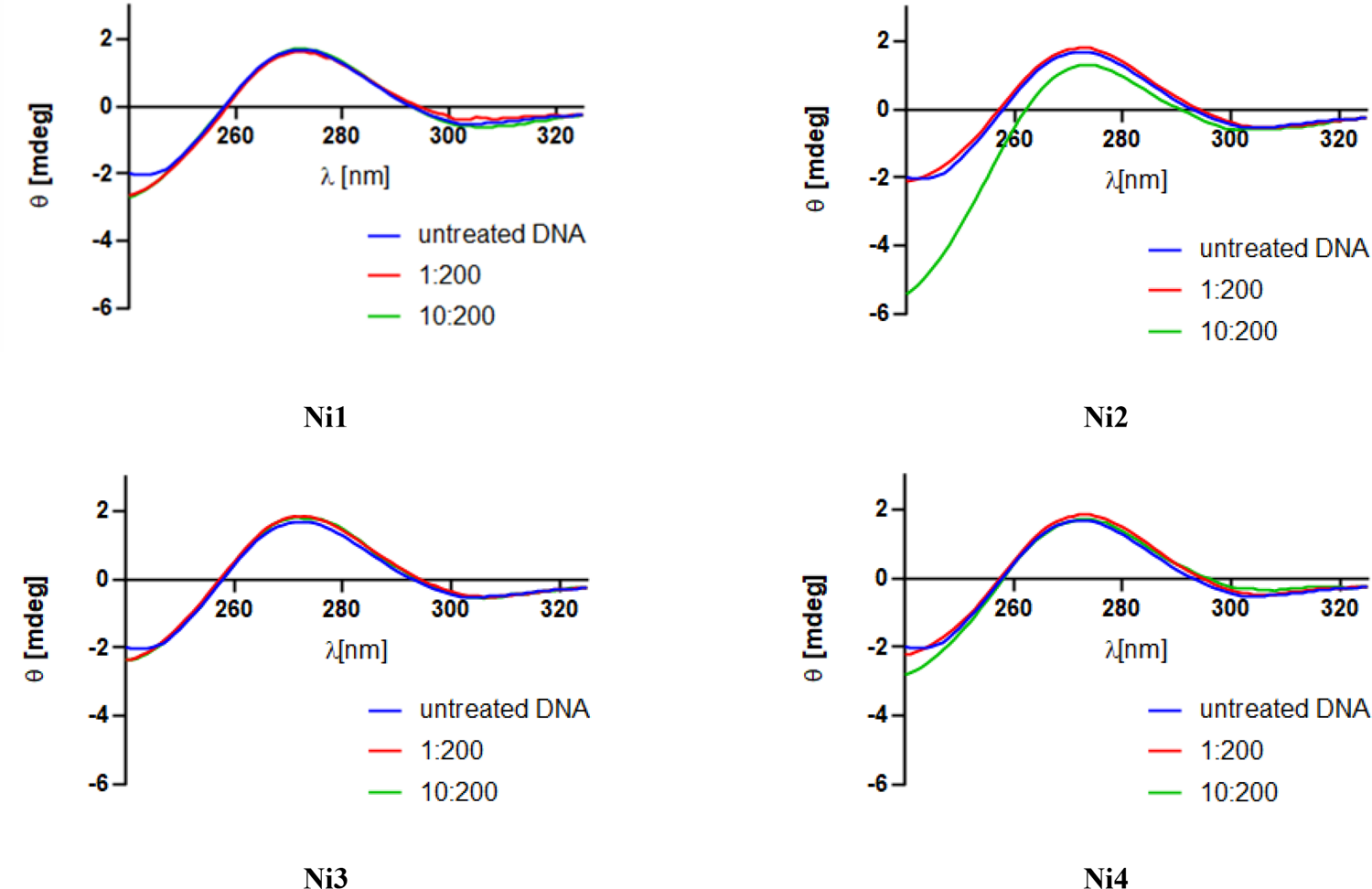
Circular Dichroism Profile before and after Addition of Target Complexes of CT-DNA ([bp] = 37.6 μM) at Different Ratios in PBS Buffer (pH 7.4, [PBS] = 10 mM, [NaCl] = 137 mM, [KCl] = 2.7 mM)

**Chart 11:**
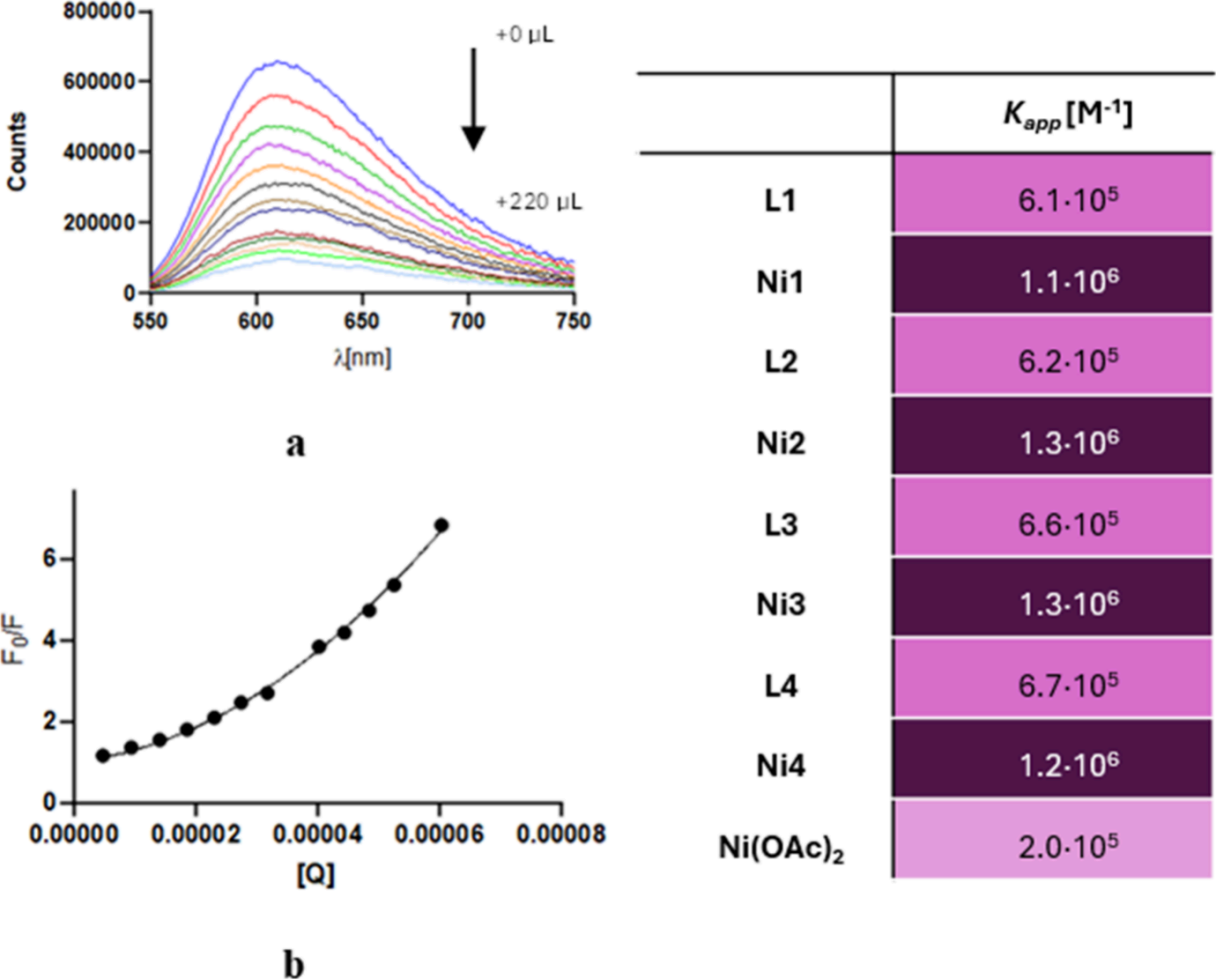
(a) Ethidium Bromide-DNA Adduct Emission at Increasing Complex Concentration (**Ni4**, as Example), Corrected for the Dilution Factor; (b) *F*_0_/*F* to [*Q*] Quadratic Plot^a^

a Solvent system: pH 7.4 buffered solution ([HEPES]: 50 mM; [NaCl]: 0.1 M; adjusted to pH: 7.4). DNA was at [bp] = 54.5 μM (initial volume of 2.5 mL). [EthBr] = 2.6 μM. Ligands, complexes, and nickel acetate stock solutions were prepared in DMSO at concentration of 600 μmol/L. Subsequently, we added 20 μL aliquots of compound/nickel acetate solution directly inside the cuvette more than 10 times. On the right, the estimated apparent constant values (*K*_app_) of each compound and DNA.

**Chart 12:**
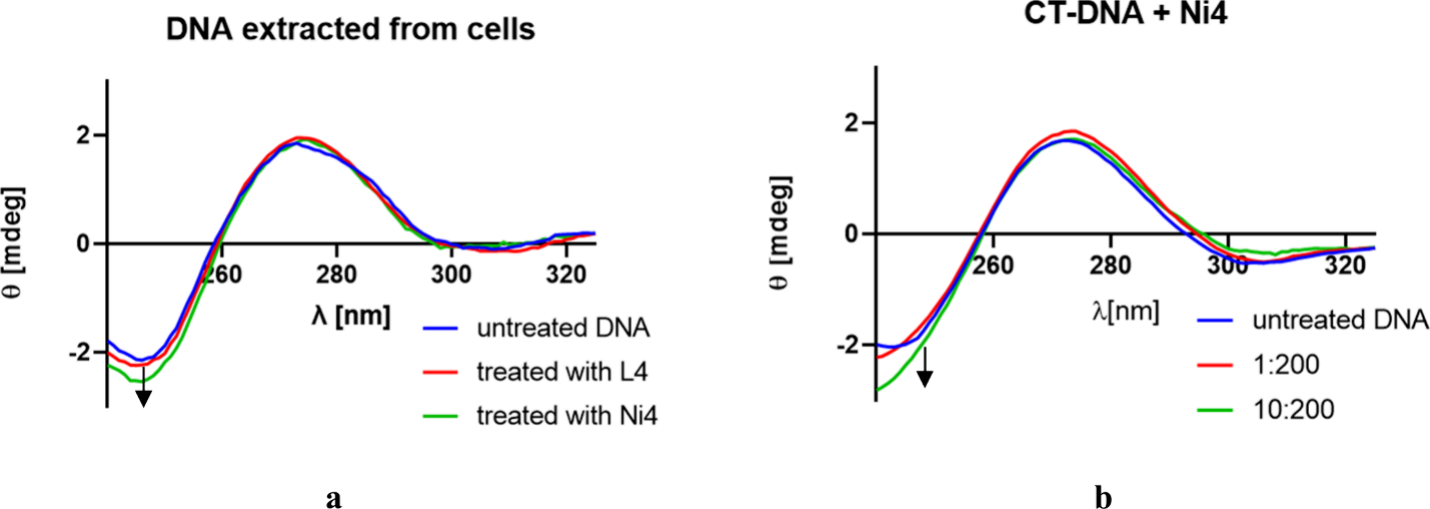
(a) CD Spectra of the DNA Extracted from A549 cells, Untreated and Treated with either **Ni4** or **L4** at a Concentration of 5 μM (Corresponding to the IC_50_ Value of **Ni4**)­^a^

a Base pair concentration normalized at 0.15 mM in buffered HEPES solution ([HEPES]: 50 mM; [NaCl]: 0.1 M; adjusted to pH: 7.4 using 2 M NaOH). *A*_260_/*A*_280_ = 1.8. (b) For comparison, CD spectra of CT-DNA ([bp] = 37.6 μM) at different ratios in PBS buffer (pH 7.4, [PBS] = 10 mM, [NaCl] = 137 mM, [KCl] = 2.7 mM).

EthBr displacement titration with fluorescence resulted in apparent stability constants (*K*_app_), expressing the affinity of the compounds for DNA specifically via intercalation or minor groove binding: All the complexes share *K*_app_ values between 1.1 and 1.3 × 10^6^ M^–1^, whereas ligands express lower values, being **L1** the least interactive with a *K*_app_ of 6.1 × 10^5^ M^–1^. At the CD, only **Ni2** and **Ni4** caused evident differences on the Cotton effect bands: In particular, the negative band at 240 nm enhanced in intensity after incubation of CT-DNA with these complexes (Chart 10). This modification is due to a change in helicity, related to groove binding rather than intercalation or backbone intermolecular interaction, which is coherent with the EthBr displacement assay and a minor groove binding of these complexes (Chart 11 and Figure S42). To understand if this behavior is retained *in vitro*, so is one of the causes of the cytotoxicity, we extracted the DNA of A549 cells treated with **Ni4** and **L4** (and untreated, as comparison) and obtained the CD spectra (Chart 12).

As expected, DNA of cells treated with **L4** was almost superimposable with that of untreated cells, whereas **Ni4** caused a conformational change qualitatively comparable to the deformation experienced by CT-DNA after incubation with the same compound.

### ROS-Mediated Pathway

After a preliminary set of assays using electronic spectroscopy (see above), we performed experiments directly on the A549 cancer cells to discover whether oxidative stress and ROS concentrations were involved in the mode of action of **Ni2** and **Ni4**.

The formation of intracellular ROS was first revealed using 2,7-dichlorodihydrofluorescein diacetate (DCFH-DA), a nonpolar and nonfluorescent compound that diffuses into the cytoplasm where intracellular esterases cleave the acetate group to yield polar, nonfluorescent 2,7-dichlorofluorescein (DCF), whereas its reaction with ROS forms a highly fluorescent two-electron oxidation product. This experiment revealed that, in comparison with controls, **Ni2** and **Ni4** enhanced oxidative stress of about 40 and 30%, respectively, compared to the untreated control (see Chart 13), whereas ligands were comparable to the control.

**Chart 13:**
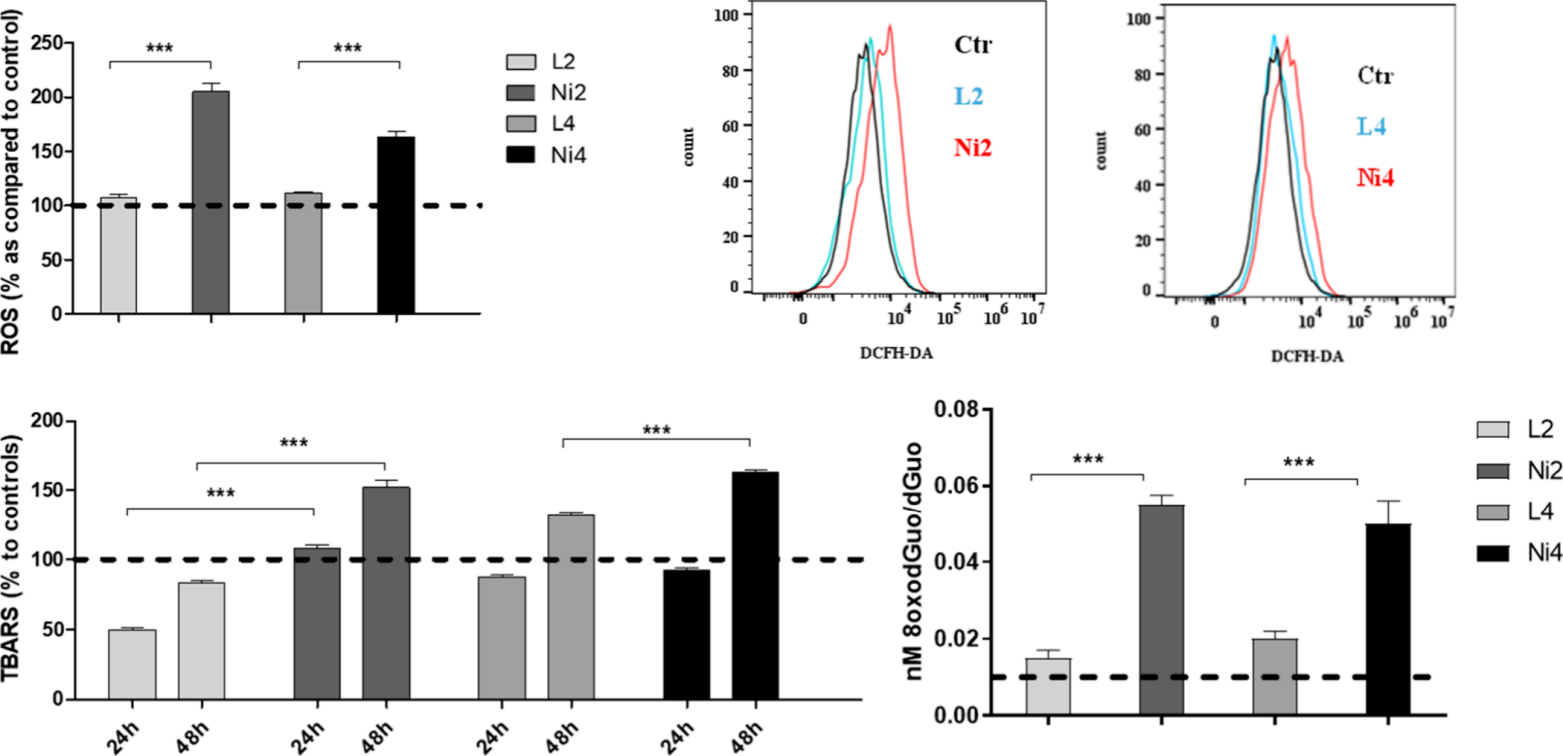
Indicators of the Oxidative Stress inside the Cellular Environment^a^

a Above-left are effects on ROS productions during A549 exposure to IC_50_ concentrations of the tested compounds. The dotted line represents the control. Values are mean ± SD of three separate experiments, each carried out in triplicate. Percentage vs control = (sample value/control value) × 100. Significantly different from untreated control: ***p < 0.001. Above-right are flow cytometry-based analysis of ROS production in A549 cells treated with compounds for 90 min. Results are presented as cytogram overlay (**Ni2** and **Ni4**). Below-left are effects on TBARS, during A549 exposure to IC_50_ concentrations of the tested compounds. Values are mean ± SD of three separate experiments, each carried out in triplicate. The dotted line represents the control. Percentage vs control = (sample value/control value) × 100. Significantly different from untreated control: ***p < 0.001. Below right are effects on guanosine oxidation during A549 exposure to IC_50_ concentrations of compounds. The dotted line represents the control. Values are mean ± SD of three separate experiments, each carried out in triplicate. Significantly different from untreated control: ***p < 0.001.

Oxidative stress in A549 cells due to **L2**, **L4**, **Ni2**, and **Ni4** was further assessed using thiobarbituric acid reactive substances (TBARS) assay. This test revealed that **L2** is not involved in ROS-related mechanisms, whereas **L4** and both **Ni2** and **Ni4** induce lipid peroxidation at 48 h. Coherently with DCFH-DA assay, anyway, **Ni2** and **Ni4** seemed to be both more effective than **L4**. Therefore, the ethylmorpholine substitution favors oxidative stress, but Ni­(II) is determinant as well (see Chart 13).

Mass spectrometry analysis revealed a significant increase in oxidized DNA bases in cells treated with the compounds. These results are consistent and complementary to the intracellular ROS data, providing stronger evidence for the involvement of oxidative stress in the mechanism of action (see Chart 13).

Accordingly, we tested the concentration of H_2_O_2_ in cell supernatant using Amplex Red Kit: unexpectedly, we found that cells treated with **Ni2** and **Ni4** did not have not only higher levels of H_2_O_2_ than cells treated with ligands and untreated, but even lower. Therefore, it seemed that the complexes consumed H_2_O_2_ rather than produced it (Chart 14).

**Chart 14:**
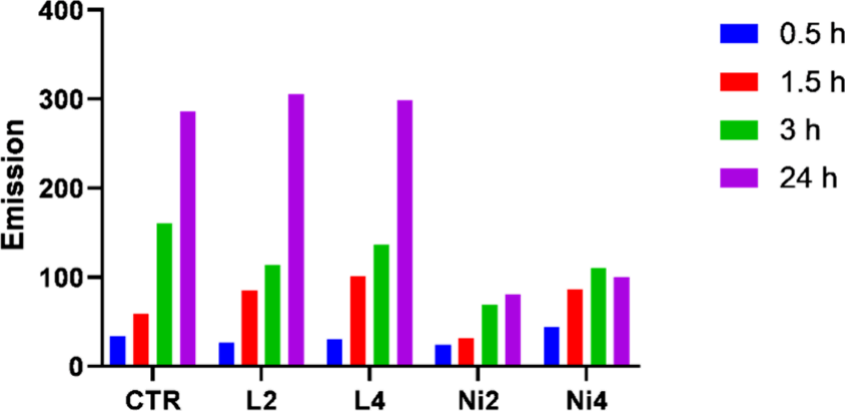
Amplex Red Results, Expressing the H_2_O_2_ Concentration in A549 Cell Supernatant^a^

a The detection limit of the assay was 0.1 μM; cells were seeded in six-well plates without phenol red medium, and after 24 h from seeding, they were treated with **L2**, **L4**, **Ni2**, and **Ni4**; H_2_O_2_ was measured in the supernatant cell collected after 30 min, 90 min, 3 h, and 24 h.

We then studied whether **Ni2** and **Ni4** were directly involved in H_2_O_2_ consumption and if it is stoichiometric or catalytic. We therefore performed experiments with a Clark-type oximeter:^53^ They consisted of dissolving the complexes in aqueous medium buffered at 7.4 and, after having added H_2_O_2_ at increasing concentrations at each trial, monitoring the O_2_ concentration in a time-dependent fashion. We found that both **Ni2** and **Ni4** consume catalytically H_2_O_2_ (see Chart 15). Since we hypothesized a catalase-mimic behavior, i.e., catalyzing H_2_O_2_ dismutation,^54^ we fitted the data using the Michaelis–Menten equation.

$$2H_{2}O_{2}\to 2H_{2}O+O_{2}$$

**Chart 15:**
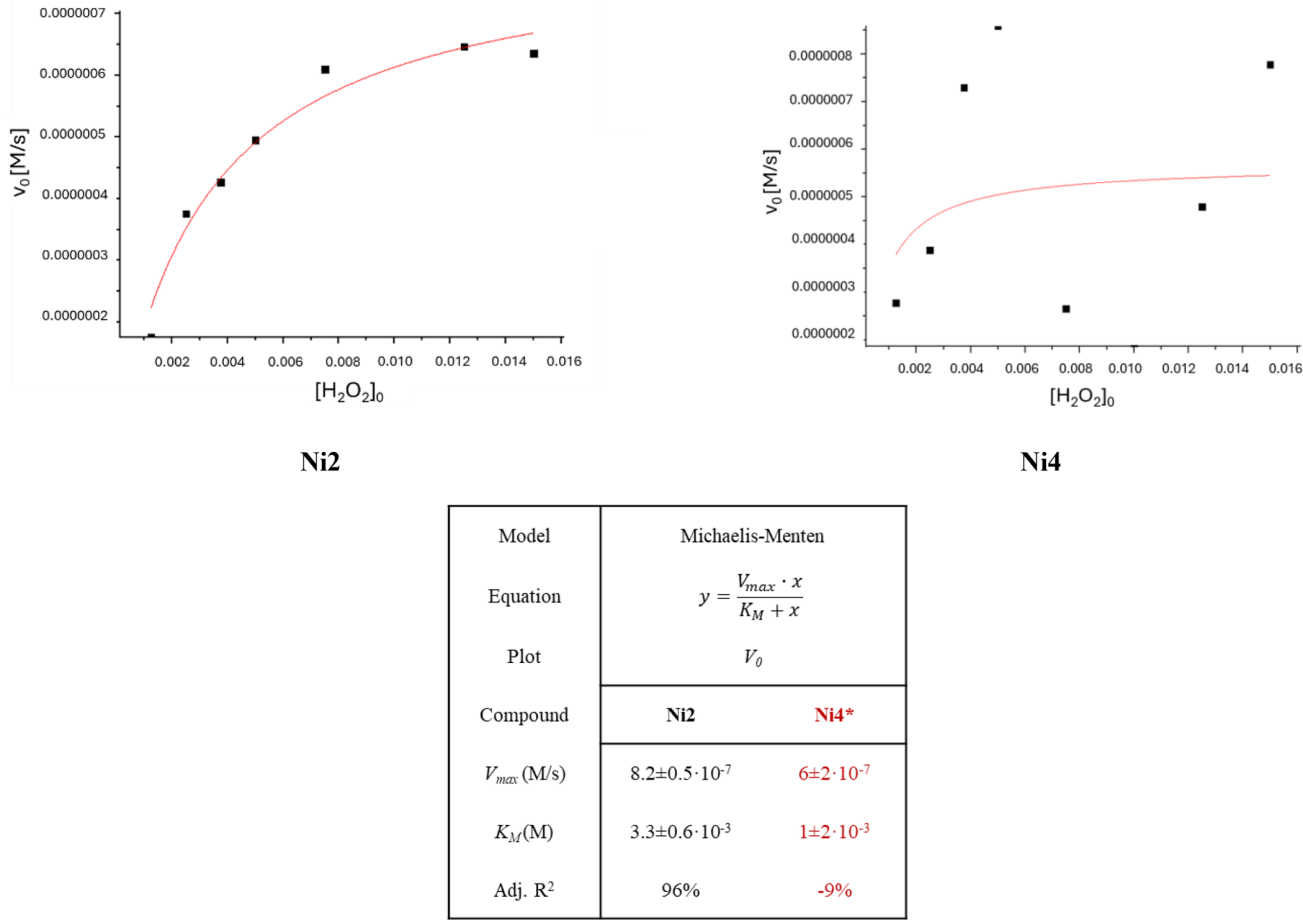
Kinetic Analysis of Consumption Capacity of **Ni2** and **Ni4** ^a^

a Michaelis–Menten plot of the initial velocities obtained through Clark-type oximeter assays. All measurements were carried out in PBS buffer (pH 7.4, [PBS] = 10 mM, [NaCl] = 137 mM, [KCl] = 2.7 mM) with 5% DMSO in 50 mL centrifuge tubes. H_2_O_2_ stock solution at 880 mM in double-distilled water, **Ni2** stock solution in DMSO at 1.25 mM, and **Ni4** stock solution at 1 mM were prepared. Considering a total volume of 10 mL, additions of complexes solutions were tuned to have a total 50 μM concentration, whereas the H_2_O_2_ final concentrations were 1.25, 2.5, 3.75, 5, 7.5, 12.5, and 15 mM. After having purged the PBS solution with dinitrogen gas to reduce dissolved oxygen concentration under 1.5 mg/L (47 μM), complexes and H_2_O_2_ were added and O_2_ was measured over time for 15–20 min. Below, the data obtained from the two plots and, in red, **Ni4** results highlighted.

We found that the two complexes work differently: On one side, **Ni4** consumed H_2_O_2_ catalytically, but without a direct proportionality related to the Michaelis–Menten equation; on the other side, **Ni2** exerted an efficient Michaelis–Menten catalytic mechanism, which is comparable to CAT-like synzymes from literature (see Table 2), with a *k*_cat_/*K*_M_ of 10 ± 2 M^–1^ s^–1^ mostly due to a high affinity between substrate (H_2_O_2_) and catalyst (**Ni2**), being *K*_M_ 3.3 ± 0.6 × 10^–3^ M. These data seemed not only coherent with the results of the Amplex Red Kit experiment in general but even justified the higher efficiency of **Ni2** in consuming H_2_O_2_.

**Table 2:** **Ni2** Compared to Catalase and Catalase Mimics in Literature

	*K*_M_ (M)	*k*_cat_ (s^–1^)	*k*_cat_/*K*_M_(M^–1^ s^–1^)	ref.
**Ni2**	3.3 ± 0.6 × 10^–3^	3.3 ± 0.2 × 10^–2^	10 ± 2	this work
**(CATm2)Cu**_**2**_	2.9 × 10^–2^	1.1 × 10^–1^	3.9	Ben Hadj Hammouda et al.^52^
**1**	6.6 × 10^–3^	10.5	1600	Palopoli et al.^55^
2 (Fe)	4.2 × 10^–2^	2.2 × 10^3^	5.2 × 10^4^	Karbalaei et al.^56^
**catalase**	8.3 × 10^–2^	2.6 × 10^5^	3.1 × 10^6^	Shank et al.^57^

We assessed the effect of the compounds on ROS balance on the side of enzymes involved in ROS regulation. **Ni2** treatment compared to the ligand was only able to slightly induce the expression of the HO-1 gene at 24 h. On the other side, **Ni4** treatment compared to the ligand was able to induce a significant increase in the gene expression of both HO-1 and SOD-2 for all of the times examined. Both complexes with their respective ligands did not induce any change in expression of the SOD-1 gene (Chart 16).

**Chart 16:**
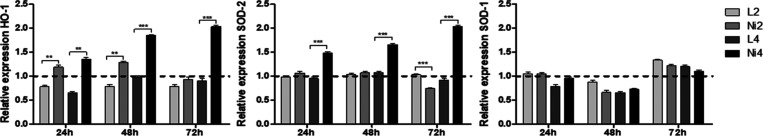
Effect of Ligands **L2** and **L4** and Complexes **Ni2** and **Ni4** on HO-1, SOD-2, and SOD-1 Gene Expression after 24, 48, and 72 h of Treatment^a^

a Values are mean ± SD of three separate experiments, each carried out in triplicate (dashed line = CTR). Statistical significance is indicated as follows: **p < 0.01, ***p < 0.001.

## Discussion

These new thiosemicarbazone Ni­(II) complexes were revealed to be almost isostructural as coordination systems, as they are all square planar and trans and even the coordination bond lengths are almost identical. Conversely, their different N4 substitutions caused important differences in cytotoxicity, electronic behavior, and biomolecular interactions. In particular, the alkyl-substituted **Ni2** and **Ni4** were revealed to be the best compromise as redox-balance disrupters and DNA-targeting twisters, explaining their enhanced cytotoxicity. Conversely, **Ni4**’s high selectivity is probably related to its strong affinity for albumin, contrarily to **Ni2**, but it is not possible to exclude a relation with the different effect on the expression of ROS-regulating enzymes as well.

The varying stability and reactivity toward ROS (verified preliminarily by electronic spectroscopy, see Charts 4 and 5 and Figures S35 and S36, and consequently *in vitro* and *in vial* oximetry, Charts 14 and 15) seemed to be coherent with the CV results (Chart 6). The DPPH assay and the UV–visible stability experiment toward the ^·^OH radical showed that DPPH reacted more with free ligands than with complexes and that in general complexes interacted more with the ^·^OH radical than with DPPH. We hypothesized that the DPPH radical had a higher affinity for the exposed sulfur moieties of the ligands, which can easily stabilize a radical cation, whereas Ni­(II) seems to be more prone to a two-electron oxidation (Chart 7). The ^·^OH radical oxidizes in a one-electron fashion as DPPH, but its smaller size may allow for a fast exchange of two OH molecules on the same Ni­(II) center, justifying the degradation reported in the electronic spectra. It is to be noted that **Ni3**, characterized by a bulky phenyl N4 substituent, displayed significantly lower activity than **Ni1** and **Ni2**, whereas **L1**–**3** all share comparable RSA% values. This hypothesis is also coherent with the fact that the degradation due to OH on **Ni2** and **Ni4** seems to take place in proximity of the metal, with also the charge transfer absorption bands being involved in the modifications. From a thermodynamical point of view, the possibility of an oxidation of the nickel complexes by DPPH and OH is corroborated by the comparison of the estimated *E*°_Ni(II)/Ni(III)_ and *E*°_Ni(III)/Ni(IV)_ values and literature values (*E*°_DPPH/DPPHH_: 0.35 V against the Ag/AgCl reference electrode; *E*°_OH/H2O_: 2.32 V against the standard hydrogen electrode),^58^ displaying favorable oxidations by these radical species.

The stability of the complexes toward O_2_^–^ and H_2_O_2_ is not necessarily related to a complete absence of reactivity. As an example, the Amplex Red and the Clark-type assays (see Charts 14 and 15) revealed that both **Ni2** and **Ni4** displayed an interesting H_2_O_2_ consumption capacity even if the UV–visible preliminary experiments showed absence of changes in the spectra. This seemed peculiarly coherent with the CV results (Chart 6), which revealed that these two complexes displayed the most reversible electron exchanges among all the compounds. On the other side, if we accepted a CAT-like activity for **Ni2** and a similar one for **Ni4**, we discarded a superoxide dismutase (SOD)-like activity for the same compounds, since it would result in a H_2_O_2_ production that seemed opposed to the Amplex Red assay outcome. A **Ni2** CAT-like behavior is hypothesized to happen through a first oxidation step, followed by a subsequent reduction of the Ni­(II) center. **Ni2** double oxidation by H_2_O_2_ is sustained by comparison between the reduction potentials to literature (*E*°_H2O2/H2O_: 1.35 V against standard hydrogen electrode),^59^ which seems more reasonable than a reduction–oxidation pathway. Overall, **Ni2** and **Ni4** alkylic substitution may have had a role in electron-enriching the Ni­(II) center to enable it to display the redox properties needed to disrupt the inner ROS balance of the cancer cells.

This is coherent with the computational data as well: All of the compounds seem to need a two-electron oxidation to be activated, in order to achieve a sufficient lowering of the HOMO–LUMO gap to start a catalytic-like activity (Table 1), but the exclusivity of **Ni2** and **Ni4** is related to their HOMO lower stability as neutral species (Table 1, bold values on the left). Therefore, even if all of the complexes share their overall reactivity, the alkylic substitution of **Ni2** and **Ni4** is essential for their efficacy in ROS balance disruption.

Conversely, **Ni2** and **Ni4** displayed very different effects on the regulation of ROS-related enzymes, specifically heme oxygenase (HO-1), cytoplasmic superoxide dismutase (SOD-1), and mitochondrial superoxide dismutase (SOD-2) (Chart 16). In particular, even if **Ni2** seemed to be the most effective on the ROS side due to the chemical and *in vitro* test, its influence on gene expression is slight to negligible. On the other hand, **Ni4** had a strong regulatory role on the expression of HO-1 and SOD-2, revealing one possible reason for its selectivity (compared to the nonselective **Ni2**) and informing about its partial localization in mitochondria (in addition to the nucleus).

The most probable explanation for **Ni4** selectivity is related to albumin. Albumin is a target of antitumor drug delivery due to the enhanced absorption of such proteins by cancer cells, improving both cytotoxicity and selectivity. Once confirmed that the metal ion was not cleaved by albumin (Figure S40), we found that the best candidates were, in order, **Ni4**, **Ni3**, and **Ni2** (Chart 9). Considering that the cytotoxic compounds, **Ni2** and **Ni4**, displayed very different affinities for albumin, and since **Ni3** is well bound by albumin but revealed a negligible cytotoxicity (Chart 7), we support that albumin as a delivery system is optimal to enhance selectivity but does not impact cytotoxicity.

We found therefore that **Ni2** and **Ni4** feature an unprecedented mix of modes of action to target efficiently and selectively cancer cells, and **Ni4** feasibility to be delivered by albumin and to hit the regulation of ROS-related enzymes renders it more than 8-fold selective toward cancer cells.

## Conclusions

Four new thiosemicarbazone Ni­(II) complexes were synthesized and characterized. Their XRD structures were revealed to be almost isostructural as coordination systems, as they are all square planar and trans and even the coordination bond lengths are almost identical. Conversely, their different N4 substitutions caused important differences in cytotoxicity, electronic behavior, and biomolecular interactions.

We tested these complexes on four cancer cell lines and on a healthy cell line, to scan both the effectivity and the selectivity of the compounds, finding that **Ni1** and **Ni3** are negligible, **Ni2** is toxic but not selective, and **Ni4** is both very effective and selective, with an IC_50_ of 5 μM with A549 and an SI of 8.2 comparing A549 and HuDe.

These results were accompanied by a deep insight into the modes of action of the complexes, finding that they are multiple and the main roles are ascribable to albumin delivery, DNA interaction, and ROS equilibrium disruption.

DNA interaction showed that the compounds potentially bind DNA on the minor groove, but only **Ni2** and **Ni4**, the ones which are cytotoxic, impact DNA helicity. This is a probable way in which the compounds cause apoptosis. The modification observed on CT-DNA is analogous to the one observed on the DNA extracted from treated cells, supporting that the compounds reach the DNA in the nucleus and have the same effect seen *a priori*.

The redox activity of the complexes and the role of the Ni­(II) center in interacting with ROS were shown to be crucial as well. The analysis of **Ni2** and **Ni4** with CV, UV, and Clark-type oximetry and, parallelly, *in vitro*, revealed that their redox activity causes oxidative stress on cells, explaining their efficacy. On the other hand, **Ni4** selectivity can be partially related to its capability in regulating ROS-regulating enzymes HO-1 and SOD-2.

**Ni4** exclusive selectivity is most probably due to its high affinity for albumin, which acts as delivery system toward cancer cells.

We found therefore that **Ni2** and **Ni4** feature an unprecedented mix of modes of action to target efficiently and selectively cancer cells, and **Ni4** feasibility to be delivered by albumin and to hit the regulation of ROS-related enzymes renders it more than 8-fold selective toward cancer cells.

## Experimental Section

### General

All common laboratory chemicals were purchased from commercial sources and used without further purification: 3,4-dimethoxybenzaldehyde, ≥99.0% (Janssen Chimica, Geel, Belgium); thiosemicarbazide, ≥99.9% (Fluka, Buchs, Switzerland); 4-methyl-3-thiosemicarbazide, 97% (Fluorochem, Hadfield, UK); 4-phenyl-3-thiosemicarbazide, 99% (Sigma-Aldrich, Burlington, Massachusetts, USA); 4-(2-morpholinoethyl)-3-thiosemicarbazide (Fluorochem, Hadfield, UK); nickel­(II) acetate, 99% (Carlo Erba, Milano); ethidium bromide, 10 mg/mL solution in water (Sigma-Aldrich, Burlington, Massachusetts, USA); lyophilized calf thymus DNA (SERVA Electrophoresis GmbH, Heidelberg, Germany); bovine serum albumin, 98% (VWR, Phillipsburg, New Jersey, USA); hydrogen peroxide 30% in water (Sigma-Aldrich, Burlington, Massachusetts, USA); 20 μm Ø microfilters (Eppendorf, Hamburg, Germany); phosphate-buffered solution tablets (VWR, Phillipsburg, New Jersey, USA).

NMR spectra were recorded on a Bruker Anova spectrometer at 400 MHz (Billerica, Massachusetts, USA) or on a JEOL ECZ600R at 600 MHz (Akishima, Tokyo, Japan), with chemical shifts reported in δ units (ppm). The NMR spectra were referenced relative to the residual solvent peaks. The solvent used for the spectral acquisitions was DMSO-d6. FT-IR measurements were recorded on a Nicolet 5PC FT-IR spectrometer (Rodano, Milan, Italy) in the range of 4000–400 cm^–1^ and equipped with an ATR accessory. Elemental analyses were performed using the Thermo Fisher Scientific FlashSmart CHNS Elemental Analyzer (Rodano, Milan, Italy). ESI-MS data were collected on a Waters Acquity Ultraperformance ESI-MS spectrometer with a Single Quadrupole Detector (Sesto San Giovanni, Milan, Italy). Fluorescence spectra were collected with an FLS1000 Edinburgh Instruments fluorometer (Edinburgh, Scotland, UK) equipped with a 450 W xenon lamp as the excitation source, using standard 1 cm × 1 cm 3 mL quartz cuvettes. To minimize inner-filter effects, samples with optical densities <0.1 were analyzed.

The software applications used for data interpretation are GraphPad Prism 8.0.2 (263) (GraphPad Software Inc., San Diego, California, United States), Origin 2019 64bit (OriginLab Corporation, Northampton, Massachusetts, United States), SkanIt RE 7.1 (Thermo Fisher Scientific, Inc., Waltham, Massachusetts, United States), and FlowJo 10.9 Software (Tree Star Inc., Ashland, Oregon, United States). Biological results are expressed as means ± SD of at least three independent experiments. Statistical analysis on biological results was performed by (SPSS Inc./IBM, Chicago, Illinois, USA), using one-way ANOVA with Dunnett’s or Tukey’s post hoc tests. *p*-values <0.05 (two-sided) were considered as statistically significant.

### Synthesis and Characterization

#### Ligands

We followed the same synthetic protocol for all the ligands.^31,34,35^ The thiosemicarbazide (1.25 mmol; unsubstituted, *N*-methyl, *N*-phenyl-, *N*-(2-morpholinoethyl)-) was mixed in a 1:1 stoichiometry with 3,4-dimethoxybenzaldehyde (1.25 mmol) in ethanol (15 mL), and a catalytic amount (few drops) of acetic acid was added. The mixture was refluxed overnight, and then the mixture was cooled to 4 °C to promote precipitation. The resulting solid was filtered, washed with diethyl ether, and dried at the vacuum line.

##### (*E*)-2-(3,4-Dimethoxybenzylidene)­hydrazine-1-carbothioamide (**L1**)

Yield: 86%. Appearance: white powder. Elemental analysis C_10_H_13_N_3_O_2_S calcd: C 50.19%, N 17.56%, H 5.48%, S 13.40% exp: C 49.84%, N 16.84%, H 5.56%, S 13.07%. ^1^H NMR (400 MHz, DMSO-*d*_6_): [ppm] 11.27 (s, 1H, C=N–NH); 8.09 (s, 1H, NH_2_); 7.97 (s, 1H, CH=N); 7.96 (s, 1H, NH_2_); 7.50 (d, 1H, CH_arom_); 7.14 (dd, 1H, CH_arom_); 6.95 (d, 1H, CH_arom_); 3.82 (s, 3H, CH_3_); 3.79 (s, 3H, CH_3_). ^13^C NMR (101 MHz, DMSO-*d*_6_): [ppm] 177.7, 150.7, 149.2, 142.6, 127.0, 122.1, 111.4, 108.7, 55.6. IR (ATR, cm^–1^): 3350, 3260 w (N–H), 3116 m (C–H_arom._), 2960 m (C–H_aliph._), 1617 m (C=N), 1096, 853 m (C=S). ESI-MS (*m*/*z*, %): 240 ([M + H]^+^, 60).

##### (*E*)-2-(3,4-Dimethoxybenzylidene)-*N*-methylhydrazine-1-carbothioamide (**L2**)

Yield: 83%. Appearance: white powder. Elemental analysis C_11_H_15_N_3_O_2_S calcd: C 52.16%, N 16.59%, H 5.97%, S 12.66% exp: C 51.74%, N 15.91%, H 6.00%, S 12.35%. ^1^H NMR (400 MHz, DMSO-d6): [ppm] 11.33 (s, 1H, C=N–NH); 8.38 (m, 1H, NH­(CH_3_)); 7.98 (s, 1H, CH=N); 7.45 (d, 1H, CH_arom_); 7.21 (dd, 1H, CH_arom_); 6.98 (d, 1H, CH_arom_); 3.84 (s, 3H, CH_3_); 3.80 (s, 3H, CH_3_); 3.04 (d, 3H, NH­(CH_3_)). ^13^C NMR (101 MHz, DMSO-d6): [ppm] 177.6, 150.6, 149.1, 142.0, 127.0, 121.8, 111.5, 109.1, 56.0, 30.9. IR (ATR, cm^–1^): 3352, 3149 m (N–H), 2990 m (C–H_arom_.), 2962 w (C–H_aliph_.), 1602 m (C=N), 1084, 862 m (C=S). ESI-MS (*m*/*z*, %): 254 ([M + H]^+^, 79).

##### (*E*)-2-(3,4-Dimethoxybenzylidene)-*N*-phenylhydrazine-1-carbothioamide (**L3**)

Yield: 92%. Appearance: white-yellow powder. Elemental analysis calcd: C 60.93%, N 13.32%, H 5.43%, S 10.17% exp: C 60.44%, N 12.79%, H 5.47%, S 10.06%. ^1^H NMR (400 MHz, DMSO-*d*_6_): [ppm] 11.73 (s, 1H, C=N–NH); 10.02 (s, 1H, S=C–NH-ph); 8.09 (s, 1H, CH=N); 7.56 (m, 3H, CH_arom.ald._); 7.37 (t, 2H, CH_arom_); 7.27 (dd, 1H, CH_arom.ald_); 7.21 (t, 1H, CH_arom_); 6.99 (d, 1H, CH_arom.ald_); 3.83 (s, 3H, CH_3_); 3.80 (s, 3H, CH_3_). ^13^C NMR (101 MHz, DMSO-*d*_6_): [ppm] 175.8, 161.3, 159.3, 151.0, 149.2, 143.3, 139.3, 128.2, 126.7, 126.0, 125.4, 122.4, 111.7, 109.4, 55.6. IR (ATR, cm^–1^): 3336, 3311 w (N–H), 3142, 2993 m (C–H_arom._), 2953 w (C–H_aliph._), 1598 m (C=N), 1072, 862 m (C=S). ESI-MS (*m*/*z*, %): 316 ([M + H]^+^, 82).

##### (*E*)-2-(3,4-Dimethoxybenzylidene)-*N*-(2-morpholinoethyl)­hydrazine-1-carbothioamide (**L4**)

Yield: 92%. Appearance: at first, a yellow oil. After several trituration passages with diethyl ether, a white-yellow powder was obtained. Elemental analysis calcd: C 54.53%, N 15.90%, H 6.86%, S 9.00% exp: C 54.59%, N 14.96%, H 6.83%, S 9.03%. ^1^H NMR (400 MHz, DMSO-*d*_6_): [ppm] 11.42 (s, 1H, C=N–NH); 8.31 (9.98 (s, 1H, S=C–NH–R); 8.00 (s, 1H, CH=N); 7.37 (m, 3H, CH_arom._); 7.22 (dd, 1H, CH_arom._); 6.99 (d, 1H, CH_arom._); 3.83 (s, 3H, CH_3_); 3.80 (s, 3H, CH_3_); 3.68 (m, 2H, S=C–NH–C**H**_**2**_−); 3.58 (t, 4H, −CH_2_–O–CH_2_); 2.54 (m, 2H, S=C–NH–CH_2_–C**H**_**2**_–N); 2.44 (m, 4H, −CH_2_–N–CH_2_). ^13^C NMR (101 MHz, DMSO-_d6_): [ppm] 176.7, 150.7, 149.1, 142.5, 126.8, 121.7, 111.5, 109.1, 66.4, 56.6, 55.8, 53.2. IR (ATR, cm^–1^): 3341 w, 3229 b (N–H), 2958, 2908, 2887, 2860, 2837, 2814 m (C–H), 1601 m (C=N), 1068, 847 m (C=S). ESI-MS (*m*/*z*, %): 353 ([M + H]^+^, 100).

#### Complexes

The same synthetic procedure was performed for all the complexes. We added the ligand (0.37 mmol) to refluxing ethanol (10 mL) and stirred it for 1–2 h. Afterward, a solution of Ni­(OAc)_2_·4H_2_O (0.19 mmol) was added to the main mixture, which was cooled to room temperature and left stirring overnight. The formation of the Ni­(II) complexes was accompanied by an intense color shift to yellow/light brown. The Ni­(II) complexes were precipitated, isolated, and eventually washed with diethyl ether.

##### Ni1

Yield: 79%. Appearance: light-brown powder. Elemental analysis: calcd: C 44.88%, N 15.70%, H 4.52%, S 11.98%; exp.: C 44.92%, N 15.60%, H 4.62%, S 11.98%. ^1^H NMR (400 MHz, DMSO-*d*_6_): [ppm] 8.74 (s, 1H, NH_2_); 7.26 (s, 1H, CH=N); 7.04 (s, 1H, NH_2_); 7.00 (d, 1H, CH_arom_); 6.59 (s, 2H, CH_arom_); 4.03 (s, 3H, CH_3_); 3.83 (s, 3H, CH_3_). IR (ATR, cm^–1^): 3426, 3289 w (N–H), 3177, 3139, 2952, 2911, 2832 w (C–H), 1626 m (C=N), 1019 s, 794 m (C=S). ESI-MS (*m*/*z*, %): 535 ([M + H]^+^, 100).

##### Ni2

Yield: quant. Appearance: light-brown powder. Elemental analysis: calcd: C 46.91%, N 14.92%, H 5.01%, S 11.38%; exp.: C 46.38%, N 14.73%, H 5.07%, S 11.23%. ^1^H NMR (400 MHz, DMSO-*d*_6_): [ppm] 8.74 (s, 1H, N**H**(CH_3_)); 7.40 (s, 1H, CH=N); 7.16 (s, 1H, CH_arom_); 7.01 (m, 2H, CH_arom_); 3.97 (s, 3H, CH_3_); 3.84 (s, 3H, CH_3_); 2.64 (d, 3H, NH­(C**H**_**3**_)). IR (ATR, cm^–1^): 3220 w (N–H), 3087, 2958, 2934, 2899, 2834 w (C–H), 1601 m (C=N), 1015, 850 m (C=S). ESI-MS (*m*/*z*, %): 563 ([M + H]^+^, 100).

##### Ni3

Yield: 40%. Appearance: light-brown solid. Elemental analysis: calcd: C 51.83%, N 11.33%, H 5.17%, S 8.65%; exp.: C 51.92%, N 11.56%, H 5.06%, S 8.49. ^1^H NMR (400 MHz, DMSO-*d*_6_): [ppm] 9.66 (s, 1H, S=C–NH–Ph); 7.97 (s, 1H, CH=N); 7.64 (m, 2H, CH_arom._); 7.58 (m, 2H, CH_arom._); 7.29 (t, 2H, CH_arom_); 7.00 (t, 2H, CH_arom_); 3.81 (s, 3H, CH_3_); 3.46 (s, 3H, CH_3_). IR (ATR, cm^–1^): 3294 m (N–H), 3117, 3061, 3032, 3005, 2958, 2926, 2834 w (C–H), 1592 m (C=N), 1021, 833 m (C=S). ESI-MS (*m*/*z*, %): 688 ([M + H]^+^, 36).

##### Ni4

Yield: 58%. Appearance: light-brown powder. Elemental analysis calcd: C 50.47%, N 14.71%, H 6.09%, S 8.42% exp: C 50.23%, N 14.55%, H 6.11%, S 8.29%. ^1^H NMR (400 MHz, DMSO-*d*_6_): [ppm] 8.12 (s, 1H, S=C–NH–R); 7.48 (d, 1H, CH_arom._); 7.34 (m, 1H, CH_arom._); 7.31 (s, 1H, CH=N); 6.97 (d, 1H, CH_arom._); 3.80 (s, 3H, CH_3_); 3.76 (s, 3H, CH_3_); 3.56 (m, 6H, S=C–NH–C**H**_**2**_– and −CH_2_–O–CH_2_); 2.47 (m, 2H, S=C–NH–CH_2_–C**H**_**2**_–N); 2.37 (m, 4H, −CH_2_–N–CH_2_). IR (ATR, cm^–1^): 3338 w, (N–H), 3123, 3002, 2973, 2940, 2864, 2840, 2802, 2767 m (C–H), 1592 m (C=N), 1036, 815 m (C=S). ESI-MS (*m*/*z*, %): 762 ([M + H]^+^, 92).

#### X-ray Diffraction Characterization

Crystals suitable for X-ray diffraction were obtained for **L4**, **Ni1**, **Ni2**, **Ni3**, and **Ni4** by slow evaporation of EtOH solutions. Single-crystal X-ray diffraction analyses were carried out with a Bruker D8 VENTURE diffractometer equipped with a kappa goniometer and an Oxford cryosystem. Microfocused Mo Kα radiation (λ = 0.71073 Å) and Cu Kα radiation (λ = 1.54178 Å) were used as the X-ray source, and Lorentz polarization and absorption correction were applied through the SADABS^60^ procedure. The phase problem was solved by direct methods, and the structures were refined by full-matrix least squares on all F2 using SHELXL,^61,62^ as implemented in the OLEX2 ^63^ suite of programs. The structure drawings were obtained using ORTEPIII^64^ and Mercury (Table 3).^65^

**Table 3:** X-ray Crystallographic Data

	L4	Ni1	Ni2	Ni3	Ni4
**empirical formula**	C16 H24 N4 O3 S	C20 H24 N6 Ni O4 S2	C22 H28 N6 Ni O4 S2	C32 H32 N6 Ni O4 S2	C32 H45 N8 Ni O6 S2
**formula weight**	352.45	535.28	563.33	687.46	760.57
**temperature (K)**	200.0	200.0	200.0	200.0	200.0
**diffractometer/detector**	Bruker D8 Venture/Photon II area detector	Bruker D8 Venture/Photon II area detector	Bruker D8 Venture/Photon II area detector	Bruker D8 Venture/Photon II area detector	Bruker D8 Venture/Photon II area detector
**radiation**	Mo Kα (λ = 0.71073 Å)	Cu Kα (λ = 1.54178 Å)	Mo Kα (λ = 0.71073 Å)	Mo Kα (λ = 0.71073 Å)	Mo Kα (λ = 0.71073 Å)
**crystal system**	monoclinic	monoclinic	orthorhombic	monoclinic	orthorhombic
**space group**	*C*2/*c*	P2_1_/*n*	*Pbca*	*P*2_1_/*c*	*Pca*2_1_
***a* (Å)**	26.5449(13)	8.3812(6)	14.1018(6)	11.0714(4)	25.5921(17)
***b* (Å)**	9.7158(5)	10.9551(9)	16.7127(6)	9.4041(3)	4.9956(3)
***c* (Å)**	18.8586(16)	12.7516(9)	21.4838(9)	16.5062(6)	27.1000(17)
**α (°)**	90	90	90	90	90
**β (°)**	129.9730(10)	106.607(5)	90	109.5760(10)	90
**γ (°)**	90	90	90	90	90
**volume (Å** ^3^)	3727.3(4)	1121.97(15)	5063.3(4)	1619.23(10)	3464.7(4)
***Z***	4	2	8	2	4
**ρcalc (g·cm** **^–3^)**	1.256	1.584	1.478	1.410	1.458
**μ (mm** **^–1^)**	0.195	3.345	0.972	0.774	0.737
***F*(000)**	1504	556	2352	716	1604
Θ range for data collection (°)	2.00–25.68	5.42–66.59	1.90–26.38	1.95–25.68	2.19–26.46
**index ranges**	–32 < *h* < 32	–7 < *h* < 9	–17 < *h* < 17	–13 < *h* < 13	–32 < *h* < 32
	–11 < *k* < 11	–12 < *k* < 10	–20 < *k* < 20	–11 < *k* < 11	–6 < *k* < 6
	–22 < *l* < 22	–14 < *l* < 15	–26 < *l* < 26	–20 < *l* < 20	–33 < *l* < 33
**reflections collected**	76,937	12,164	70,341	61,484	93,357
**unique reflections**	3540	1955	5176	3069	7121
**parameters**	291	153	322	207	447
**goodness-of-fit on *F*** ^**2**^	1.044	1.076	1.036	1.050	1.034
**final *R* indexes**	*R* = 0.043	*R* = 0.099	*R* = 0.034	*R* = 0.018	*R* = 0.065
**[*I* ≥2σ(*I*)]**	*wR*2 = 0.114	*wR*2 = 0.238	*wR*2 = 0.080	*wR*2 = 0.074	*wR*2 = 0.156
**CCDC number**	2430125	2430126	2430128	2430127	2430129

#### Electronic Spectroscopy

UV/visible spectra were obtained using a Thermo Fisher Scientific Evolution 260 Bio Spectrophotometer (Rodano, Milan, Italy) and a PerkinElmer Lambda 465 (Milano, Italy), utilizing quartz cuvettes with a 1 cm path length. Electronic spectra of the complexes were collected at 7 μM in DMSO. Stability in aqueous medium (5% DMSO in PBS buffer at pH 7.4, with [PBS] = 10 mM, [NaCl] = 137 mM, [KCl] = 2.7 mM) was monitored in a 24 h interval: The compounds were incubated at 37 °C in a standardized chamber.

The DPPH^36^ scavenging activity was studied using a colorimetric method. Solutions of 200 μM of the complexes and ligands were prepared in DMSO. A 100 μM solution of DPPH was also prepared in MeOH. Different ratios (1 to 2, 1, and 0.5) of DPPH and each complex were obtained by mixing 500 μL of a MeOH 100 μM solution of DPPH in methanol and 500 μL of DMSO solutions of the compounds at different ratios and incubated for 30 min at 37 °C in the dark. Absorbances of radical DPPH, molecular DPPH, compounds, and compounds/DPPH mixtures were read at 517 nm and used to calculate the radical scavenging activity (RSA%) using the corrected eq 2:

$$\mathrm{RSA}\%=\frac{\left(A_{\mathrm{sample}}-A_{\mathrm{DPPH}\cdot }\right)}{\left(A_{\mathrm{DPPH}-}-A_{\mathrm{DPPH}\cdot }\right)}\times 100$$ 2

where *A*_DPPH_· is the absorbance of radical DPPH, *A*_DPPH–_ is the absorbance of the monoreduced DPPH, and *A*_sample_ is the mixture absorbance. *A*_DPPH_– is obtained from a DPPH solution at the same concentration in MeOH quenched with an excess of sodium ascorbate: This correction allows for a more precise value, which does not underestimate the absorbance of the quenched DPPH.^37^

Superoxide anion assay was performed in dry DMSO. Stock solutions of the ligands (10 mM), the complexes (5 mM), and KO_2_ (259 μM) were prepared in dry DMSO. The superoxide concentration was evaluated using Lambert–Beer Law (ε = 2686 cm^–1^ mol ^–1^ L at λ = 280 nm) and diluted to 14 μM, while the solutions of the compounds were diluted to 7 μmol/L. UV–visible spectra were obtained after incremental additions (0, 10, 20, 30, and 40 μL) of KO_2_ to the ligands/complexes solution (600 μL).

Hydroxyl radicals are generated *in situ* by a Fenton reaction. Briefly, in a 45.4 μM solution of FeSO_4_ in water is added a DMSO solution of target compound at 9.1 μM in the final mixture. To this solution, H_2_O_2_ was added to achieve a final concentration of 454 μM. The UV–visible spectra were recorded every 40 s for 10 min.

H_2_O_2_ UV–visible stability assay was performed in distilled water. Solutions of the complex or ligand at a concentration of 10 μM were prepared in DMSO, while H_2_O_2_ was diluted in distilled water to obtain a 1 M stock solution. UV–visible spectra were registered in the 250–750 nm range every 12 s for 5 min on 500 μL of the compound solution after the addition of 100 μL of H_2_O_2_ stock solution.

#### Cyclic Voltammetry

Cyclic voltammetry (CV) experiments were carried out using a μStat 8000 Multi Potentiostat/Galvanostat (Metrohm Italiana S.r.l., Origgio, Italy) on screen-printed electrodes featuring carbon working and counter electrodes and a silver pseudoreference electrode (SPCE DRP-C110, Metrohm Italiana S.r.l., Origgio, Italy). Data acquisition and elaboration were conducted using DropView 8400 software (version 3.78). The CV characterization of **Ni1**–**Ni4** complexes was conducted in acetonitrile (ACN) solutions with complex concentrations ranging from 0.1 to 1 mM, also containing 0.1 M tetrabutylammonium tetrafluoroborate as the supporting electrolyte. To achieve this, SPCEs were immersed in the solutions described above, and the resulting CVs were acquired within a potential window ranging from −2.5 to +1.5 V with a scan rate of 0.1 V/s. As for the potential of the pseudoreference electrode of the SPCEs, 1 mM ferrocene solution, prepared in acetonitrile containing 0.1 M tetrabutylammonium hexafluorophosphate, exhibited an *E*_1/2_ potential of +0.5 V with respect to the silver printed electrode. The voltametric characterization of complexes was carried out in acetonitrile solution, although complete solubility was not achieved, by analyzing the saturated solutions; in fact, lower concentrations were preferred over the use of alternative solvents because ACN provided a higher resolution of the CV peaks compared to DCM or DMF, which allowed for higher concentrations but resulted in reduced resolution of the voltammograms.

#### Computational Analysis

All calculations were performed with Gaussian 16.^66^ Geometries were optimized in the gas phase using the B3LYP/6-31+G­(**) level of theory.^67–71^ Frequency calculations (at 298.15 K) at the same level of theory were used to confirm the nature of all stationary points as minima and provided values for computing free energies.

#### Cytotoxicity Assays

Cell lines used for biological experiments were adenocarcinomic human alveolar basal epithelial cells A549, the human acute promyelocytic leukemia cell line HL60, the human colorectal adenocarcinoma cell line HT29, and the human epithelial mesothelioma cell line H2052 (ATCC, Rockville, Maryland, United States), using non-neoplastic human dermal fibroblast cell line HuDe (Istituto Zooprofilattico Sperimentale della Lombardia e dell’Emilia (IZSLE), Brescia, Italy) for selectivity analysis. Varioskan LUX (Thermo Fisher Scientific Inc., Waltham, Massachusetts, United States) was used to perform MTT assay, DNA quantification, thiobarbituric acid and reactive substance (TBARS) assay, hydrogen peroxide assay on the cell supernatant using Amplex Red Hydrogen Peroxide/Peroxidase Assay Kit (Invitrogen; Thermo Fisher Scientific, Inc., Waltham, Massachusetts, United States), and the enzymatic activity of the caspase-3 using Caspase-Glo 3/7 assay (Promega Corporation, Madison, Wisconsin, USA), according to the manufacturer’s instructions. All cells were cultured in RPMI 1640 supplemented with 10% fetal bovine serum, penicillin (100 U/mL), streptomycin (100 μg/mL), and l-glutamine (2 mM). Adherent cells were grown as subconfluent monolayers. Flasks and plates were maintained at 37 °C and 5% CO_2_ in a humid atmosphere.

The cells, in the exponential phase, were cultured on 96-well plates at a density of 1 × 10^5^ cells/mL and incubated for 24 h before the treatment with 50 or 100 μM concentrations of ligands or complexes, reconstituted in DMSO, and diluted in RPMI 1640 medium. The cell proliferation was examined by 3-(4,5-dimethylthiazole)-2,5-diphenyltetrazoliumbromide (MTT) assay. After this screening, IC_50_ (after treatment of 24 and 48 h) was calculated for the compounds that had been shown to inhibit cell proliferation. In this case, the MTT assay was performed using a range of concentrations (working range 0.1–100 μM). At the fixed time points, 10 μL of MTT reagent (5 mg/mL) was added into each well prior to incubation at 37 °C for 3 h. The reaction was terminated by the addition of 100 μL of solubilization solution and the absorbance was recorded using a Varioskan plate reader at a wavelength of 550 nm. The number of viable cells was determined using a calibration curve, consisting of a decreasing number of cells and confirmed by counting viable cells in a hemocytometer (trypan blue exclusion). All measurements were performed at least in triplicate. To evaluate the effect of the treatment of the various cell lines with the ligands and the related Ni complexes, a screening was performed using the various compounds at 100 μM for 24 and 48 h. Subsequently, the compounds that showed an inhibition of proliferation were used at different concentrations (0.1–100 μM) and half maximal inhibitory concentration (IC_50_) were calculated at different times (24 and 48 h) for those compounds.

Ligands, **Ni1**, and **Ni3** did not inhibit the proliferation of the four cell lines studied up to a concentration of 100 μM, while treatment with **Ni2** and **Ni4** complexes significantly inhibited cell growth and affected cell viability in both concentration- and time-dependent ways.

#### Apoptosis

After exposure to the different compounds at the IC_50_ concentration, apoptosis was evaluated using the Annexin V/FITC kit assay (Invitrogen; Thermo Fisher Scientific, Inc., Waltham, Massachusetts, U.S.A.). Briefly, cells exposed to different compounds for 24, 48, and 72 h were washed in PBS, incubated with Annexin V-FITC and propidium iodide (PI) at room temperature for 15 min in the dark and analyzed using CytoFLEX (Beckman Coulter, Brea, California, USA). The data analysis was performed using FlowJo software (Ashland, Oregon, USA). Apoptosis was calculated as the percentage of early and late apoptotic cells.

The enzymatic activity of the caspase-3 was assayed using Caspase-Glo 3/7 assay, according to the manufacturer’s instructions. A549 cells were cultured in a 96-well culture plate (5000 cells/well) and exposed to **L2**, **Ni2**, **L4**, and **Ni4** at IC_50_ concentration for 24, 48, and 72 h. A549 cells were incubated with 100 μL of Caspase-Glo 3/7 reagent at 37 °C for 30 min. A Varioskan plate reader was used to measure the luminescence. Three independent experiments were performed, and each sample was measured in three replicates. The caspase-3 activity was calculated as a percentage normalized to control cells.

#### Interactions with Albumin

##### *Galleria mellonella* Larval Model

*Galleria mellonella* survival test is an affordable, easy, and reliable methodology for evaluating the in vivo toxicity of newly synthesized drug candidates.^46,72,73^ In this study, the larvae were injected with varying concentrations of the compounds and incubated at 37 °C for a week. *G. mellonella* larvae in the 400–500 mg range were divided into groups of 19 each and placed in large Petri plates recorded over a 6-day period of incubation at 37.4 °C postinjection of 10 μL of concentrated solutions (1.1, 0.57, and 0.28 mM) of the compounds in distilled water (10% DMSO), diluted in larva to the reported concentrations. The larvae were incubated at 37 °C and monitored daily for a week for survival. *G. mellonella* larvae were purchased from Fishing & Adventure S.r.l. (Parma, Italy).

##### Albumin Affinity: Circular Dichroism

Circular dichroism is a spectroscopic technique that allows for the detection of modifications in the chirality of biomolecular systems, such as proteins or DNA. Bovine serum albumin (BSA) exhibits Cotton effects in the 300–800 nm range when chelating Ni­(II) or Cu­(II), whereas it is silent in this interval as a free ligand. Circular dichroism spectroscopy was performed using a 3 mL quartz cuvette with a 1 cm path length. BSA was dissolved in a pH 7.4 solution (double-distilled water adjusted to pH 7.4 using NaOH 2 M) to a 12 μM concentration. Stock solutions of complexes **Ni1**–**4** and nickel acetate were prepared in DMSO at 600 μM. 35 μL of the stock solutions was added to the BSA solution: In the mixed solutions [albumin]/[compound] = 1.7, with the compounds diluted to 7 μM. Circular dichroism spectra were recorded after having mixed BSA and compounds.^74^ Circular dichroism measurements were performed using a J-1500 spectrophotometer (JASCO Corporation, Tokyo, Japan).

##### Albumin Affinity: Fluorescence Titrations

Albumin affinity fluorescence titrations were performed using a 3 mL quartz cuvette with a 1 cm path length. BSA was dissolved in a pH 7.4 buffered solution ([HEPES]: 50 mM; [NaCl]: 0.1 M; adjusted to pH: 7.4 using NaOH 2 M) to a 2.5 μM concentration. Stock solutions of compounds were prepared in DMSO at 60 μM. First, 2.5 mL of BSA solutions was analyzed. Afterward, more than 10 aliquots of 20 μL each were added to the albumin solution. Fluorescence spectra were recorded at λ_ex_ = 280 nm (the intensity of the emission was thoroughly checked at λ_em_ = 345 nm). The Stern–Volmer eq 3 was employed to estimate the Stern–Volmer constant (*K*_sv_)*:*

$$\frac{F_{0}}{F}=1+K_{\mathrm{SV}}[Q]$$ 3

where “*F*_0_” and “*F*” are the emission intensities of native BSA and BSA after addition of quencher, respectively, and “*Q*” represents the target compound considered as quencher.^49^ In addition, the modified Stern–Volmer eq 4 ^75^ was used to estimate the number of binding sites “*n*” and the binding constant (*K*_b_):

$$\log\;\frac{F_{0}-F}{F}=n\log\;K_{b}+n\log\left(\frac{1}{\left[Q]-\frac{F_{0}-F}{F_{0}}[P\right]}\right)$$ 4

where [*Q*] and [*P*] are the total concentration of quencher and of protein at that point of the titration. Complex formation is proved by quenching constant (*k*_q_) values. The following equation is used for their calculation:

$$k_{q}=\frac{K_{\mathrm{SV}}}{\tau_{0}}$$ 5

where *k*_q_ is the bimolecular quenching rate constant, τ_0_ is the average lifetime of the fluorophore in the excited state that is for a biomacromolecule 10^–8^ s.^76,77^ All the experiments were performed with solutions containing a whole percentage in volume of DMSO less than 15%, to preserve albumin from denaturation. We approximately tested the minimum amount of DMSO causing albumin precipitation due to unfolding, which was found to be around 25–30% in buffered aqueous solution at room temperature. Initially, 2.5 mL of BSA solutions was analyzed, followed by the additions of over 10 aliquots of 20 μL each to the albumin solution. Once the titration profiles were obtained and corrected for the dilution factor, the maximum values were plotted to determine Stern–Volmer constant (*K*_SV_), binding constant (*K*_b_), and the number of occupied sites (*n*). Additionally, after obtaining *K*_SV_, we calculated *k*_q_ to assess the static nature of the fluorescence quenching of BSA.

#### Interactions with DNA

##### Circular Dichroism with CT-DNA

Circular dichroism (CD) spectroscopy was performed using a 3 mL quartz cuvette with a 1 cm path length. Lyophilized calf thymus DNA (CT-DNA) was dissolved in PBS buffer (pH 7.4, [PBS] = 10 mmol/L, [NaCl] = 137 mmol/L, [KCl] = 2.7 mmol/L) over a period of 2 days and subsequently microfiltered twice using at first 45 μm-diameter filters and then through 20 μm-diameter filters. Concentration and purity were determined via electronic spectroscopy: with *A*_260_ = 0.25 and *A*_260_/*A*_280_ = 2.0 (>1.8), we estimated a concentration of [bp] = 37.6 μM and confirmed the solution’s sufficient purity. Complexes were dissolved in DMSO at 30 μM. Each complex and CT-DNA were mixed—in 10:200 (1.9 mL CT-DNA + 100 μL complex solution) and 1:200 (1.9 mL CT-DNA + 10 μL complex solution +90 DMSO) ratios—and CD spectra were recorded in the 240–305 nm range.

##### Ethidium Bromide Displacement Titrations

Ethidium bromide displacement fluorescence titrations were performed using a 3 mL quartz cuvette with a 1 cm path length. Lyophilized calf thymus DNA was dissolved in a pH 7.4 buffered solution ([HEPES]: 50 mM; [NaCl]: 0.1 M; adjusted to pH 7.4 using 2 M NaOH) over a period of 2 days and subsequently microfiltered using 45 μm-diameter filters. Concentration and purity were determined via electronic spectroscopy: With *A*_260_ = 0.36 and *A*_260_/*A*_280_ = 2.9 (>1.8), we estimated a concentration of [bp] = 54.5 μM and confirmed the solution’s sufficient purity. A stock solution of ethidium bromide (EthBr) was prepared by diluting a 10 mg/mL solution to 6.5 mM in double-distilled water. Ligands, complexes, and nickel acetate stock solutions were prepared in DMSO at a concentration of 600 μM. Spectra were recorded by exciting EthBr at λ_ex_ = 285 nm, and emission intensity was thoroughly checked at λ_em_ = 605 nm. The first spectrum was taken at 2.5 mL of the CT-DNA solution with the addition of 1 μL of EthBr stock solution, resulting in [EthBr] = 2.6 μM. Subsequently, we added 20 μL of compound/nickel acetate solution directly inside the cuvette 12 times. The dilution effect was accounted for by multiplying the spectral intensities for the dilution factor, i.e., (*V*_0_ + *V*_additions_)/*V*_0_. The apparent constant of binding (*K*_app_, [M^–1^]) of the compounds was estimated using eq 3, which is a variant of the equation 2 reported in a work by Banerjee et al.:^78^

$$K_{\mathrm{app}}=\frac{1+K_{\mathrm{EthBr}}[\mathrm{EthBr}]}{{\mathrm{IC}}_{50}}$$ 6

where *K*_EthBr_ = 2.2 × 10^5^ M^–1^ is considered as the mean binding constant of ethidium bromide to DNA,^79^ [EthBr] is the molar concentration of ethidium bromide (2.6 μM), and IC_50_ is the molar concentration of the target compound to which *F*_0_/*F* = 2. The IC_50_ value was achieved for each compound plotting *F*_0_/*F* against [target compound] and fitting a quadratic model suitable for the calculation (with *B*_0_ constrained to 1). Once the model was obtained, the abscissa relative to *F*_0_/*F* = 2 was considered as IC_50_.

##### Circular Dichroism on DNA Extracted from Treated Cells

A549 was treated at the IC_50_ concentration with **Ni4** and **L4** (as negative control) for 48 h and washed in PBS, and genomic DNA was extracted using the Gentra Puregene according to the manufacturer’s protocol. DNA quantification was performed using Varioskan LUX. We performed circular dichroism on the DNA extracted from untreated cells, cells treated with **Ni4** at IC_50_ concentration, and cells treated with **L4** at the same concentration. After extraction, we obtained 100 μL of DNA solution in three batches. These samples were diluted to 300 μL to fit the 0.1 cm-path-length quartz cuvette volume using a pH 7.4 buffered solution ([HEPES]: 50 mM; [NaCl]: 0.1 M; adjusted to pH 7.4 using 2 M NaOH). The concentration of the solutions slightly varied between 0.15 and 0.21 mM. Therefore, θ [mdeg] intensity was normalized.

#### Reactive oxygen species pathway

##### *I* *n Vitro* Oxidative Stress

The formation of intracellular reactive oxygen species (ROS) was revealed using 2,7-dichlorodihydrofluorescein diacetate (DCFH-DA), a nonpolar and nonfluorescent compound that diffuses into the cytoplasm where intracellular esterases cleave the acetate group to yield polar, nonfluorescent 2,7-dichlorofluorescein (DCF), whereas its reaction with ROS forms a highly fluorescent two-electron oxidation product. The A549 cells were pretreated with 10 μM DCFH-DA in PBS at 37 °C for 30 min in the dark and then incubated with **L2**, **Ni2**, **L4**, and **Ni4** at the IC_50_ concentration before being harvested, washed with PBS, and analyzed by means of a CitoFlex flow cytometer. Hydrogen peroxide (10 μM) was used as a positive control. Lipid peroxidation was evaluated using the thiobarbituric acid reactive substances (TBARS) method as previously described:^80^ The condensation of malondialdehyde (MDA) derived from polyunsaturated fatty acids with two equivalents of thiobarbituric acid gives a fluorescent red derivative that can be quantified using a Varioskan fluorescence spectrophotometer (λ_exc_: 515 nm, λ_em_: 545 nm). The values of MDA were normalized to protein concentrations and expressed as percentages of the controls.

Intracellular ROS were assayed early (after 1 h) because of their instability. In comparison with controls, **Ni2** and **Ni4** induced intracellular ROS after 1 h: In particular, the treatment with **Ni2** induced an ROS production of about 40% compared to the untreated control and that with **Ni4** of about 30%.

A CitoFlex flow cytometer (Beckman Coulter, Brea, California, United States) was used to perform intracellular reactive oxygen species measurement using DCFH-DA, and the apoptosis assay using Annexin V/FITC Assay Kit (Invitrogen; Thermo Fisher Scientific, Inc., Waltham, Massachusetts, United States).

Oxidative stress was also evaluated by quantifying oxidative DNA damage using ultrahigh-performance liquid chromatography coupled with tandem mass spectrometry (UHPLC–MS/MS). Elevated intracellular ROS levels induce DNA damage; 2′-deoxyguanosine (dGuo) and its oxidized form 8-oxo-2′-deoxyguanosine (8-oxodGuo) were quantified as biomarkers of oxidative DNA damage. Cells were treated with the compounds, and DNA was extracted from cell pellets using the Puregene Genomic DNA Isolation Kit (Gentra) and enzymatically hydrolyzed to nucleosides. The resulting nucleoside mixture was analyzed by UHPLC–MS/MS using a triple quadrupole mass spectrometer (API 6500+, AB Sciex) equipped with a TurboIonSpray electrospray ionization source, operating in positive ion mode. Chromatographic separation was achieved on an Atlantis dC18 column (100 × 2.0 mm, 3 μm; Waters) using a gradient elution with 10 mM formic acid (pH 3.75) and methanol. Samples were acidified with 0.2 M formic acid and spiked with stable isotope-labeled internal standards prior to injection. MS/MS acquisition was performed in Selected Reaction Monitoring (SRM) mode. Oxidative DNA damage was expressed as the ratio of 8-oxodGuo to dGuo. The method was validated according to FDA guidelines for bioanalytical methods.

##### Catalase-Like Hydrogen Peroxide Consumption

The *in vitro* H_2_O_2_ concentration was determined by Amplex Red Hydrogen Peroxide/Peroxidase Assay Kit. The detection limit of the assay was 0.1 μM. Cells were seeded in six-well plates without phenol red medium, and after 24 h from seeding, they were treated with **L2**, **L4**, and the related Ni complexes. H_2_O_2_ was measured in the supernatant cell collected after 30 min, 90 min, 3 h, and 24 h according to the manufacturer’s instructions.

Oxygen partial pressure was followed over time with a Clark electrode kinetic analysis, which was conducted with a Knick SE715 Memosens oxygen sensor (Berlin, Germany) plugged into a Knick Portavo 907 Multi meter (Berlin, Germany). Clark-like electrode *in vial* measurements were carried out in PBS buffer (pH 7.4, [PBS] = 10 mM, [NaCl] = 137 mM, [KCl] = 2.7 mM) with 5% DMSO in 50 mL centrifuge tubes. H_2_O_2_ stock solution at 880 mM in double-distilled water, **Ni2** stock solution in DMSO at 1.25 mM, and **Ni4** stock solution at 1 mM were prepared. Considering a total volume of 10 mL, additions of complex solutions were tuned to have a total 50 μM concentration, whereas the H_2_O_2_ final concentration was 1.25, 2.5, 3.75, 5, 7.5, 12.5, and 15 mM. After having purged the PBS solution with dinitrogen gas to reduce the dissolved oxygen concentration under 1.5 mg/L (47 μM), complexes and H_2_O_2_ were added and O_2_ was measured over time for 15–20 min.

The initial rates *V*_0_ were determined from the slope of the linear fit (from 0 to 20 s) of dioxygen formation at different initial H_2_O_2_ concentrations. Michaelis–Menten constants *K*_m_ and *V*_max_ were determined using a Michaelis–Menten fitting model on Origin 2019 64 bit.

$$V_{0}=\frac{V_{\max}[H_{2}O_{2}{]}_{0}}{K_{M}+[H_{2}O_{2}{]}_{0}}$$ 7

To obtain the enzymatic turnover number (also called catalytic constant for enzymes with a single active site, *k*_cat_),^81^ we employed the following equivalence:

$$V_{\max}=k_{\mathrm{cat}-O_{2}\mathrm{production}}[\mathrm{catalyst}{]}_{0}$$ 8

Since dismutation leads from two H_2_O_2_ molecules to a single O_2_ molecule:

$$k_{\mathrm{cat}-H_{2}O_{2}\mathrm{consumption}}=2k_{\mathrm{cat}-O_{2}\mathrm{production}}$$ 9

The experimental procedure was adapted mostly from a previous work by Ben Hadj Hammouda et al.^52^

##### Gene Expression

cDNA was synthesized using a commercial kit based on the use of inverse transcriptase, following the manufacturer’s recommended experimental conditions [High-Capacity RNA-to-cDNA kit (Applied Biosystems; Thermo Fisher Scientific Inc., Waltham, Massachusetts, United States)]. RT-qPCR was performed using the QuantStudio 7 Flex Real-Time PCR System (Thermo Fisher Scientific Inc., Waltham, Massachusetts, United States) using specific primers, including exon–exon junctions specifically designed for heme oxygenase 1 (HO-1), superoxide dismutase 1 (SOD-1), and superoxide dismutase 2 (SOD-2). TP53 gene expression was quantified using TaqMan gene expression assays (Assay ID: Hs01034249_m1; Thermo Fisher Scientific, Waltham, Massachusetts, USA). The reactions consisted of one step at 95 °C for 10 min, followed by 40 cycles of 95 °C for 15 s and 60 °C for 1 min. All assays were performed in duplicate, and one no-template and two interpolate controls were used in each experiment. Data were normalized over the phosphoglycerate kinase 1 (PGK1) housekeeping gene, and the expressions were calculated as 2^–ΔΔCt^. One-way analysis of variance (ANOVA) with Dunnett’s or Tukey’s post hoc tests was used for data comparison. RNA from treated and untreated cultured cells was extracted using TRIzol reagent (Thermo Fisher Scientific Inc., Waltham, Massachusetts, United States), following the manufacturer’s instructions. Subsequently, to eliminate genomic DNA contamination a DNase I (DNA-free kit; Thermo Fisher Scientific, Waltham, Massachusetts, USA) was used, and the RNA concentration was determined using a Varioskan LUX (Thermo Fisher Scientific Inc., Waltham, Massachusetts, United States).

## Supplementary Material



## Notes

General: materials and methods; synthesis and characterization of the ligands (**L1**, **L2**, **L3**, and **L4**) and of the complexes (**Ni1**, **Ni2**, **Ni3**, and **Ni4**); diffraction characterization (**L4**, **Ni1**, **Ni2**, **Ni3**, and **Ni4**); electronic spectroscopy; cyclic voltammetry; computational analysis; cytotoxicity assays: IC_50_ fit curves; interactions with albumin; *Galleria mellonella* larval model; albumin affinity: circular dichroism assays; fluorescence titrations; interactions with DNA: ethidium bromide displacement titrations; and reactive oxygen species pathway: Clark-type oximetry (PDF)

## Abbreviations

ACN: acetonitrile
BSA: bovine serum albumin
CD: circular dichroism
CT-DNA: calf thymus DNA
DCFH-DA: 2,7-dichlorodihydrofluorescein diacetate
DCM: dichloromethane
DMF: dimethylformamide
DPPH: diphenylpicrydrazyl
EthBr: ethidium bromide
HOMO: highest occupied molecular orbital
HSA: human serum albumin
IC50: inhibitory concentration 50%
LPO: lipid peroxides
LUMO: lowest unoccupied molecular orbital
MDR: multidrug resistance
PBS: phosphate buffer solution
ROS: reactive oxygen species
RSA: radical scavenging activity
SI: selectivity index
TBARS: thiobarbituric acid reactive substances
TSC: thiosemicarbazone.
