# Supplementary material for: Multimodal Mechanism of Antitumoral Ni(II) Thiosemicarbazones: Deep Mechanistic Understanding of ROS Dynamics, Albumin-Mediated Delivery, and DNA Targeting
Source: ACS Omega. 2026 May 29;11(22):32118–40. doi: 10.1021/acsomega.5c12067 (PMC13261419; doi:10.1021/acsomega.5c12067)
Supplement: Supplementary file 1 [file ao5c12067_si_001.pdf]

Multi-modal Mechanism of Antitumoral Ni(II) Thiosemicarbazones: Deep Mechanistic Understanding of ROS Dynamics, Albumin-Mediated Delivery, and DNA Targeting

Lorenzo Verderi [1], Silvana Pinelli[2], Gloria Cenci[1,3], Chiara Maccari [2], Paola Mozzoni[2, 4], Simone Fortunati[1], Marco Giannetto[1], Valentina Borghesani[1], Matteo Tegoni[1], Luca Ronda[2], Jesus Diaz[5], Mauro Carcelli[1], Giorgio Pelosi[1, 4] and Franco Bisceglie[1, 4]*

[1]Department of Chemistry, Life Sciences and Environmental Sustainability, University of Parma, Parco Area delle Scienze 17/a 43124 Parma, Italy. [2]Department of Medicine and Surgery, University of Parma, Via Gramsci 14, 43125 Parma, Italy. [3]Institute of Materials for Electronics and Magnetism, National Research Council (IMEM-CNR), Parco Area delle Scienze 37/a, 43124 Parma, Italy. [4]Center of Excellence for Toxicological Research (CERT), University of Parma, Via Gramsci 14, 43126 Parma, Italy. [5]Laboratory of Bioorganic Chemistry & Membrane Biophysics (L.O.B.O.). Departamento de Química Orgánica e Inorgánica. Universidad de Extremadura. 10003 Cáceres, Spain.

Table of Contents

1. General p. 2
2. Synthesis and Charaterization p. 5
    2. 1.Ligands (L1, L2, L3, L4) p. 5
    2. 2.Complexes (Ni1, Ni2, Ni3, Ni4) p. 14
    2. 3.X-ray Diffraction Characterization (L4, Ni1, Ni2, Ni3, Ni4) p. 23
    2. 4.Electronic Spectroscopy p. 28
    2. 5.Cyclic Voltammetry p. 33
    2. 6.Computational Analysis p. 33
3. CytotoxicityAssays: IC_{50} fit curves p. 37
4. Interactions with Albumin p. 45
    4.1 *Galleria mellonella* larval model p. 45
    4.2 Albumin affinity: circular dichroism assays p. 46
    4.3 Albumin affinity: fluorescence titrations p. 47
5. Interactions with DNA: ethidium bromide displacement titrations p. 48
6. Reactive Oxygen Species pathway: Clark-type oximetry p. 52
7. References p.52

1. GENERAL

All common laboratory chemicals were purchased from commercial sources and used without further purification: 3,4-dimethoxybenzaldehyde, ≥99.0% (Janssen Chimica, Geel, Belgium); thiosemicarbazide, ≥99.9% (Fluka, Buchs, Switzerland); 4-methyl-3-thiosemicarbazide, 97% (Fluorochem, Hadfield, UK); 4-phenyl-3-thiosemicarbazide, 99% (Sigma-Aldrich, Burlington, MA, USA); 4-(2-morpholinoethyl)-3-thiosemicarbazide (Fluorochem, Hadfield, UK); nickel(II) acetate, 99% (Carlo Erba, Milano); ethidium bromide, 10 mg/mL solution in water (Sigma-

Aldrich, Burlington, MA, USA); lyophilized calf thymus DNA (SERVA Electrophoresis GmbH, Heidelberg, Germany); bovine serum albumin, 98% (VWR, Phillipsburg, NJ, USA); hydrogen peroxide 30% in water (Sigma-Aldrich, Burlington, MA, USA); 20 μm Ø microfilters (Eppendorf, Hamburg, Germany); phosphate buffered solution tablets (VWR, Phillipsburg, NJ, USA).

NMR spectra were recorded on a Bruker Anova spectrometer at 400 MHz (Billerica, MA, USA), with chemical shifts reported in δ units (ppm). The NMR spectra were referenced relative to the residual solvent peaks. The solvent used for the spectra acquisitions was DMSO-d6. FT-IR measurements were recorded on a Nicolet 5PC FT-IR spectrometer (Rodano, MI, Italy) in the range of 4000–400 cm^{-1} and equipped with an ATR accessory. Elemental analyses were performed using the ThermoFisher Scientific FlashSmart CHNS Elemental Analyzer (Rodano, MI, Italy). ESI-MS data were collected on a Waters Acquity Ultraperformance ESI-MS spectrometer with a Single Quadrupole Detector (Sesto San Giovanni, MI, Italy). UV/Visible spectra were obtained using a ThermoFisher Scientific Evolution 260 Bio Spectrophotometer (Rodano, MI, Italy) and a Perkin-Elmer Lambda 465 (Milano, Italy), utilizing quartz cuvettes with a 1 cm path length. Oxygen partial pressure was followed over time with a Clark electrode kinetic analysis was conducted with a Knick SE715 Memosens oxygen sensor (Berlin, Germany) plugged into a Knick Portavo 907 Multi meter (Berlin, Germany). Fluorescence spectra were collected with an FLS1000 Edinburgh Instruments fluorometer (Edinburgh, Scotland, UK) equipped with a 450 W Xenon lamp as the excitation source, using standard 1 cm x 1 cm 3 mL

quartz cuvettes. To minimize inner-filter effects, samples with optical densities < 0.1 were analyzed. Cyclic voltammetry (CV) experiments were carried out using a μStat 8000 Multi Potentiostat/Galvanostat (Metrohm Italiana S.r.l., Origgio, Italy) on screen-printed electrodes featuring carbon working and counter electrodes and a silver pseudoreference electrode (SPCE DRP-C110, Metrohm Italiana S.r.l., Origgio, Italy). Data acquisition and elaboration were conducted using DropView 8400 software (version 3.78). Circular dichroism measurements were performed using a J-1500 spectrophotometer (JASCO Corporation, Tokyo, Japan). Galleria mellonella larvae were purchased from Fishing & Adventure S.r.l. (Parma, Italy).

Cell lines used for biological experiments were A549, HT29, H2052, HL60 (ATCC, Rockville, MD, United States), using non-neoplastic human dermal fibroblast cell line HuDe (Istituto Zooprofilattico Sperimentale della Lombardia e dell'Emilia (IZSLE), Brescia, Italy) for selectivity analysis. Varioskan Lux (Thermo Fisher Scientific Inc., Waltham, MA, United States) was used to perform MTT assay, DNA quantification, thiobarbituric acid and reactive substance (TBARS) assay, the hydrogen peroxide assay on the cell supernatant using Amplex® Red Hydrogen Peroxide/Peroxidase Assay Kit (Invitrogen; Thermo Fisher Scientific, Inc. Waltham, MA, United States) and the enzymatic activity of the caspase-3 using Caspase-Glo 3/7 assay (Promega Corporation, Madison, WI, USA), according to manufacturer's instructions. CitoFlex flow cytometer (Beckman Coulter, Brea, CA, United States) was used to perform intracellular reactive oxygen species measurement using DCFH-DA, and the apoptosis assay using Annexin V/FITC Assay Kit (Invitrogen; Thermo Fisher Scientific, Inc. Waltham, MA, United States). DNA extraction was performed using Gentra Puregene (QIAGEN, Venlo, The Netherlands).

RNA from treated and untreated cultured cells was extracted using TRIzol reagent (Thermo Fisher Scientific Inc., Waltham, MA, United States), following the manufacturer's instructions. Subsequently, to eliminate genomic DNA contamination a DNase I (DNA-free kit; Thermo Fisher Scientific, Waltham, MA, USA) was used, and the RNA concentration was determined using a Varioskan Lux (Thermo Fisher Scientific Inc., Waltham, MA, United States). cDNA was synthesized using a commercial kit based on the use of inverse transcriptase, [High-Capacity RNA-to-cDNA™ kit (Applied Biosystems; Thermo Fisher Scientific Inc., Waltham, MA, United States)], following the manufacturer's recommended experimental conditions. RT-qPCR was performed using the QuantStudio 7 Flex Real-Time PCR System (Thermo Fisher Scientific Inc., Waltham, MA, United States) using specific primers, including exon–exon junctions specifically designed for heme oxygenase 1 (HO-1), superoxide dismutase 1 (SOD-1), and superoxide dismutase 2 (SOD-2). TP53 gene expressions were quantified using Taqman gene expression assays (Assay ID: Hs01034249_m1; Thermo Fisher Scientific, Waltham, MA, USA).

The software applications used for data interpretation are: GraphPad Prism 8.0.2 (263) (GraphPad Software Inc., San Diego, CA, United States), Origin 2019 64bit (OriginLab Corporation, Northampton, MA, United States), SkanIt RE 7.1 (Thermo Fisher Scientific, Inc. Waltham, MA, United States), FlowJo 10.9 Software (Tree Star Inc., Ashland, OR, United States). Biological results are expressed as means ± SD of at least three independent experiments. Statistical analysis on biological results was performed by (SPSS Inc/IBM, Chicago, Ill, USA), using one-way ANOVA with Dunnett's or Tukey's post hoc tests. p-values<0.05 (two-sided) were considered as statistically significant.

2. SYNTHESIS AND CHARACTERIZATION

2.1 Ligands (L1, L2, L3, L4)

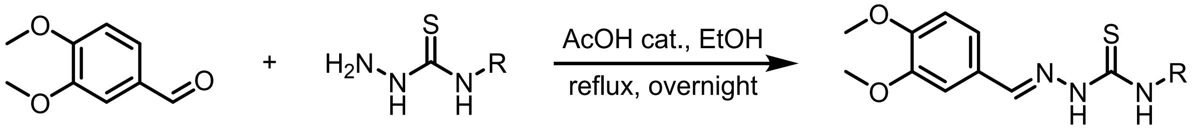

L1-4

We followed the same synthetic protocol for all the ligands[1–3].

The thiosemicarbazide (1.25 mmol; unsubstituted, *N*-methyl, *N* -phenyl-, *N* -(2-morpholinoethyl)-) was mixed in a 1:1 stoichiometry with 3,4-dimethoxybenzaldehyde (1.25 mmol) in ethanol (15 mL), and a catalytic amount (few drops) of acetic acid was added. The mixture was refluxed overnight and then the mixture was cooled to 4°C to promote precipitation. The resulting solid was filtered, washed with diethyl ether and dried at the vacuum line.

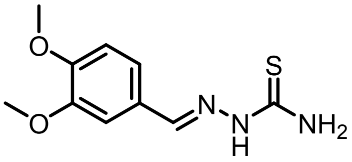

L1

(E)-2-(3,4-dimethoxybenzylidene)hydrazine-1-carbothioamide

$C_{10}H_{13}N_3O_2S$ 239.29 g/mol

Yield: 86%

Appearance: white powder.

Elemental Analysis $C_{10}H_{13}N_3O_2S$ Calcd.: C 50.19%, N 17.56%, H 5.48%, S 13.40% Exp.: C 49.84%, N 16.84%, H 5.56%, S 13.07%. ^{1}H NMR (400 MHz, DMSO-d_6): [ppm] 11.27 (s, 1H, C=N-NH); 8.09 (s, 1H, NH_2); 7.97 (s, 1H, CH=N); 7.96 (s, 1H, NH_2); 7.50 (d, 1H, CH_{arom}); 7.14 (dd, 1H, CH_{arom}); 6.95 (d, 1H, CH_{arom}); 3.82 (s, 3H, CH_3); 3.79 (s, 3H, CH_3). ^{13}C NMR (101 MHz, DMSO-d_6): [ppm] 177.7, 150.7, 149.2, 142.6, 127.0, 122.1, 111.4, 108.7, 55.6. IR (ATR, cm^{-1}): 3350, 3260 w (N-H), 3116 m (C-$H_{arom.}$), 2960 m (C-$H_{aliph.}$), 1617 m (C=N), 1096, 853 m (C=S). ESI-MS (*m/z*, %): 240 ([M+H]$^+$, 60).

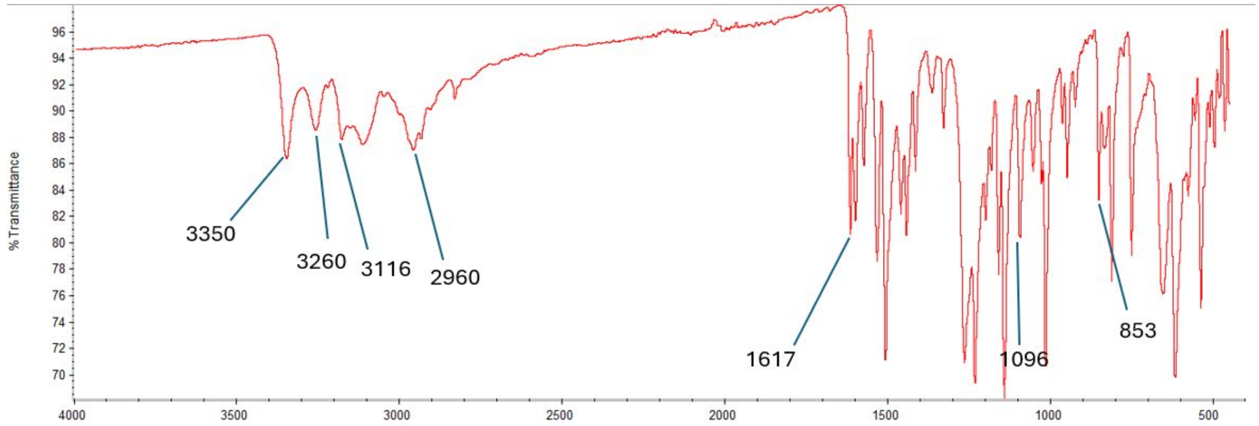

Figure S1: IR (ATR, cm-1): 3350, 3260 w (N-H), 3116 m (C-Harom.), 2960 m (C-Haliph.), 1617 m (C=N), 1096, 853 m (C=S).

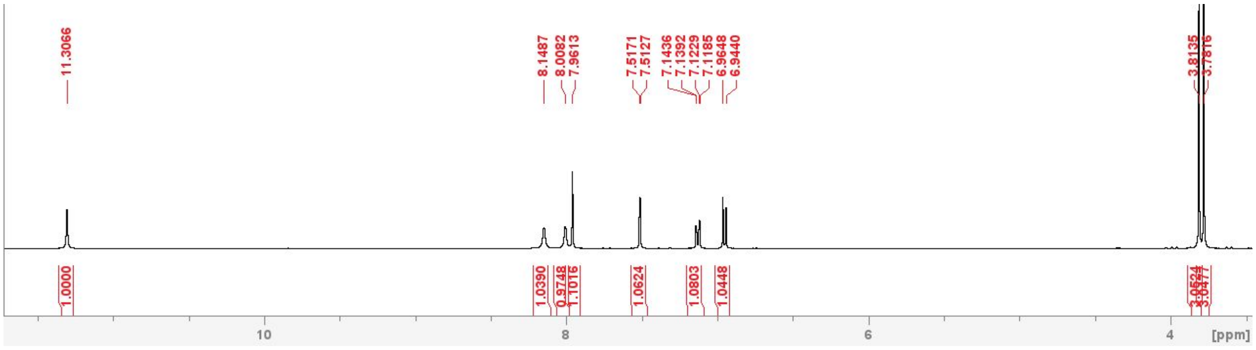

Figure S2: ^{1}H NMR (400 MHz, DMSO-d_6): [ppm] 11.27 (s, 1H, C=N-NH); 8.09 (s, 1H, NH_2); 7.97 (s, 1H, CH=N); 7.96 (s, 1H, NH_2); 7.50 (d, 1H, CH_{arom}); 7.14 (dd, 1H, CH_{arom}); 6.95 (d, 1H, CH_{arom}); 3.82 (s, 3H, CH_3); 3.79 (s, 3H, CH_3).

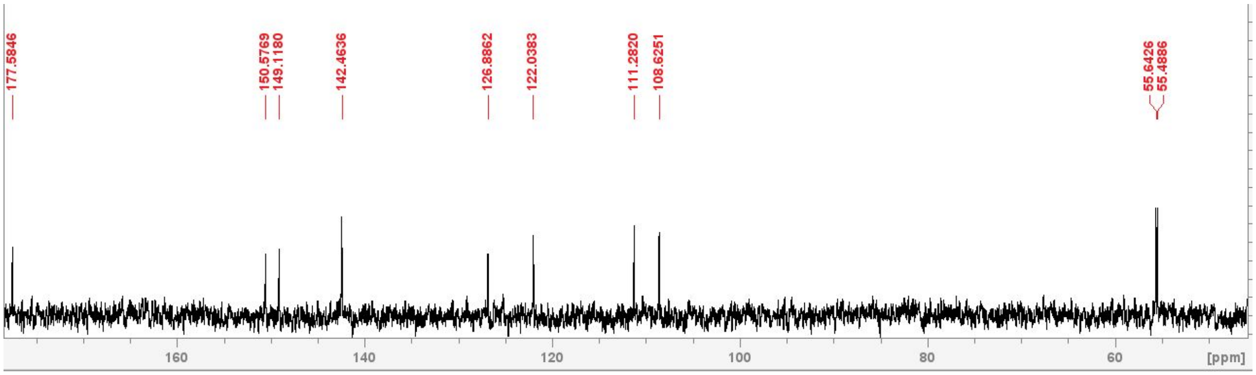

Figure S3: ^{13}C NMR (101 MHz, DMSO-d_6): [ppm] 177.7, 150.7, 149.2, 142.6, 127.0, 122.1, 111.4, 108.7, 55.6.

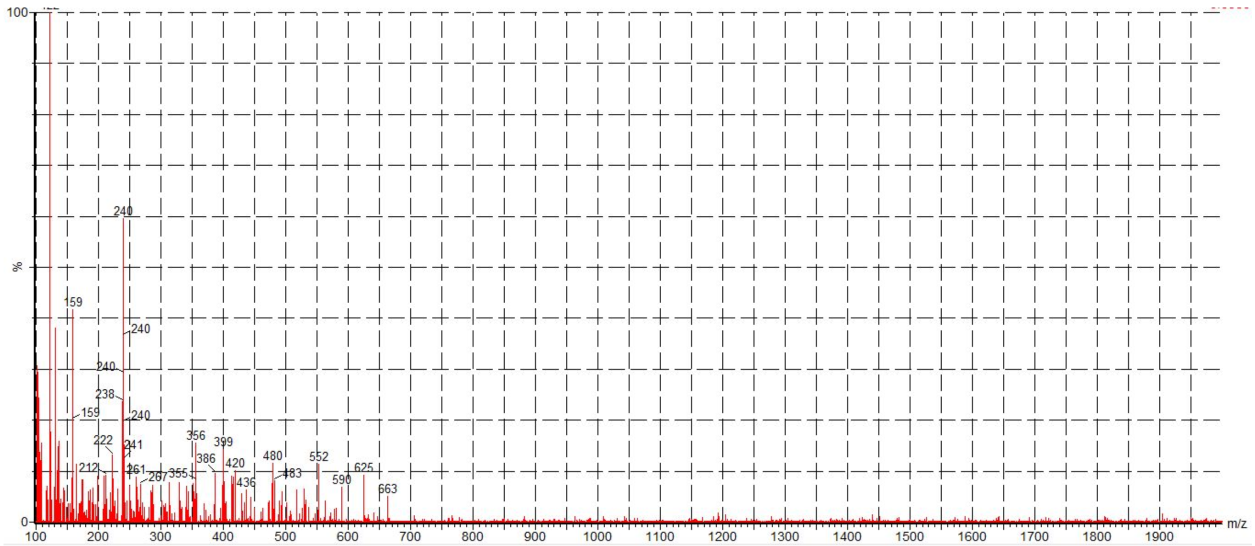

Figure S4: ESI-MS (*m/z*, %): 240 ([M+H]$^+$, 60).

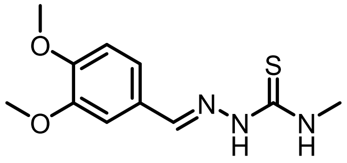

L2

(E)-2-(3,4-dimethoxybenzylidene)-N-methylhydrazine-1-carbothioamide

$C_{11}H_{15}N_3O_2S$ 253.32 g/mol

Yield: 83%.

Appearance: white powder.

Elemental Analysis $C_{11}H_{15}N_3O_2S$ Calcd.: C 52.16%, N 16.59%, H 5.97%, S 12.66% Exp.: C 51.74%, N 15.91%, H 6.00%, S 12.35%. ^{1}H NMR (400 MHz, DMSO-d6): [ppm] 11.33 (s, 1H, C=N-NH); 8.38 (m, 1H, NH(CH_3)); 7.98 (s, 1H, CH=N); 7.45 (d, 1H, CH_{arom}); 7.21 (dd, 1H, CH_{arom}); 6.98 (d, 1H, CH_{arom}); 3.84 (s, 3H, CH_3); 3.80 (s, 3H, CH_3); 3.04 (d, 3H, NH(CH_3)). ^{13}C NMR (101 MHz, DMSO-d6): [ppm] 177.6, 150.6, 149.1, 142.0, 127.0, 121.8, 111.5, 109.1, 56.0, 30.9. IR (ATR, cm^{-1}): 3352, 3149 m (N-H), 2990 m (C-$H_{arom.}$), 2962 w (C-$H_{aliph.}$), 1602 m (C=N), 1084, 862 m (C=S). ESI-MS (m/z, %): 254 ($[M+H]^+$, 79).

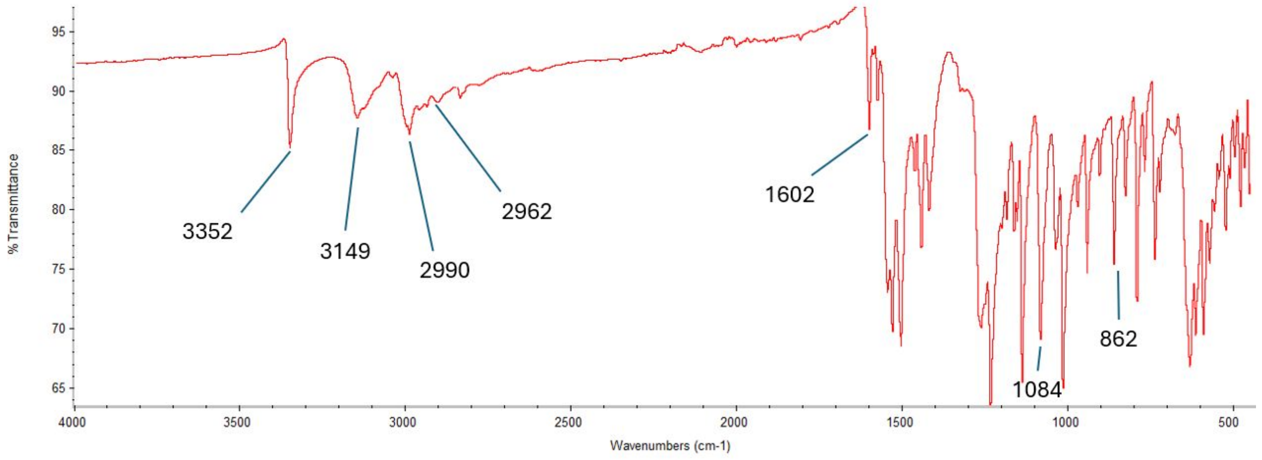

Figure S5: IR (ATR, cm^{-1}): 3352, 3149 m (N-H), 2990 m (C-$H_{arom.}$), 2962 w (C-$H_{aliph.}$), 1602 m (C=N), 1084, 862 m (C=S).

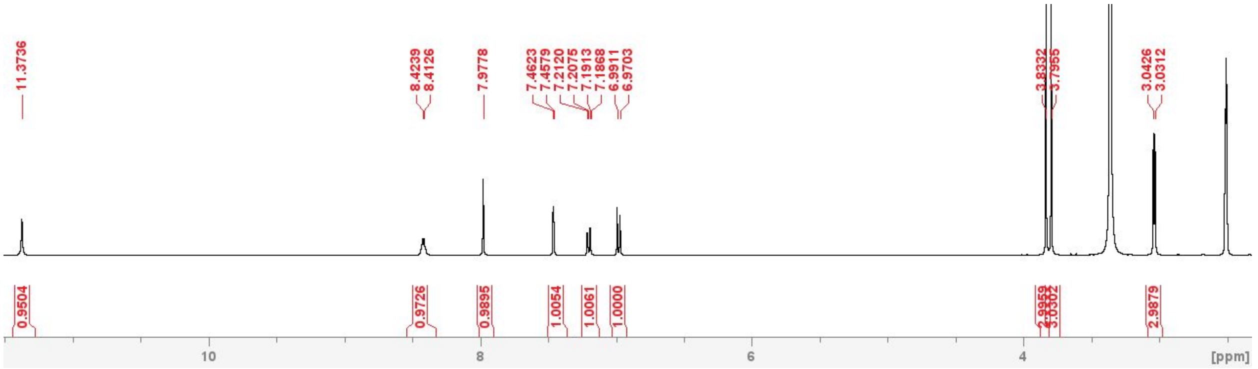

Figure S6: ^{1}H NMR (400 MHz, DMSO-d_6): [ppm] 11.33 (s, 1H, C=N-NH); 8.38 (m, 1H, **NH**(CH_3)); 7.98 (s, 1H, CH=N); 7.45 (d, 1H, CH_{arom}); 7.21 (dd, 1H, CH_{arom}); 6.98 (d, 1H, CH_{arom}); 3.84 (s, 3H, CH_3); 3.80 (s, 3H, CH_3); 3.04 (d, 3H, NH(**CH_3**)).

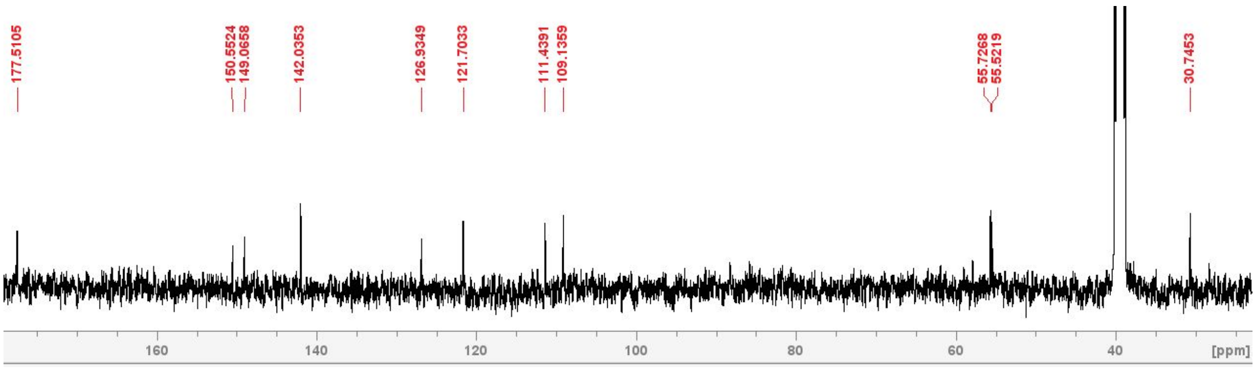

Figure S7: ^{13}C NMR (101 MHz, DMSO-d_6): [ppm] 177.6, 150.6, 149.1, 142.0, 127.0, 121.8, 111.5, 109.1, 56.0, 30.9.

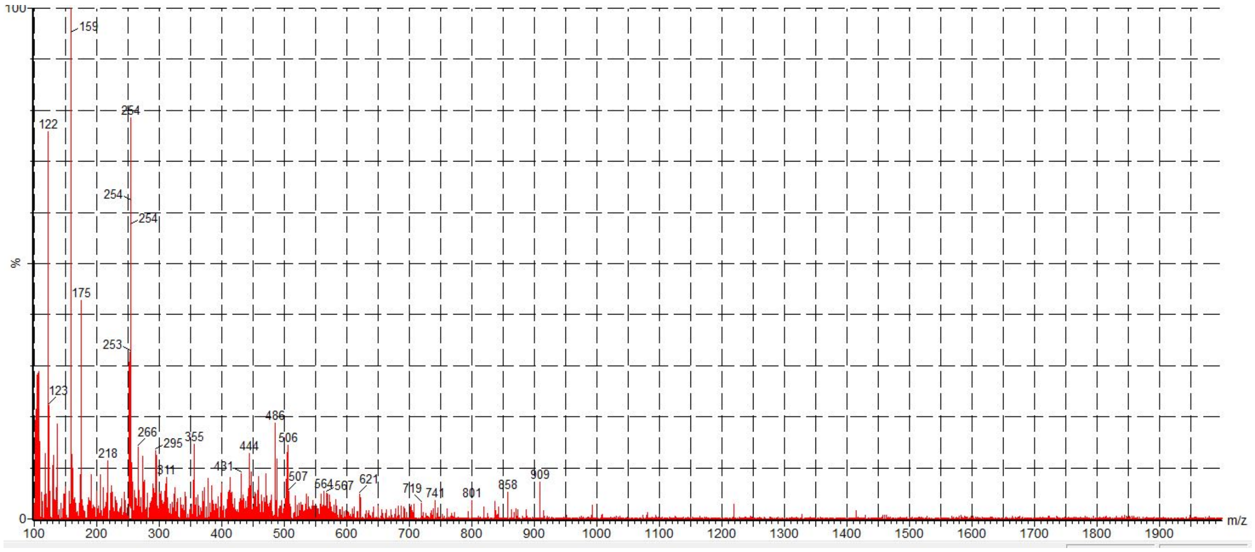

Figure S8: ESI-MS (*m/z*, %): 254 ([M+H]$^+$, 79).

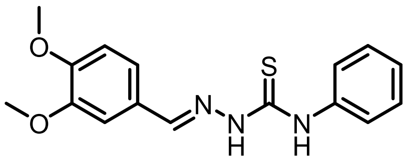

L3

(E)-2-(3,4-dimethoxybenzylidene)-N-phenylhydrazine-1-carbothioamide

$C_{16}H_{17}N_3O_2S$ 315.39 g/mol

Yield: 92%.

Appearance: white-yellow powder.

Elemental Analysis Calcd.: C 60.93%, N 13.32%, H 5.43%, S 10.17% Exp.: C 60.44%, N 12.79%, H 5.47%, S 10.06%. ^{1}H NMR (400 MHz, DMSO-d_6): [ppm] 11.73 (s, 1H, C=N-NH); 10.02 (s, 1H, S=C-NH-ph); 8.09 (s, 1H, CH=N); 7.56 (m, 3H, $CH_{arom.ald.}$); 7.37 (t, 2H, CH_{arom}); 7.27 (dd, 1H, $CH_{arom.ald}$); 7.21 (t, 1H, CH_{arom}); 6.99 (d, 1H, $CH_{arom.ald}$); 3.83 (s, 3H, CH_3); 3.80 (s, 3H, CH_3). ^{13}C NMR (101 MHz, DMSO-d$_6$): [ppm] 175.8, 161.3, 159.3, 151.0, 149.2, 143.3, 139.3, 128.2, 126.7, 126.0, 125.4, 122.4, 111.7, 109.4, 55.6. IR (ATR, cm^{-1}): 3336, 3311 w (N-H), 3142, 2993 m (C-$H_{arom.}$), 2953 w (C-$H_{aliph.}$), 1598 m (C=N), 1072, 862 m (C=S). ESI-MS (*m/z*, %): 316 ([M+H]$^{+}$, 82).

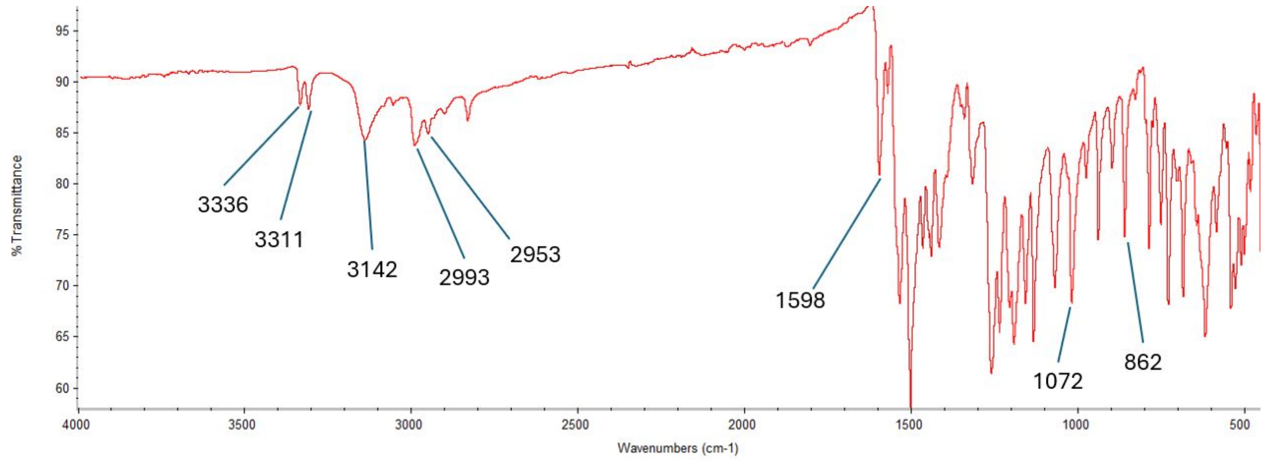

Figure S9: IR (ATR, cm^{-1}): 3336, 3311 w (N-H), 3142, 2993 m (C-$H_{arom.}$), 2953 w (C-$H_{aliph.}$), 1598 m (C=N), 1072, 862 m (C=S).

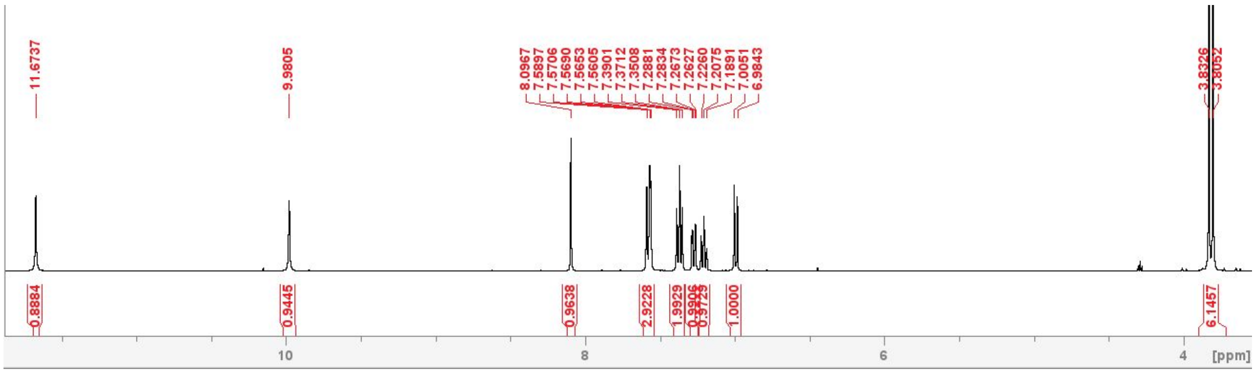

Figure S10: ^{1}H NMR (400 MHz, DMSO-d_6): [ppm] 11.73 (s, 1H, C=N-NH); 10.02 (s, 1H, S=C-NH-ph); 8.09 (s, 1H, CH=N); 7.56 (m, 3H, $CH_{arom.ald.}$); 7.37 (t, 2H, CH_{arom}); 7.27 (dd, 1H, $CH_{arom.ald}$); 7.21 (t, 1H, CH_{arom}); 6.99 (d, 1H, $CH_{arom.ald}$); 3.83 (s, 3H,

CH$_3$); 3.80 (s, 3H, CH$_3$).

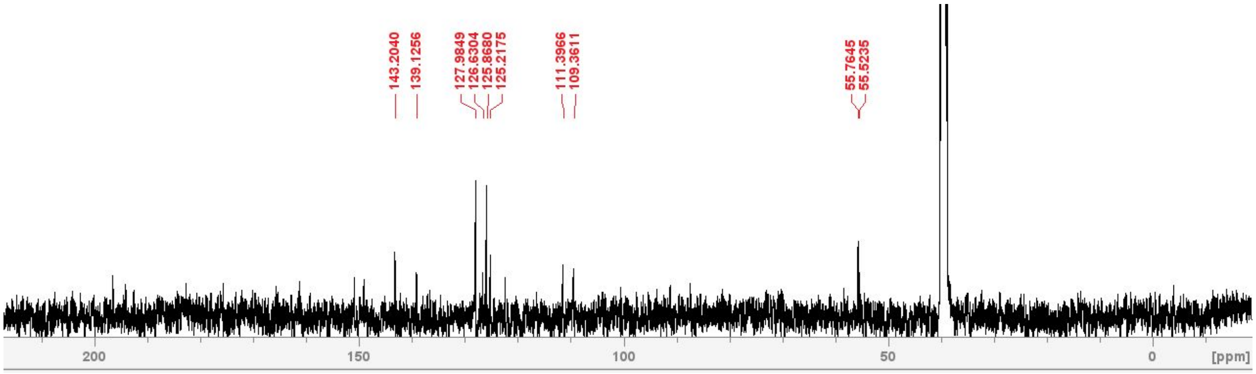

Figure S11: ^{13}C NMR (101 MHz, DMSO-d$_6$): [ppm] 175.8, 161.3, 159.3, 151.0, 149.2, 143.3, 139.3, 128.2, 126.7, 126.0, 125.4, 122.4, 111.7, 109.4, 55.6.

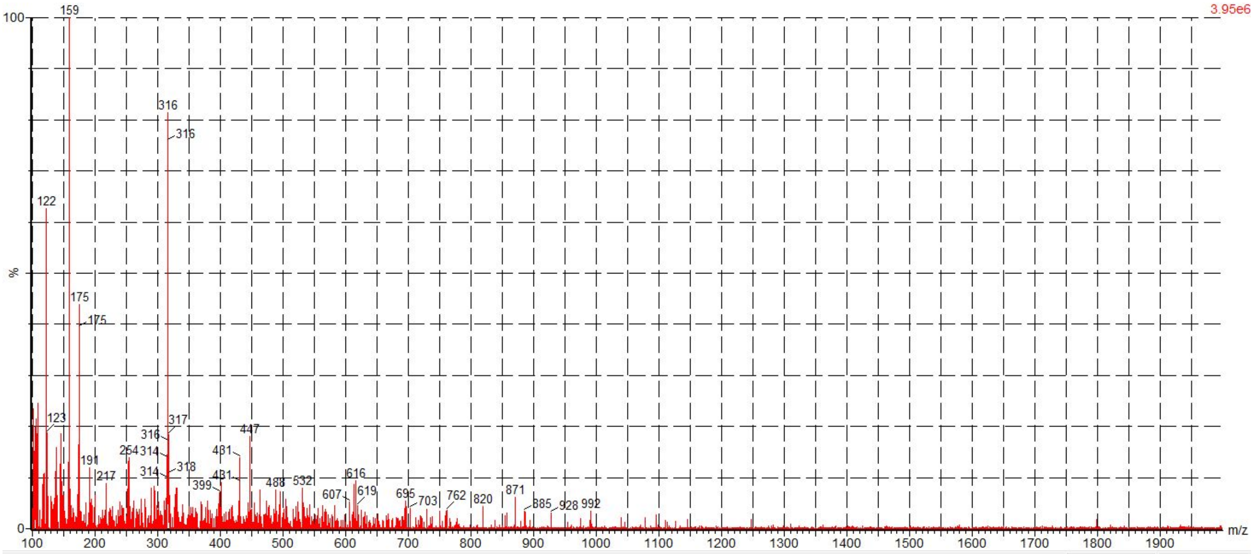

Figure S12: ESI-MS (*m/z*, %): 316 ([M+H]$^+$, 82).

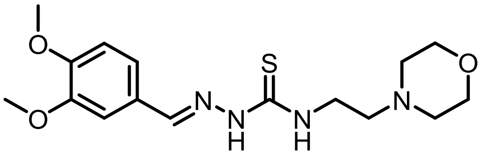

L4

(E)-2-(3,4-dimethoxybenzylidene)-N-(2-morpholinoethyl)hydrazine-1-carbothioamide

$C_{16}H_{24}N_4O_3S$ 352.45 g/mol

Yield: 92%.

Appearance: at first, yellow oil. After several trituration passages with diethyl ether, white-yellow powder.

Elemental Analysis Calcd.: C 54.53%, N 15.90%, H 6.86%, S 9.00% Exp.: C 54.59%, N 14.96%, H 6.83%, S 9.03%. ^{1}H NMR (400 MHz, DMSO-d_6): [ppm] 11.42 (s, 1H, C=N-NH); 8.31 (9.98 (s, 1H, S=C-NH-R); 8.00 (s, 1H, CH=N); 7.37 (m, 3H, $CH_{arom.}$); 7.22 (dd, 1H, $CH_{arom.}$); 6.99 (d, 1H, $CH_{arom.}$); 3.83 (s, 3H, CH_3); 3.80 (s, 3H, CH_3); 3.68 (m, 2H, S=C-NH-C**H_2**-); 3.58 (t, 4H, -CH_2-O-CH_2); 2.54 (m, 2H, S=C-NH-CH_2-C**H_2**-N); 2.44 (m, 4H, -CH_2-N-CH_2). ^{13}C NMR (101 MHz, DMSO-$_{d6}$): [ppm] 176.7, 150.7, 149.1, 142.5, 126.8, 121.7, 111.5, 109.1, 66.4, 56.6, 55.8, 53.2. IR (ATR, cm^{-1}): 3341 w, 3229 b (N-H), 2958, 2908, 2887, 2860, 2837, 2814 m (C-H), 1601 m (C=N), 1068, 847 m (C=S). ESI-MS (m/z, %): 353 ($[M+H]^+$, 100).

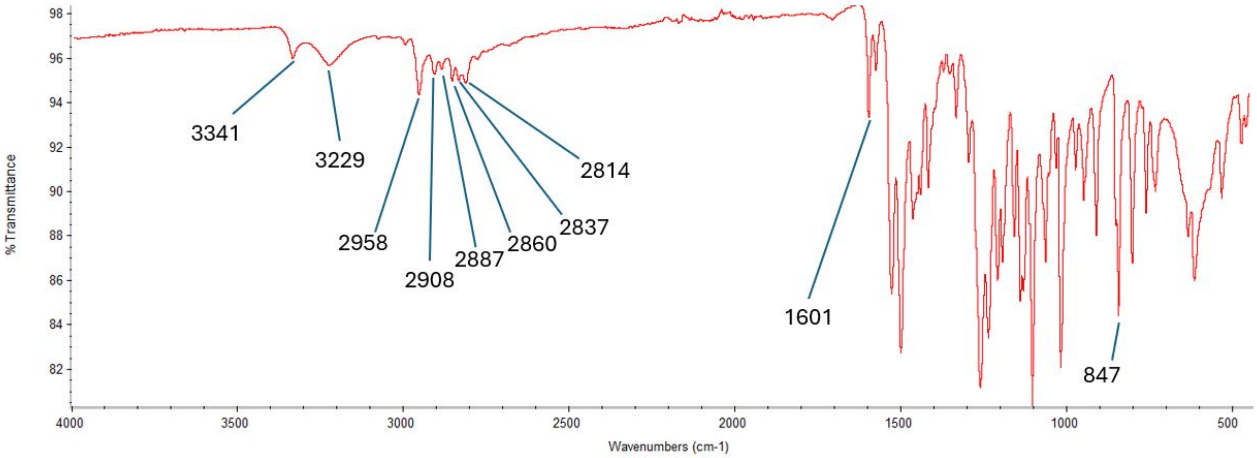

Figure S13: IR (ATR, cm^{-1}): 3341 w, 3229 b (N-H), 2958, 2908, 2887, 2860, 2837, 2814 m (C-H), 1601 m (C=N), 1068, 847 m (C=S).

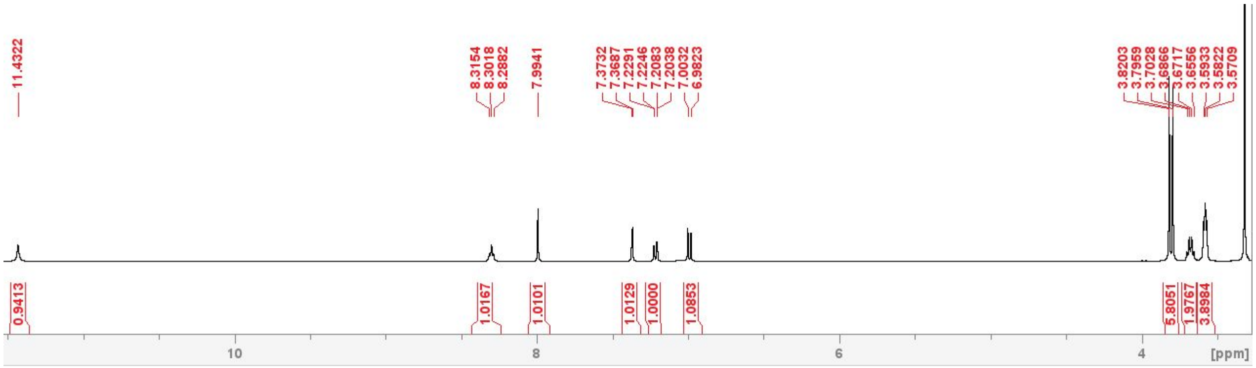

Figure S14: ^{1}H NMR (400 MHz, DMSO-d_6): [ppm] 11.42 (s, 1H, C=N-NH); 8.31 (9.98 (s, 1H, S=C-NH-R); 8.00 (s, 1H, CH=N); 7.37 (m, 3H, $CH_{arom.}$); 7.22 (dd, 1H, $CH_{arom.}$); 6.99 (d, 1H, $CH_{arom.}$); 3.83 (s, 3H, CH_3); 3.80 (s, 3H, CH_3); 3.68 (m, 2H, S=C-NH-CH_2-); 3.58 (t, 4H, -CH_2-O-CH_2); 2.54 (m, 2H, S=C-NH-CH_2-CH_2-N); 2.44 (m, 4H, -CH_2-N-CH_2).

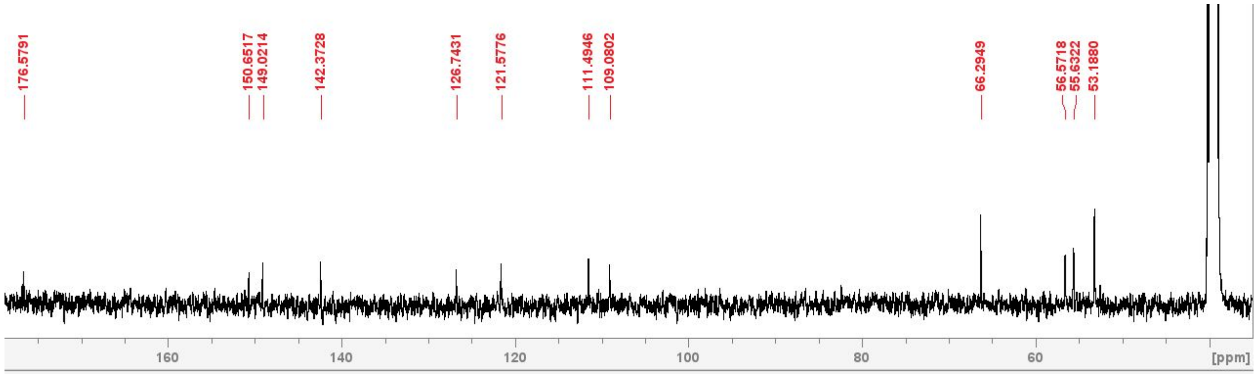

Figure S15: ^{13}C NMR (101 MHz, DMSO-$_{d6}$): [ppm] 176.7, 150.7, 149.1, 142.5, 126.8, 121.7, 111.5, 109.1, 66.4, 56.6, 55.8, 53.2.

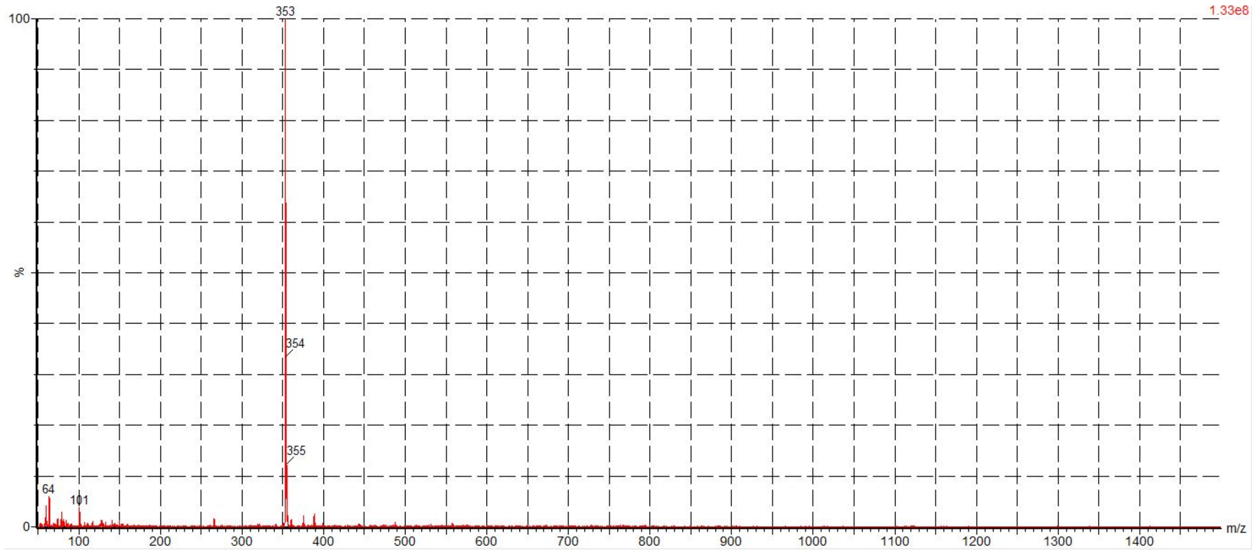

Figure S16: ESI-MS (m/z, %): 353 ($[M+H]^+$, 100).

2.2 Complexes (Ni1, Ni2, Ni3, Ni4)

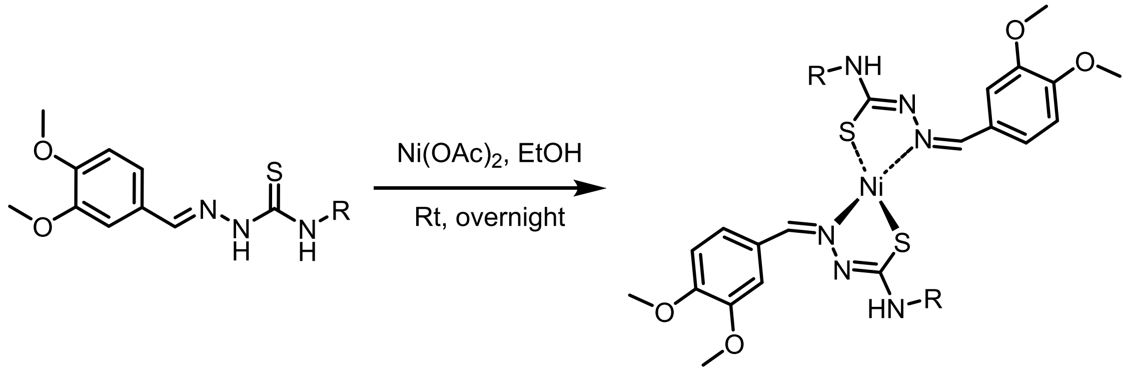

Ni1-4

The same synthetic procedure was performed for all the complexes. We added the ligand (0.37 mmol) to refluxing ethanol (10 mL) and stirred for 1-2 hours. Afterwards, a solution of $Ni(OAc)_2 \cdot 4H_2O$ (0.19 mmol) was added to the main mixture, which was cooled to room temperature and left stirring overnight. The formation of the Ni(II) complexes was accompanied by an intense color shift to yellow/light brown. The Ni(II) complexes were precipitated, isolated, and eventually washed with diethyl ether.

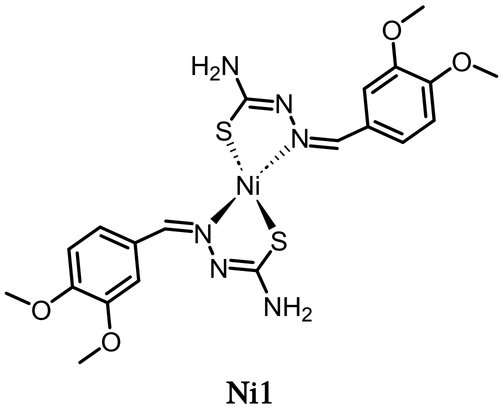

Ni1

$C_{20}H_{28}N_6NiO_4S_2$ 535.26 g/mol

Yield: 79%.

Appearance: light brown powder.

Elemental analysis Calcd.: C 44.88%, N 15.70%, H 4.52%, S 11.98% Exp.: C 44.92%, N 15.60%, H 4.62%, S 11.98%. ^{1}H NMR (400 MHz, DMSO-d_6): [ppm] 8.74 (s, 1H, NH_2); 7.26 (s, 1H, CH=N); 7.04 (s, 1H, NH_2); 7.00 (d, 1H, CH_{arom}); 6.59 (s, 2H, CH_{arom}); 4.03 (s, 3H, CH_3); 3.83 (s, 3H, CH_3). IR (ATR, cm^{-1}): 3426, 3289 w (N-H), 3177, 3139, 2952, 2911, 2832 w (C-H), 1626 m (C=N), 1019 s, 794 m (C=S). ESI-MS (*m/z*, %): 535 ([M+H]$^+$, 100).

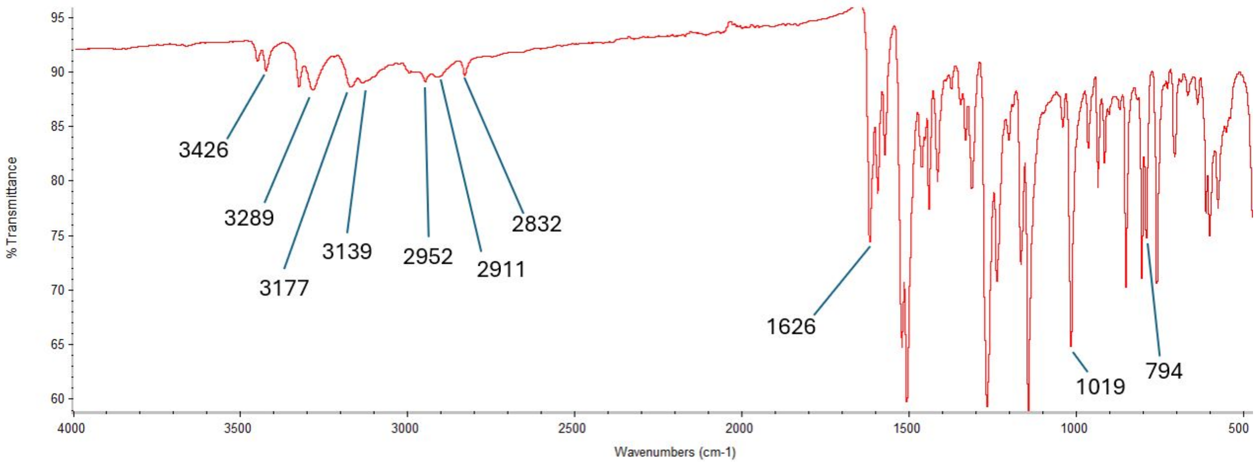

Figure S17: IR (ATR, cm^{-1}): 3426, 3289 w (N-H), 3177, 3139, 2952, 2911, 2832 w (C-H), 1626 m (C=N), 1019 s, 794 m (C=S).

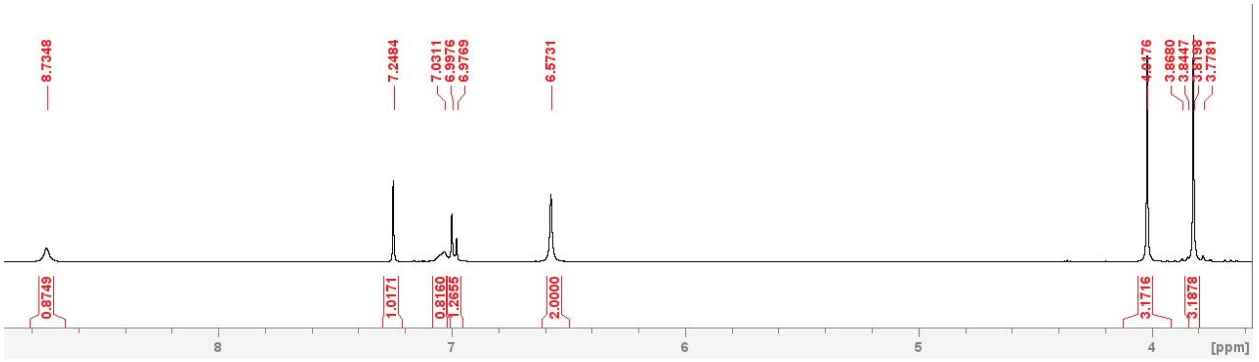

Figure S18: ^{1}H NMR (400 MHz, DMSO-d_6): [ppm] 8.74 (s, 1H, NH_2); 7.26 (s, 1H, CH=N); 7.04 (s, 1H, NH_2); 7.00 (d, 1H, CH_{arom}); 6.59 (s, 2H, CH_{arom}); 4.03 (s, 3H, CH_3); 3.83 (s, 3H, CH_3).

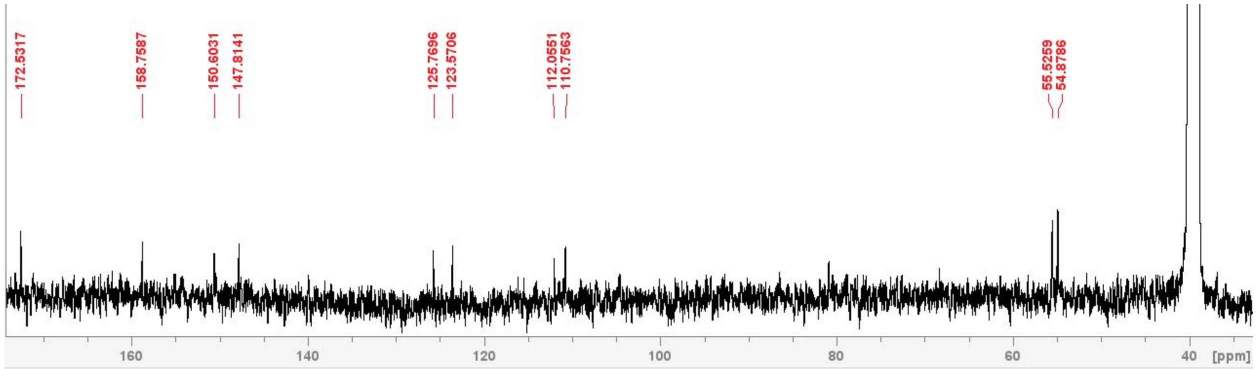

Figure S19: ^{13}C NMR (101 MHz, DMSO-d_6): [ppm] 177.7, 150.7, 149.2, 142.6, 127.0, 122.1, 111.4, 108.7, 55.6.

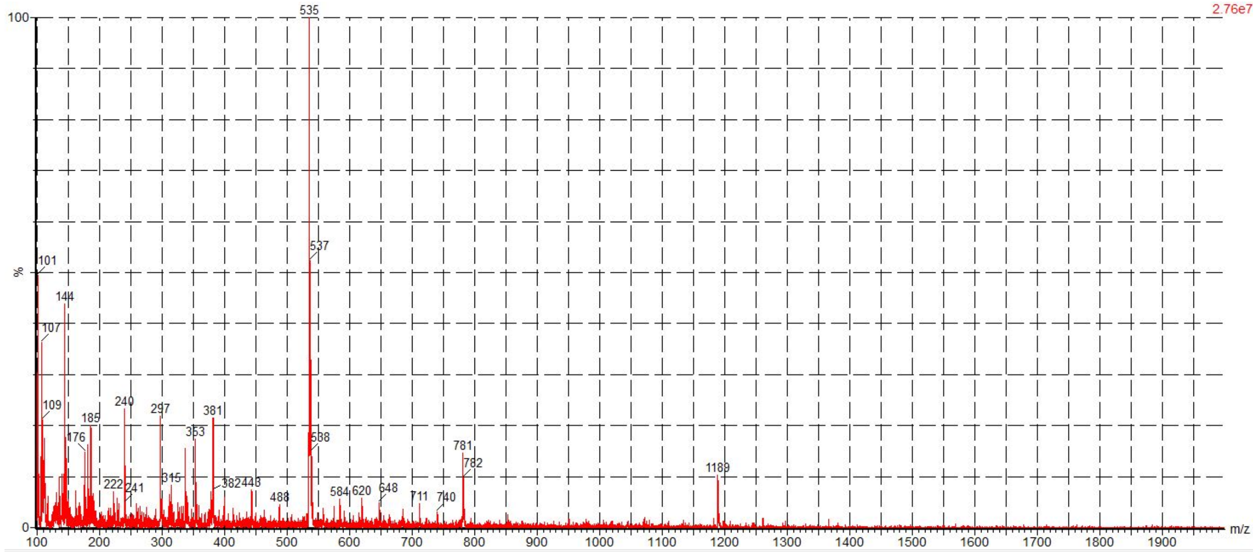

Figure S20: ESI-MS (m/z, %): 535 ([M+H]+, 100).

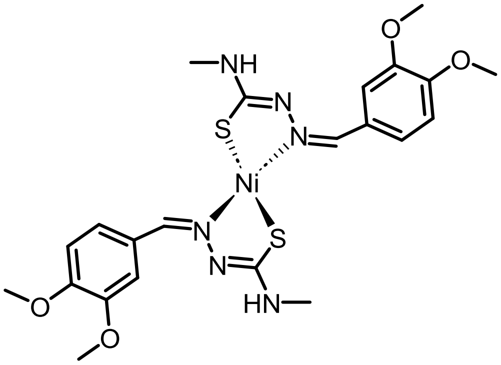

Ni2

$C_{22}H_{32}N_6NiO_4S_2$ 563.32 g/mol

Yield: quant.

Appearance: light brown powder.

Elemental analysis Calcd.: C 46.91%, N 14.92%, H 5.01%, S 11.38% Exp.: C 46.38%, N 14.73%, H 5.07%, S 11.23%. ^{1}H NMR (400 MHz, DMSO-d$_6$): [ppm] 8.74 (s, 1H, N**H**(CH$_3$)); 7.40 (s, 1H, CH=N); 7.16 (s, 1H, CH$_{arom}$); 7.01 (m, 2H, CH$_{arom}$); 3.97 (s, 3H, CH$_3$); 3.84 (s, 3H, CH$_3$); 2.64 (d, 3H, NH(C**H$_3$**)). IR (ATR, cm^{-1}): 3220 w (N-H), 3087, 2958, 2934, 2899, 2834 w (C-H), 1601 m (C=N), 1015, 850 m (C=S). ESI-MS (*m/z*, %): 563 ([M+H]$^+$, 100).

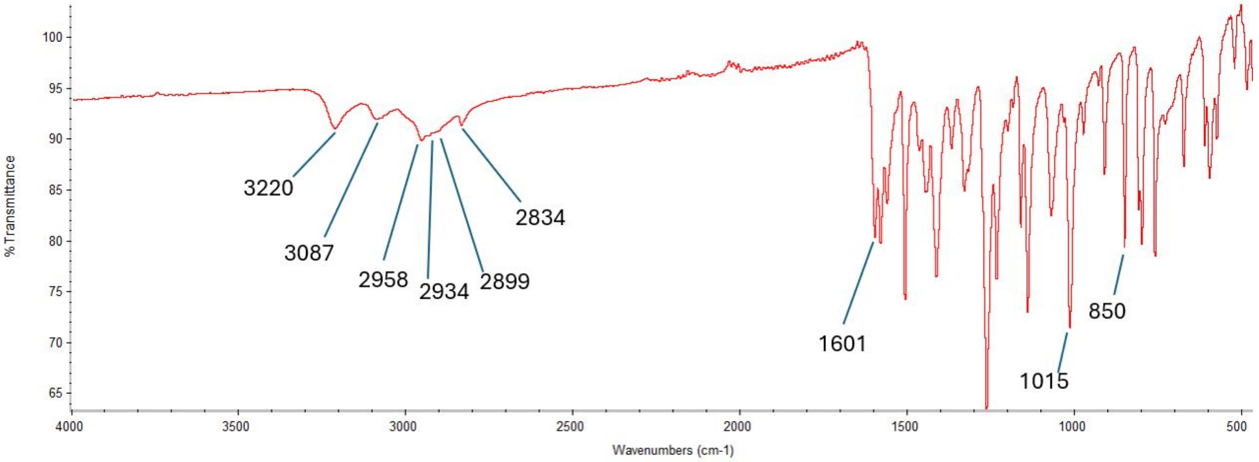

Figure S21: IR (ATR, cm^{-1}): 3220 w (N-H), 3087, 2958, 2934, 2899, 2834w (C-H), 1601 m (C=N), 1015, 850 m (C=S).

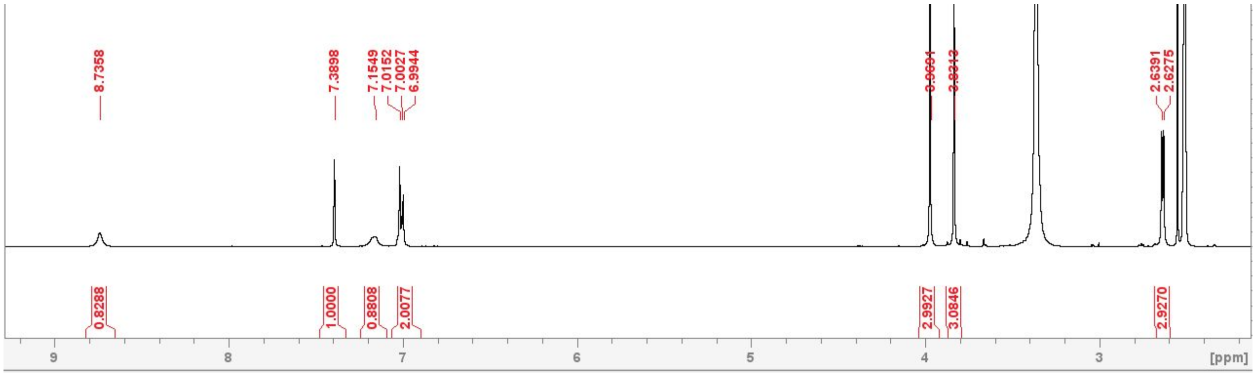

Figure S22: ^{1}H NMR (400 MHz, DMSO-d$_6$): [ppm] 8.74 (s, 1H, N**H**(CH$_3$)); 7.40 (s, 1H, CH=N); 7.16 (s, 1H, CH$_{arom}$); 7.01 (m, 2H, CH$_{arom}$); 3.97 (s, 3H, CH$_3$); 3.84 (s, 3H, CH$_3$); 2.64 (d, 3H, NH(C**H$_3$**)).

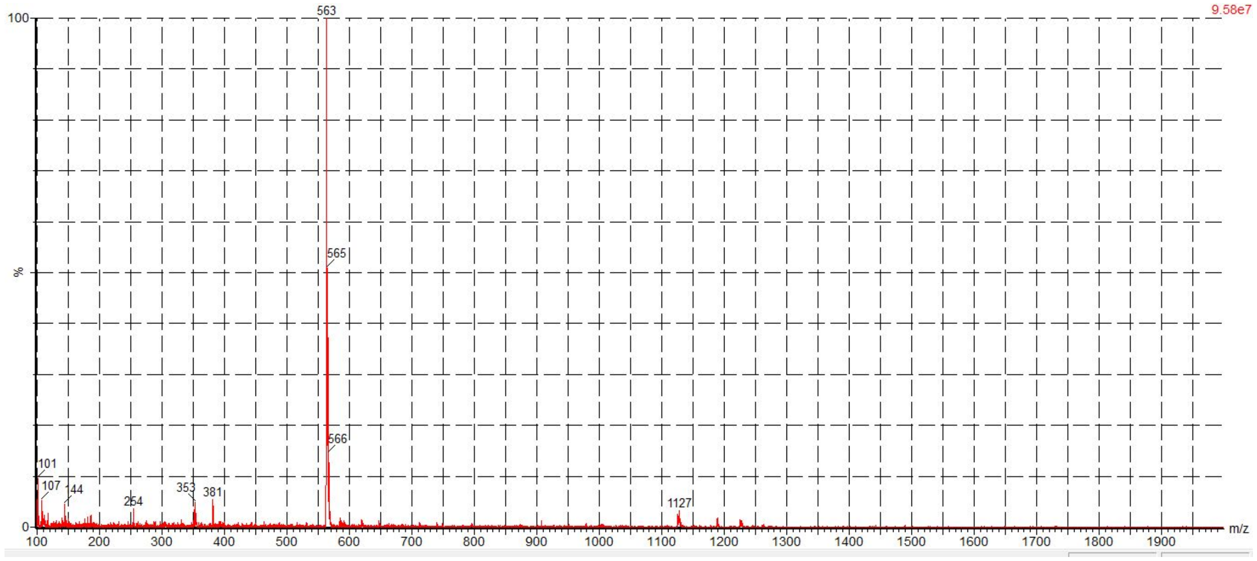

Figure S23: ESI-MS (m/z, %): 563 ($[M+H]^+$, 100).

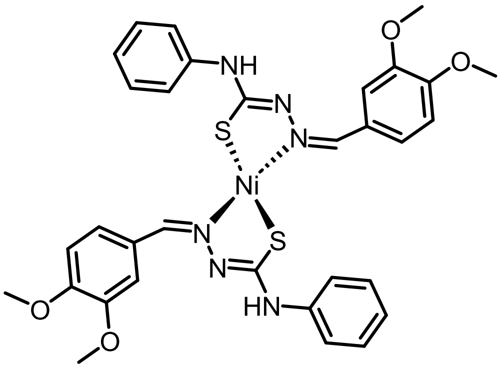

Ni3

$C_{32}H_{38}N_6NiO_7S_2$ 687.46 g/mol

Yield: 40%.

Appearance: light brown solid.

Elemental analysis Calcd.: C 51.83%, N 11.33%, H 5.17%, S 8.65% Exp.: C 51.92%, N 11.56%, H 5.06%, S 8.49. ^1H NMR (400 MHz, DMSO-d_6): [ppm] 9.66 (s, 1H, S=C-NH-Ph); 7.97 (s, 1H,

CH=N); 7.64 (m, 2H, $CH_{arom.}$); 7.58 (m, 2H, $CH_{arom.}$); 7.29 (t, 2H, CH_{arom}); 7.00 (t, 2H, CH_{arom}); 3.81 (s, 3H, CH_3); 3.46 (s, 3H, CH_3). IR (ATR, cm^{-1}): 3294 m (N-H), 3117, 3061, 3032, 3005, 2958, 2926, 2834 w (C-H), 1592 m (C=N), 1021, 833 m (C=S). ESI-MS (*m/z*, %): 688 ($[M+H]^+$, 36).

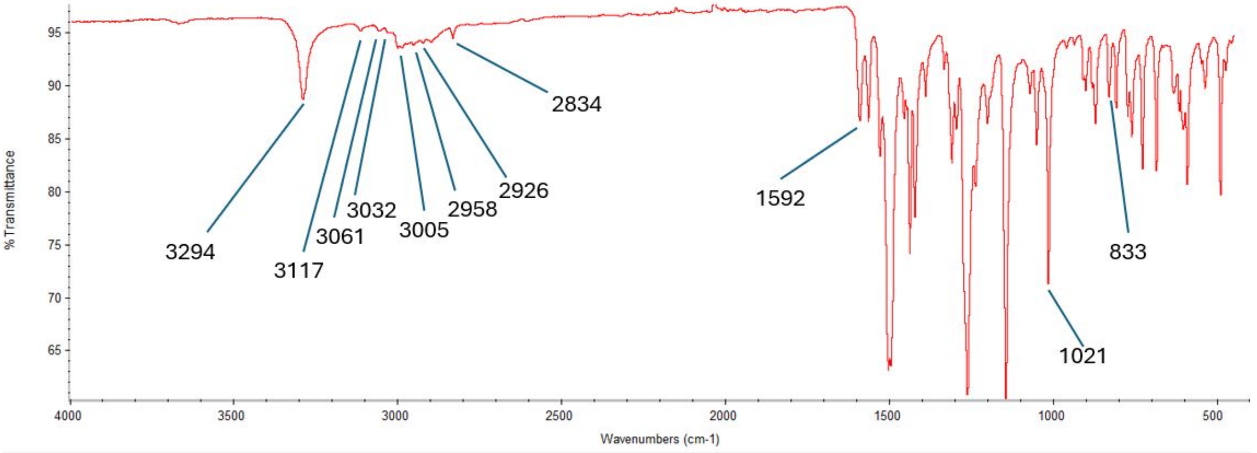

Figure S24: IR (ATR, cm^{-1}): 3294 m (N-H), 3117, 3061, 3032, 3005, 2958, 2926, 2834 w (C-H), 1592 m (C=N), 1021, 833 m (C=S).

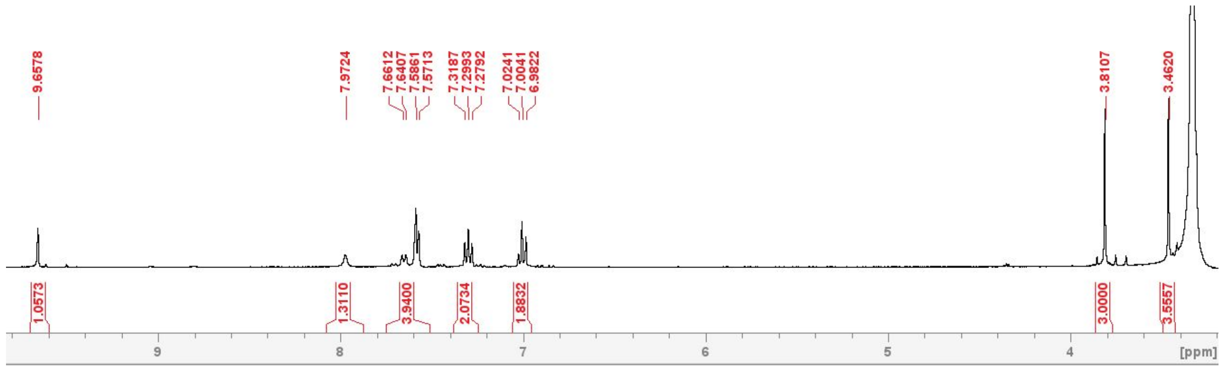

Figure S25: ^{1}H NMR (400 MHz, DMSO-d_6): [ppm] 9.66 (s, 1H, S=C-NH-Ph); 7.97 (s, 1H, HC=N); 7.64 (m, 2H, $CH_{arom.}$); 7.58 (m, 2H, $CH_{arom.}$); 7.29 (t, 2H, CH_{arom}); 7.00 (t, 2H, CH_{arom}); 3.81 (s, 3H, CH_3); 3.46 (s, 3H, CH_3).

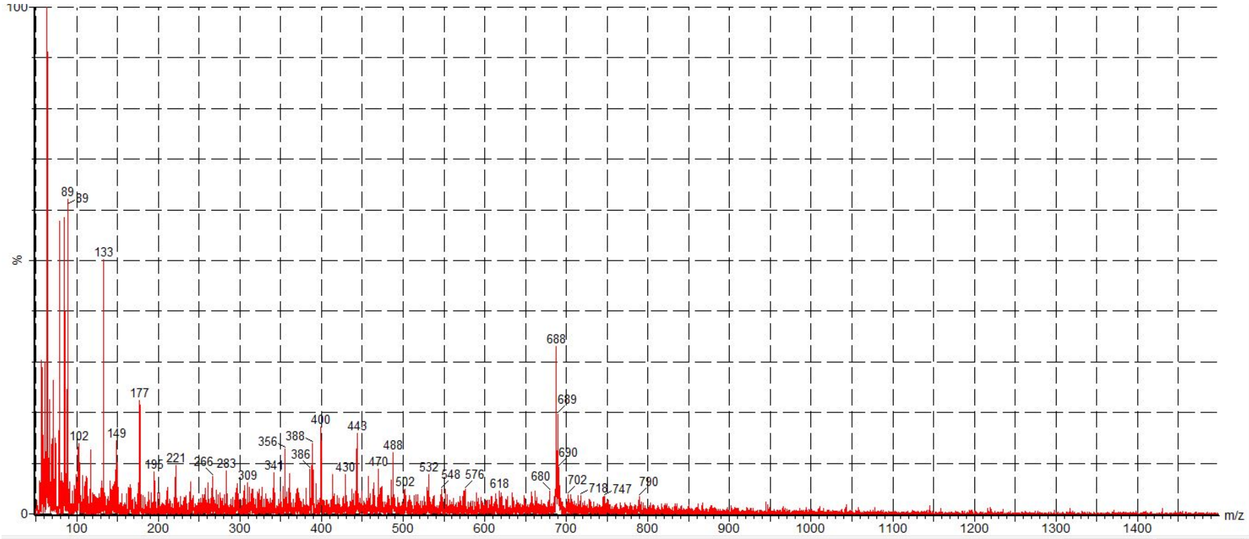

Figure S26: ESI-MS (m/z, %): 688 ($[M+H]^+$, 36).

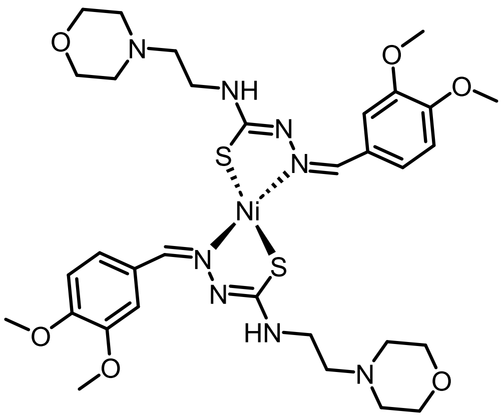

Ni4

$C_{32}H_{50}N_8NiO_6S_2$ 761.58 g/mol

Yield: 58%.

Appearance: light brown powder.

Elemental Analysis Calcd.: C 50.47%, N 14.71%, H 6.09%, S 8.42% Exp.: C 50.23%, N 14.55%, H 6.11%, S 8.29%. ^1H NMR (400 MHz, DMSO-d_6): [ppm] 8.12 (s, 1H, S=C-NH-R); 7.48 (d, 1H, $CH_{arom.}$); 7.34 (m, 1H, $CH_{arom.}$); 7.31 (s, 1H, CH=N); 6.97 (d, 1H, $CH_{arom.}$); 3.80 (s, 3H, CH_3); 3.76 (s, 3H, CH_3); 3.56 (m, 6H, S=C-NH-C$\mathbf{H_2}$- and -CH_2-O-CH_2); 2.47 (m, 2H, S=C-

NH-CH_2-C$\mathbf{H_2}$-N); 2.37 (m, 4H, -CH_2-N-CH_2). IR (ATR, cm^{-1}): 3338 w, (N-H), 3123, 3002, 2973, 2940, 2864, 2840, 2802, 2767 m (C-H), 1592 m (C=N), 1036, 815 m (C=S). ESI-MS (*m/z*, %): 762 ($[M+H]^+$, 92).

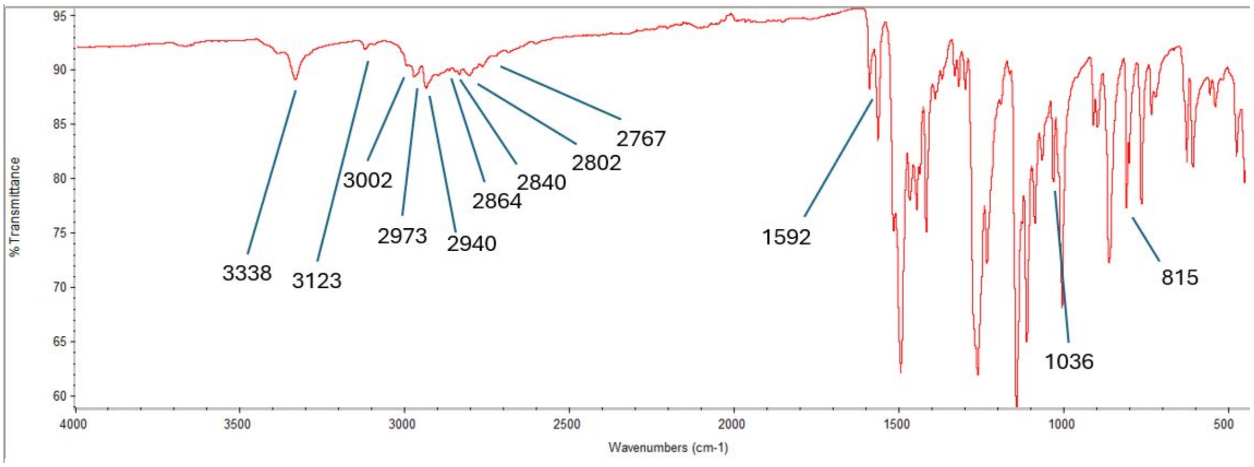

Figure S27: IR (ATR, cm^{-1}): 3338 w, (N-H), 3123, 3002, 2973, 2940, 2864, 2840, 2802, 2767 m (C-H), 1592 m (C=N), 1036, 815 m (C=S).

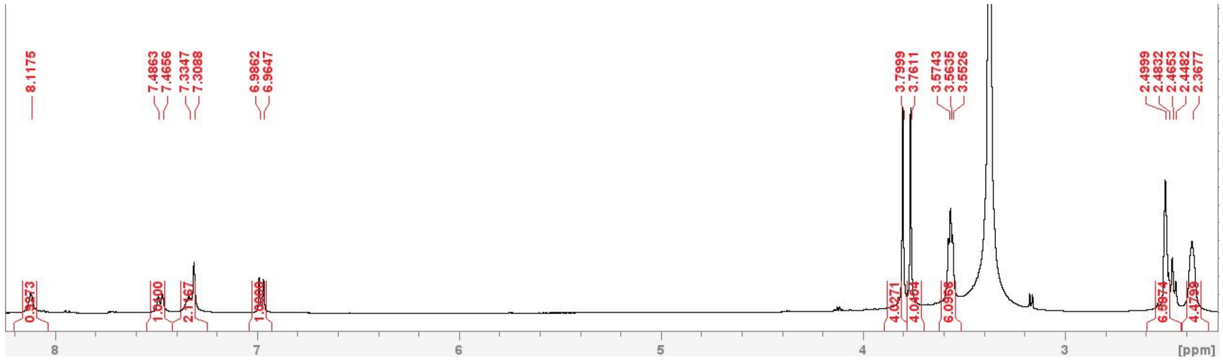

Figure S28: ^{1}H NMR (400 MHz, DMSO-d_6): [ppm] 8.12 (s, 1H, S=C-NH-R); 7.48 (d, 1H, $CH_{arom.}$); 7.34 (m, 1H, $CH_{arom.}$); 7.31 (s, 1H, CH=N); 6.97 (d, 1H, $CH_{arom.}$); 3.80 (s, 3H, CH_3); 3.76 (s, 3H, CH_3); 3.56 (m, 6H, S=C-NH-C$\mathbf{H_2}$- and -CH_2-O-CH_2); 2.47 (m, 2H, S=C-NH-CH_2-C$\mathbf{H_2}$-N); 2.37 (m, 4H, -CH_2-N-CH_2).

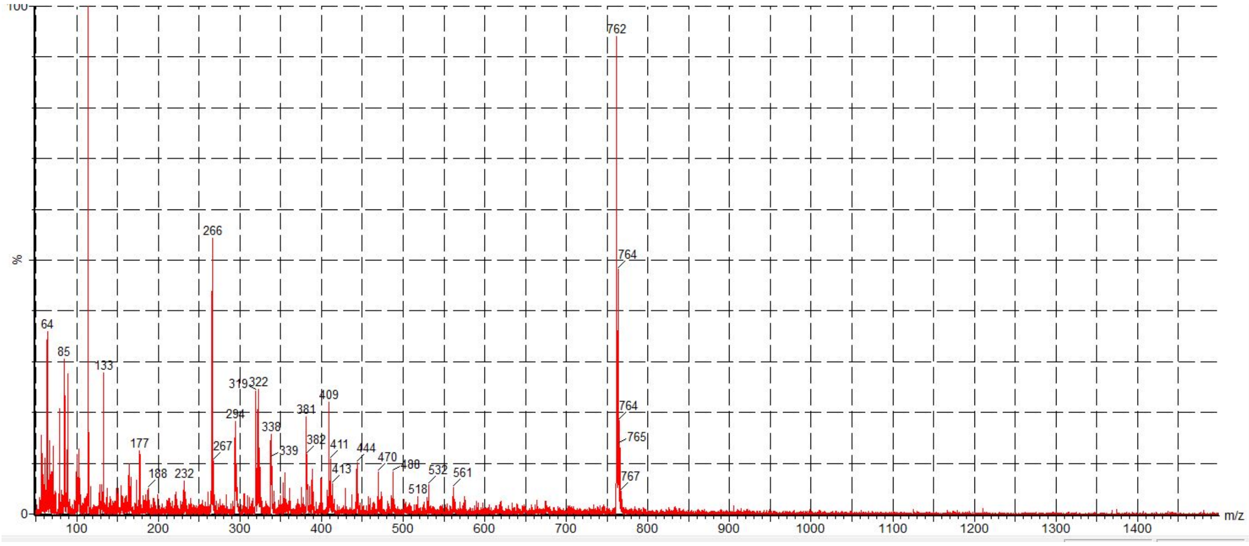

Figure S29: ESI-MS (m/z, %): 762 ($[M+H]^+$, 92).

2.3 X-ray Diffraction Characterization (L4, Ni1, Ni2, Ni3, Ni4)

```
Ring puckering coordinates
following Cremer D. & Pople J.A., JACS (1975).97,1354

Ring  1
Atom            Internal cartesian coordinates
               X                Y                 Z
Ni1      0.0000(0.0001)  1.7073(0.0025) -0.1795(0.0034)
S1       1.5443(0.0026)  0.2159(0.0038)  0.1437(0.0037)
C1       0.4960(0.0059) -1.1486(0.0070) -0.0530(0.0046)
N2      -0.8134(0.0057) -1.0460(0.0055) -0.0579(0.0045)
N1      -1.2269(0.0058)  0.2714(0.0051)  0.1467(0.0041)
q2   =   0.2838(0.0058)
phi2  =   179.18(  1.49)

Asymmetry parameters
Following Nardelli M., Acta Cryst.(1983). C39, 1141

Ni1   S1    C1    N2    N1

DS(Ni1    )=0.0171(0.0029)        D2(Ni1    )=0.1432(0.0023)
DS(S1     )=0.1211(0.0033)        D2(S1     )=0.1084(0.0023)
DS(C1     )=0.1830(0.0028)        D2(C1     )=0.0376(0.0023)
DS(N2     )=0.1783(0.0028)        D2(N2     )=0.0500(0.0026)
DS(N1     )=0.1042(0.0031)        D2(N1     )=0.1123(0.0022)
```

Q2= 0.2838

Phi2 = 179.18

Ni1

```
Ring puckering coordinates
following Cremer D. & Pople J.A., JACS (1975).97,1354

Ring  1
Atom            Internal cartesian coordinates
               X                Y                 Z
Ni1      0.0000(0.0000)  1.7122(0.0008)  0.1637(0.0008)
S1       1.5495(0.0006)  0.2200(0.0007) -0.1323(0.0008)
C1       0.4906(0.0018) -1.1499(0.0017)  0.0503(0.0010)
N2      -0.8073(0.0012) -1.0526(0.0014)  0.0508(0.0010)
N1      -1.2328(0.0011)  0.2704(0.0013) -0.1326(0.0008)
q2   =   0.2588(0.0012)
phi2  =    -0.09(  0.34)

Asymmetry parameters
Following Nardelli M., Acta Cryst.(1983). C39, 1141

Ni1   S1    C1    N2    N1

DS(Ni1    )=0.0144(0.0006)        D2(Ni1    )=0.1302(0.0005)
DS(S1     )=0.1082(0.0007)        D2(S1     )=0.0997(0.0005)
DS(C1     )=0.1656(0.0006)        D2(C1     )=0.0357(0.0005)
DS(N2     )=0.1630(0.0006)        D2(N2     )=0.0436(0.0006)
DS(N1     )=0.0968(0.0007)        D2(N1     )=0.1011(0.0005)
```

Q2= 0.2588

Phi2 = -0.09

Ni3

```
Ring puckering coordinates
following Cremer D. & Pople J.A., JACS (1975).97,1354

Ring  1
Atom            Internal cartesian coordinates
              X              Y              Z
Ni1      0.0000(0.0000)  1.7276(0.0010) -0.0608(0.0012)
S1A      1.5644(0.0007)  0.2120(0.0012)  0.0526(0.0012)
C1A      0.4889(0.0017) -1.1531(0.0018) -0.0243(0.0013)
N2A     -0.8005(0.0016) -1.0514(0.0016) -0.0132(0.0014)
N1A     -1.2528(0.0017)  0.2648(0.0016)  0.0457(0.0013)
q2  =  0.0965(0.0015)
phi2  = -174.50(  1.07)

Asymmetry parameters
Following Nardelli M., Acta Cryst.(1983). C39, 1141

Ni1   S1A   C1A   N2A   N1A

DS(Ni1   )=0.0069(0.0010)        D2(Ni1   )=0.0478(0.0007)
DS(S1A   )=0.0342(0.0010)        D2(S1A   )=0.0398(0.0007)
DS(C1A   )=0.0587(0.0009)        D2(C1A   )=0.0175(0.0007)
DS(N2A   )=0.0621(0.0009)        D2(N2A   )=0.0111(0.0008)
DS(N1A   )=0.0413(0.0009)        D2(N1A   )=0.0342(0.0007)
```

Q2= 0.0965

Phi2 = -174.50

```
Ring  2
Atom            Internal cartesian coordinates
              X              Y              Z
Ni1      0.0000(0.0000)  1.7263(0.0011) -0.0662(0.0009)
S1B      1.5505(0.0009)  0.2209(0.0014)  0.0504(0.0012)
C1B      0.4930(0.0017) -1.1546(0.0016) -0.0154(0.0013)
N2B     -0.8013(0.0016) -1.0555(0.0017) -0.0255(0.0013)
N1B     -1.2423(0.0017)  0.2629(0.0020)  0.0566(0.0014)
q2  =  0.1050(0.0016)
phi2  =  175.44(  1.09)

Asymmetry parameters
Following Nardelli M., Acta Cryst.(1983). C39, 1141

Ni1   S1B   C1B   N2B   N1B

DS(Ni1   )=0.0100(0.0010)        D2(Ni1   )=0.0528(0.0007)
DS(S1B   )=0.0487(0.0010)        D2(S1B   )=0.0376(0.0007)
DS(C1B   )=0.0690(0.0009)        D2(C1B   )=0.0109(0.0007)
DS(N2B   )=0.0640(0.0009)        D2(N2B   )=0.0220(0.0008)
DS(N1B   )=0.0342(0.0009)        D2(N1B   )=0.0436(0.0006)
```

Q2= 0.1054

Phi2 = 175.44

Ni2

```
Ring puckering coordinates
following Cremer D. & Pople J.A., JACS (1975).97,1354

Ring  1
Atom            Internal cartesian coordinates
              X              Y              Z
Ni1      0.0000(0.0000)  1.7267(0.0000) -0.1276(0.0000)
S1A      1.5532(0.0000)  0.2095(0.0000)  0.0952(0.0000)
C1A      0.4803(0.0000) -1.1514(0.0000) -0.0265(0.0000)
N2A     -0.8130(0.0000) -1.0584(0.0000) -0.0524(0.0000)
N1A     -1.2205(0.0000)  0.2736(0.0000)  0.1112(0.0000)
q2  =  0.2028(0.0001)
phi2  =  173.91(  0.01)

Asymmetry parameters
Following Nardelli M., Acta Cryst.(1983). C39, 1141

Ni1   S1A   C1A   N2A   N1A

DS(Ni1   )=0.0235(0.0000)        D2(Ni1   )=0.1022(0.0000)
DS(S1A   )=0.0976(0.0000)        D2(S1A   )=0.0710(0.0000)
DS(C1A   )=0.1347(0.0000)        D2(C1A   )=0.0194(0.0000)
DS(N2A   )=0.1227(0.0000)        D2(N2A   )=0.0456(0.0000)
DS(N1A   )=0.0628(0.0000)        D2(N1A   )=0.0859(0.0000)
```

Q2= 0.2028

Phi2 = 173.91

```
Ring  2
Atom            Internal cartesian coordinates
              X              Y              Z
Ni1      0.0000(0.0000)  1.7289(0.0000)  0.1309(0.0000)
S1B      1.5424(0.0000)  0.2079(0.0000) -0.1026(0.0000)
C1B      0.4956(0.0000) -1.1595(0.0000)  0.0351(0.0000)
N2B     -0.8042(0.0000) -1.0407(0.0000)  0.0458(0.0000)
N1B     -1.2338(0.0000)  0.2633(0.0000) -0.1092(0.0000)
q2  =  0.2071(0.0000)
phi2  =   -2.46(  0.00)

Asymmetry parameters
Following Nardelli M., Acta Cryst.(1983). C39, 1141

Ni1   S1B   C1B   N2B   N1B

DS(Ni1   )=0.0153(0.0000)        D2(Ni1   )=0.1044(0.0000)
DS(S1B   )=0.0919(0.0000)        D2(S1B   )=0.0767(0.0000)
DS(C1B   )=0.1346(0.0000)        D2(C1B   )=0.0248(0.0000)
DS(N2B   )=0.1283(0.0000)        D2(N2B   )=0.0396(0.0000)
DS(N1B   )=0.0722(0.0000)        D2(N1B   )=0.0837(0.0000)
```

Q2= 0.2071

Phi2 = -2.46

Ni4

Figure S30. Asimmetry parameters to evaluate the distorsion of the chelation rings.

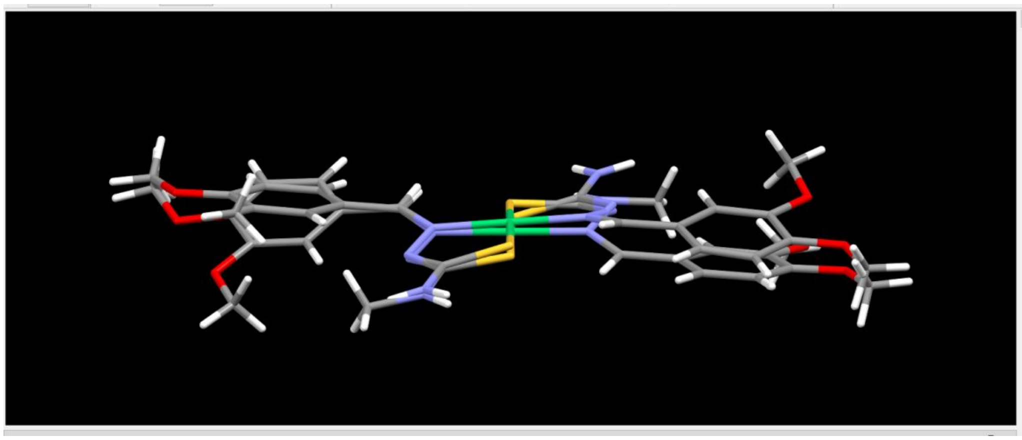

Ni1 and Ni2

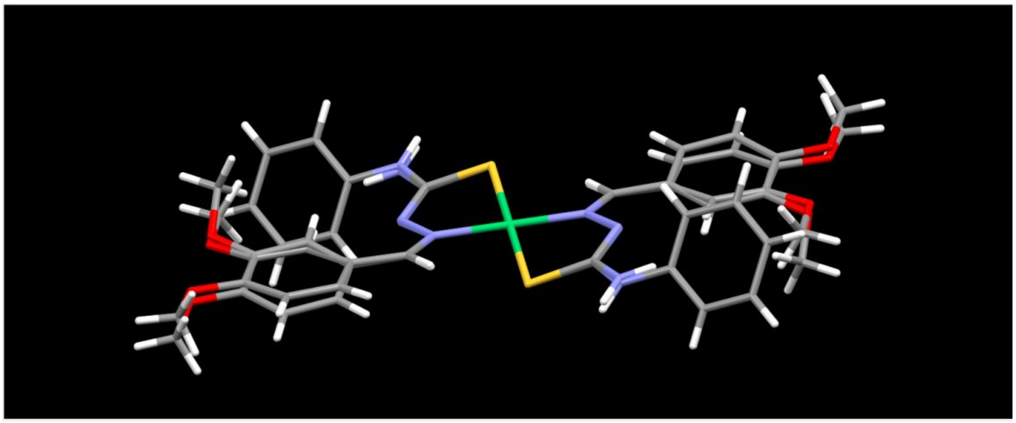

Ni1 and Ni3

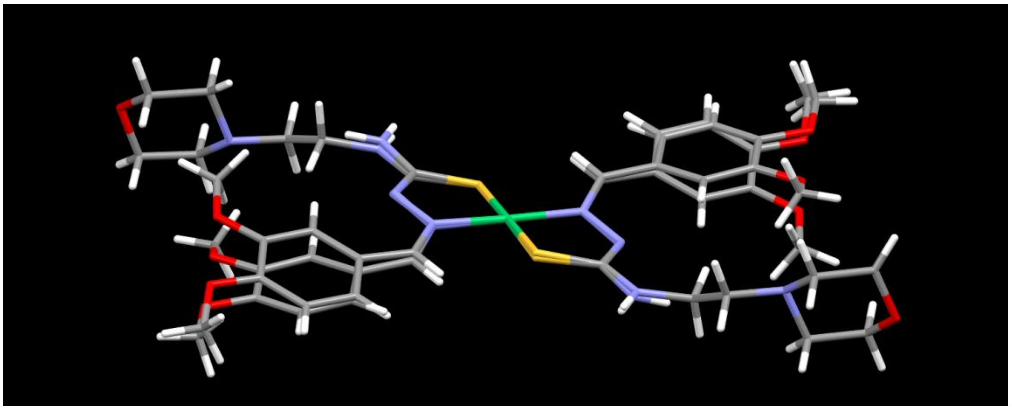

Ni1 and Ni4

Figure S31. Comparison of the coordination systems evidencing the distortions of the chelating systems.

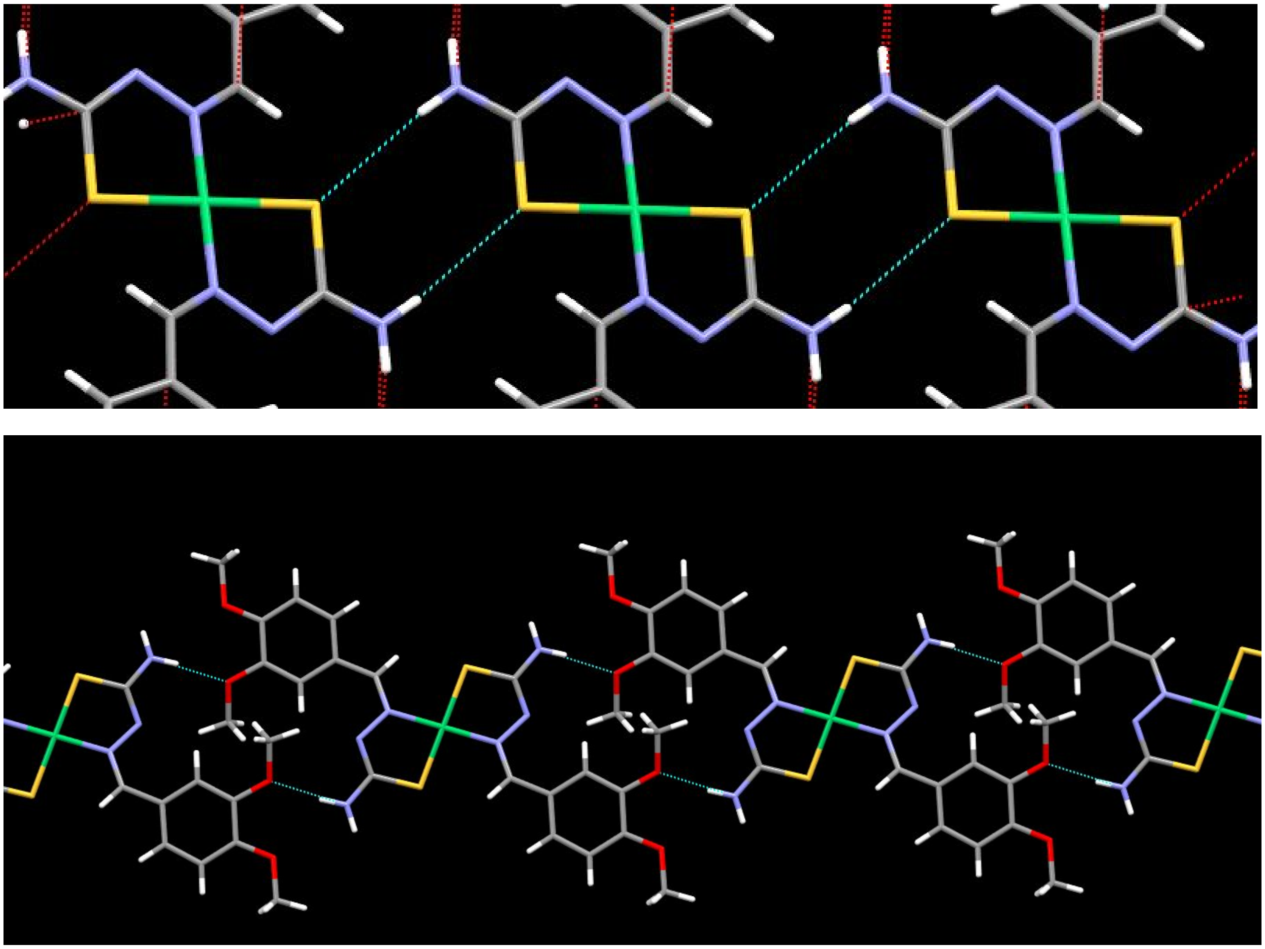

Ni1

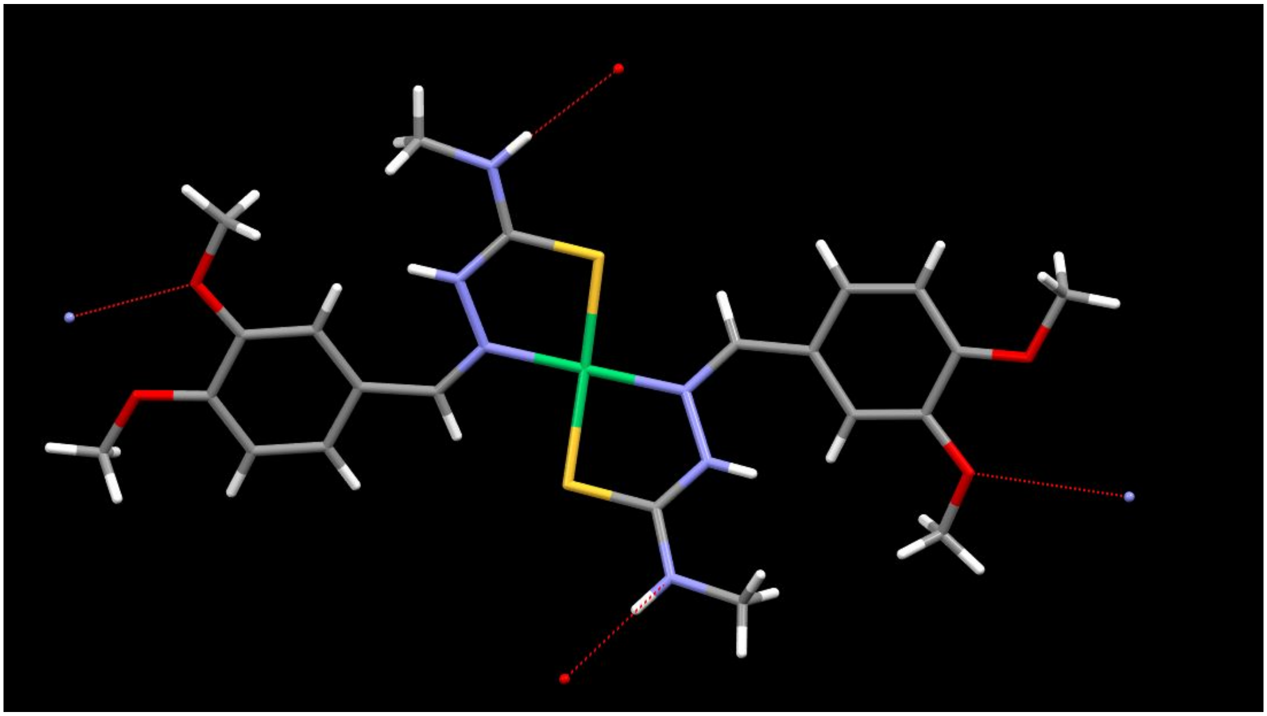

Ni2

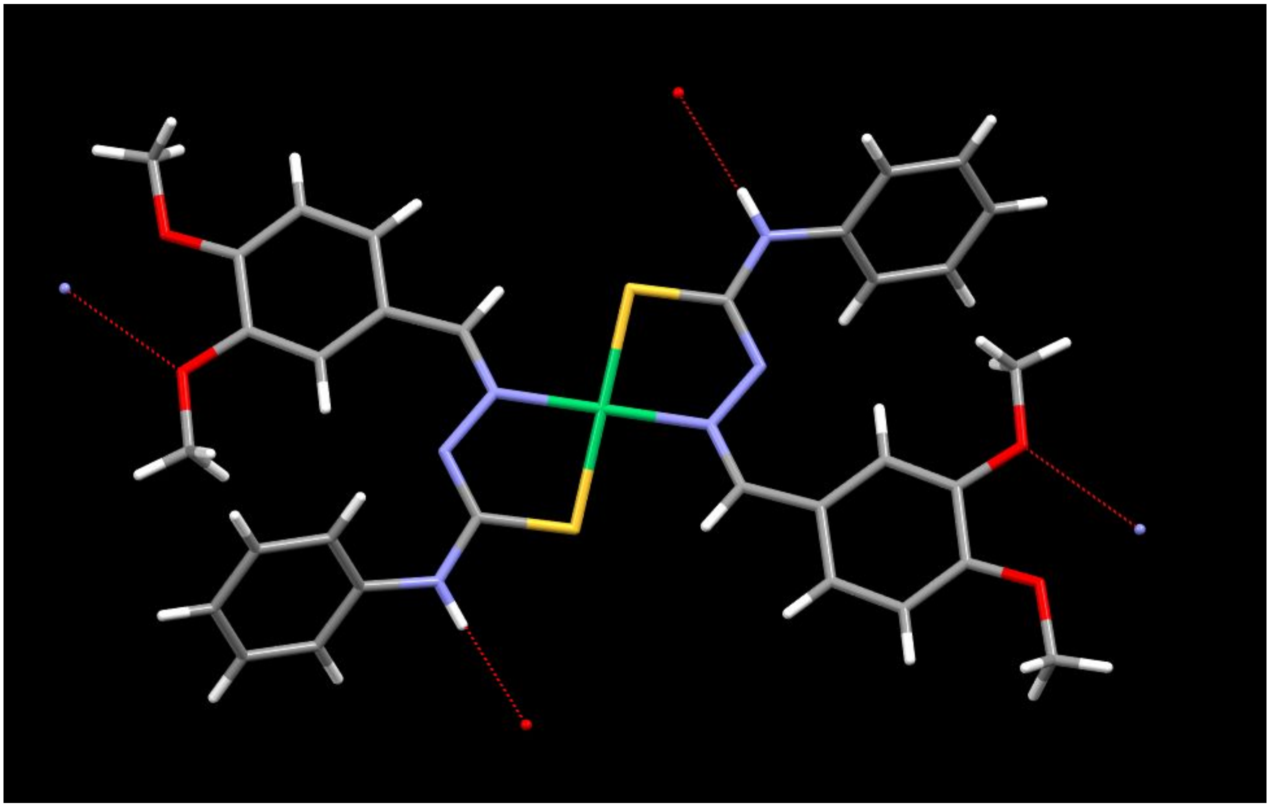

Ni3

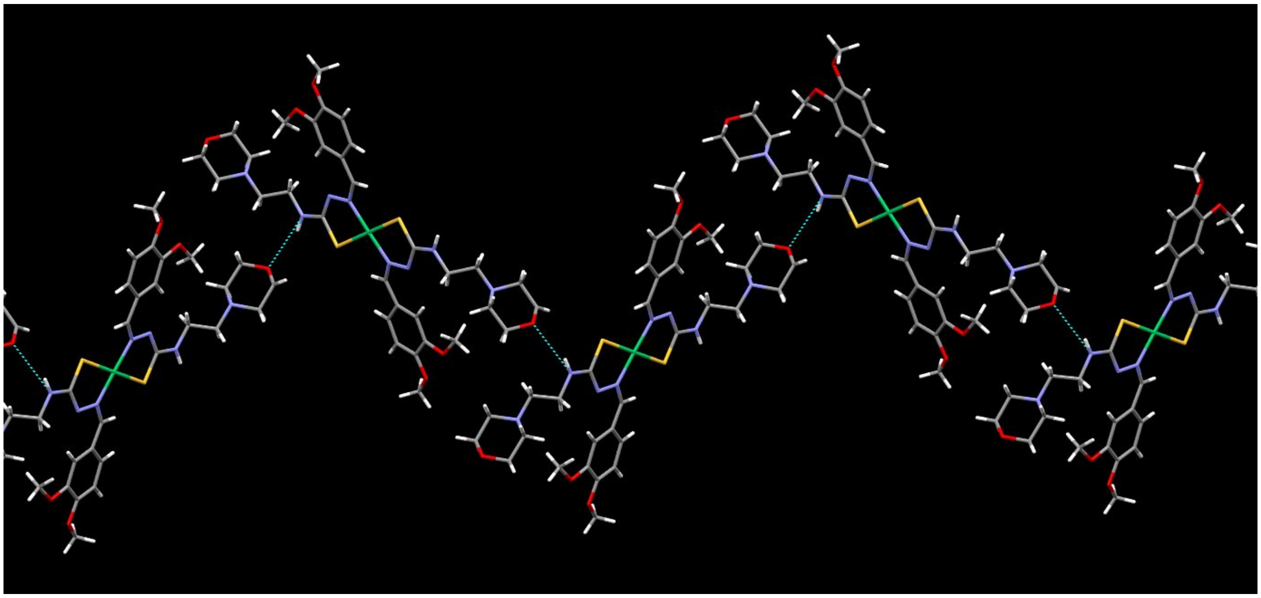

Ni4

Figure S32. Contacts and hydrogen bonds in the crystal structures of the molecules.

2.4 Electronic Spectroscopy

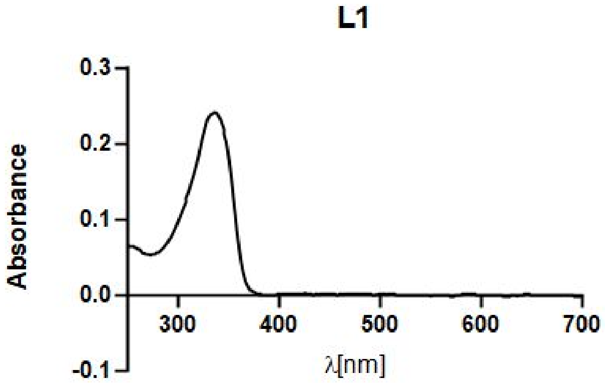

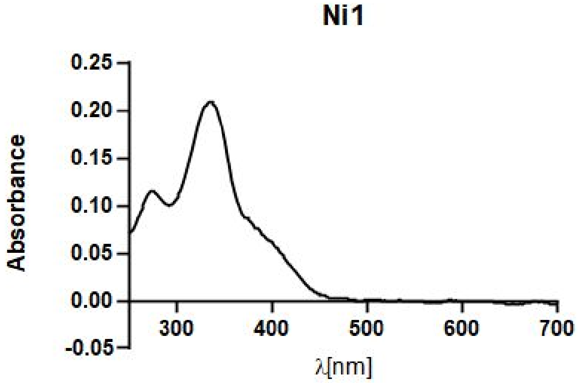

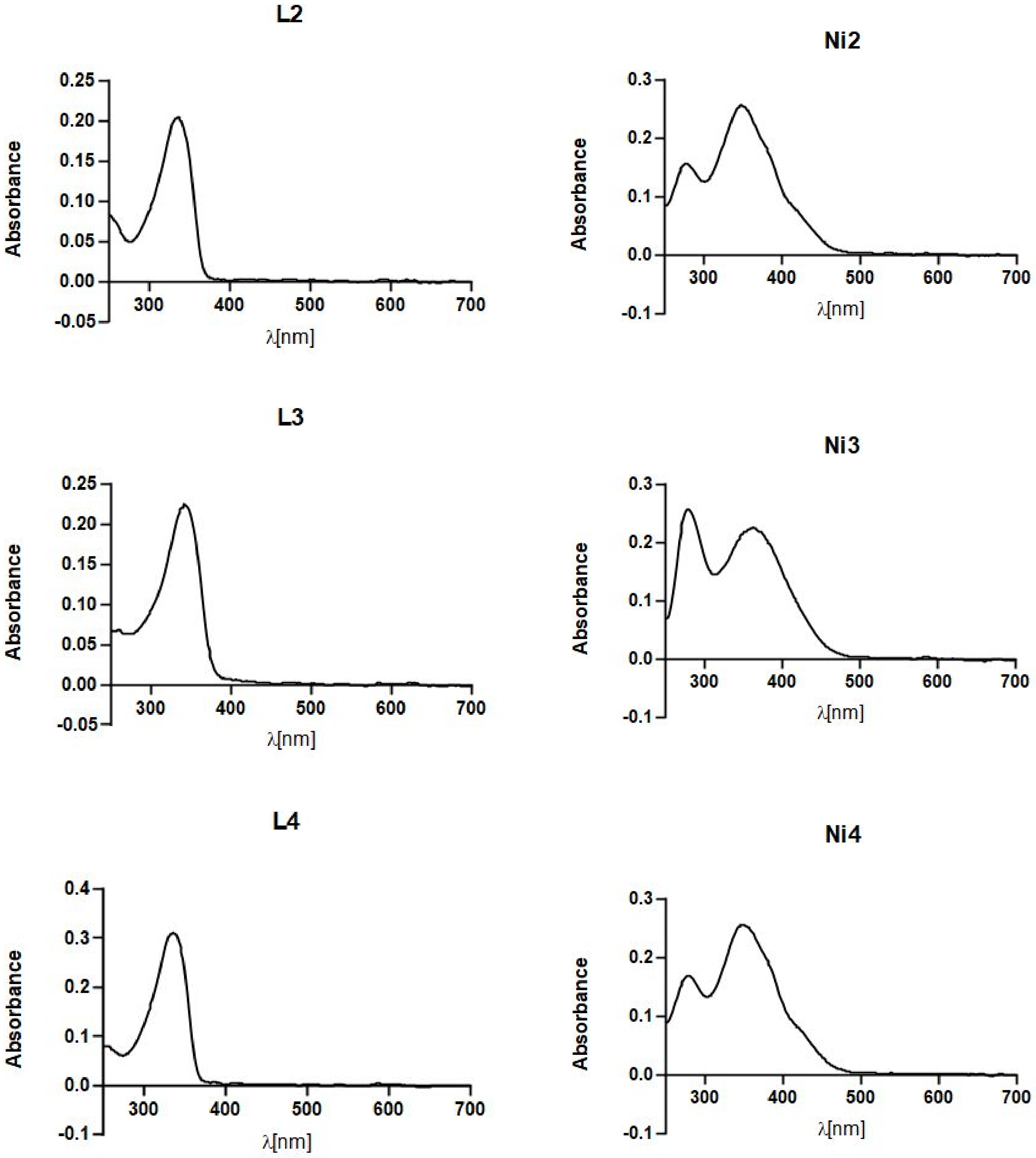

Figure S33: Chart of the electronic spectra of the compounds in DMSO at 7 μM.

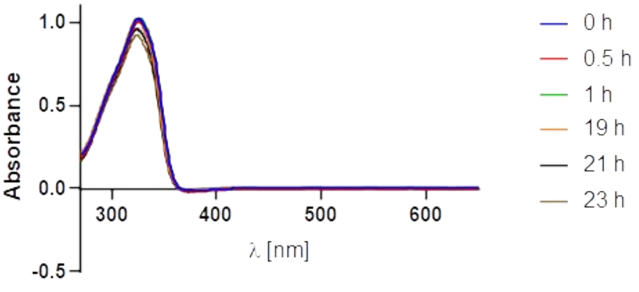

L1

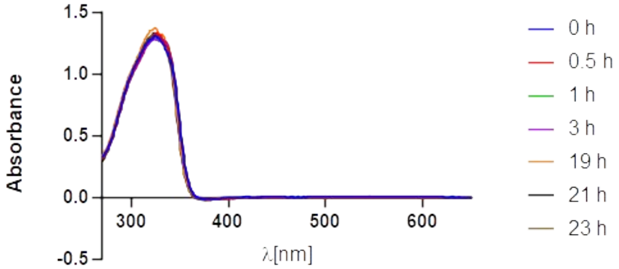

Ni1

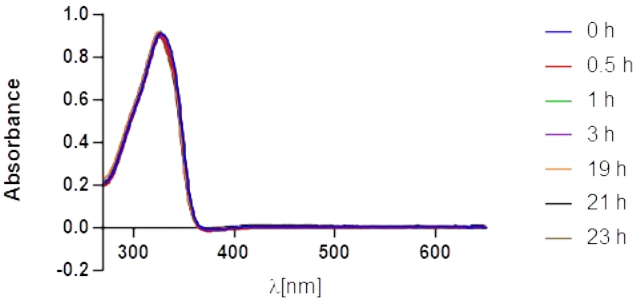

L2

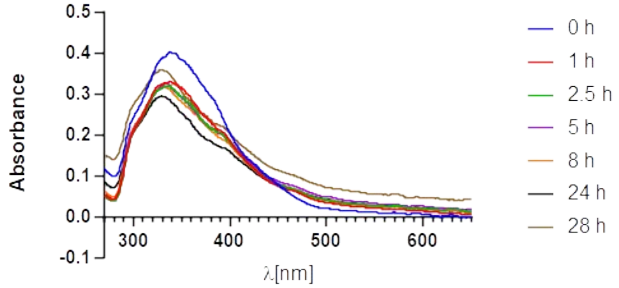

Ni2

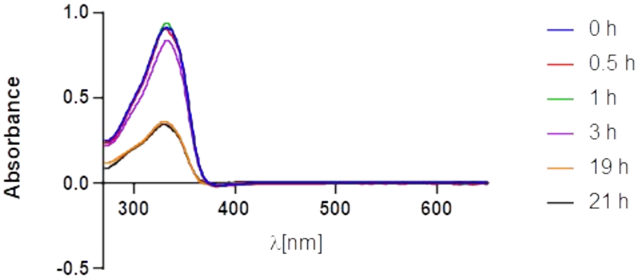

L3

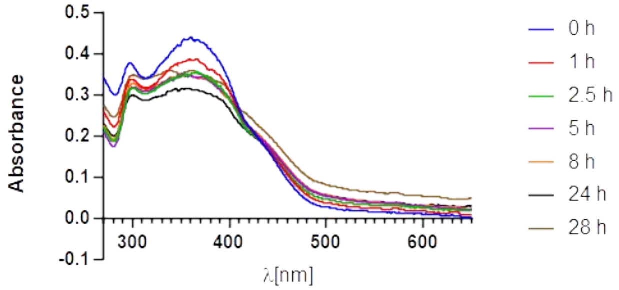

Ni3

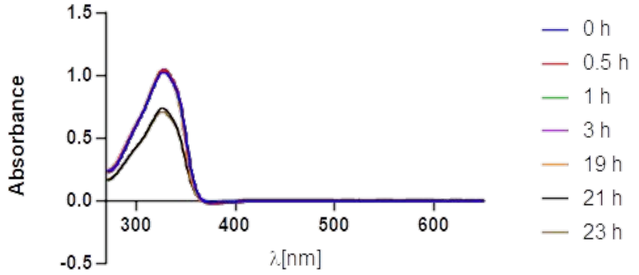

L4

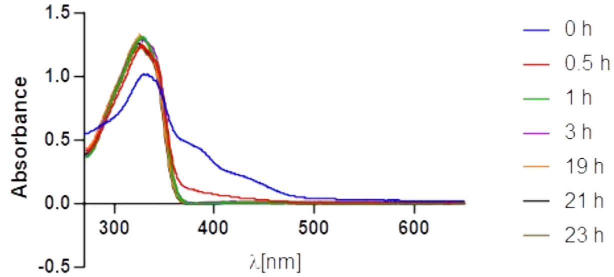

Ni4

Figure S34: Chart of the stability assays in aqueous medium. The compounds were dissolved in PBS buffer (pH 7.4,

[PBS]=10 mM, [NaCl]=137 mM, [KCl]=2.7 mM) containing 5% DMSO, and their UV-visible spectra were recorded in a 700 µL quartz cuvette with a path length of 1 cm. The compounds were incubated for 24 hours in a standardized chamber at a temperature fixed at 37 °C and atmospheric pressure.

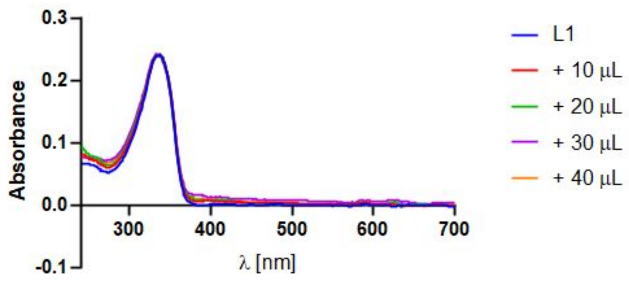

L1

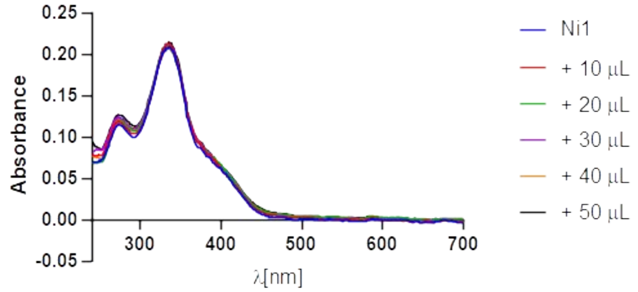

Ni1

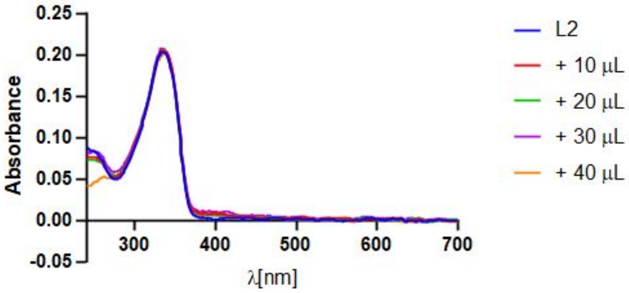

L2

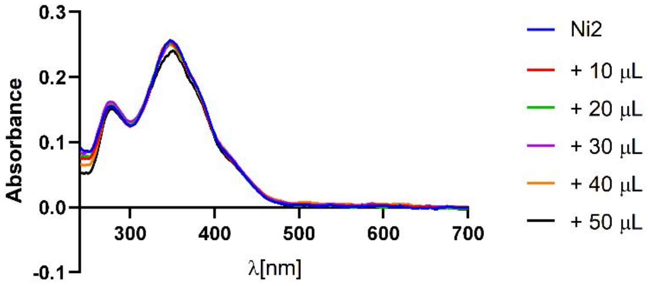

Ni2

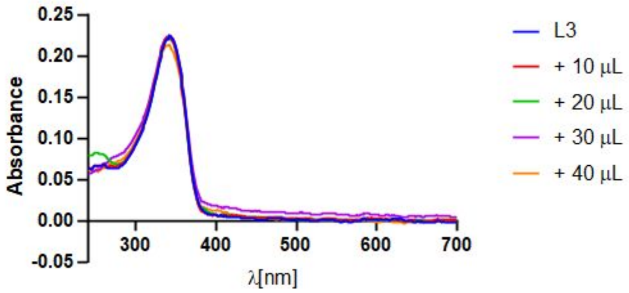

L3

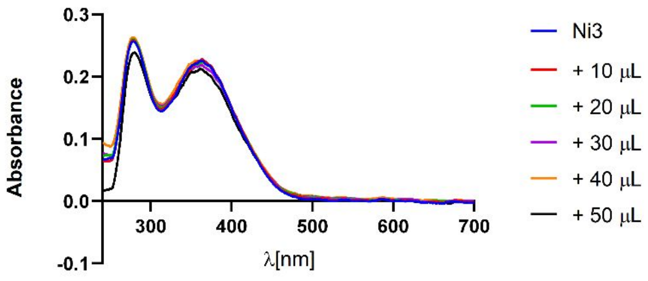

Ni3

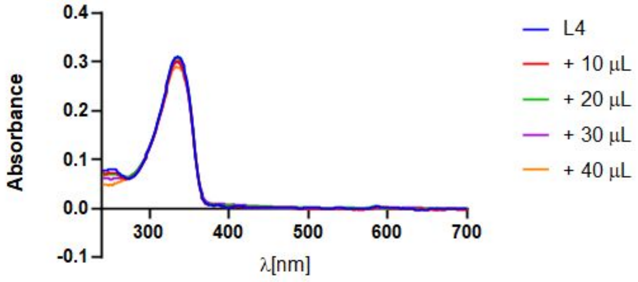

L4

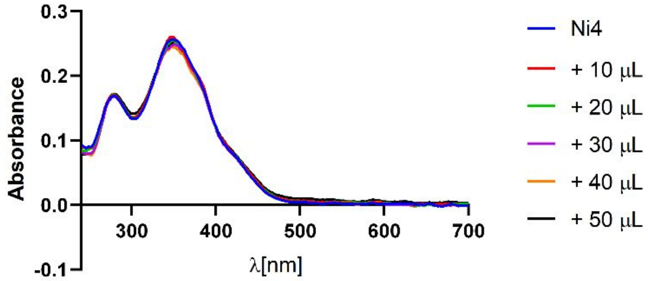

Ni4

Figure S35: Incremental additions of KO_2 solution in dry DMSO (14 µM) to compounds' solutions (600 µL, 7 µM) at the UV-visible spectrophotometer showed inactivity of ligands and complexes to reduction due to superoxide anion.

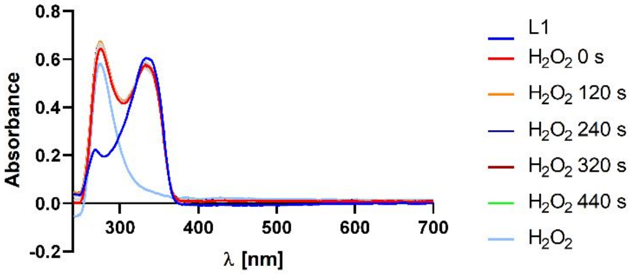

L1

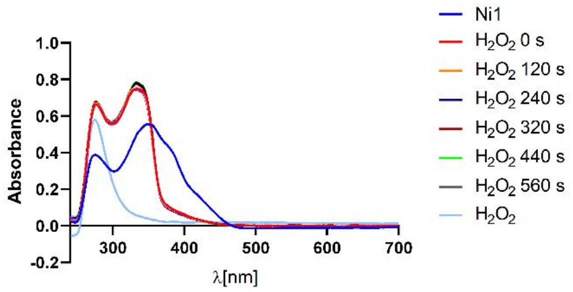

Ni1

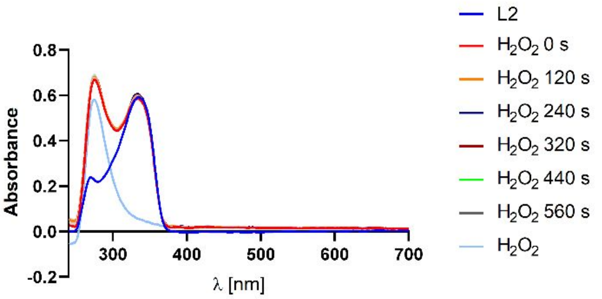

L2

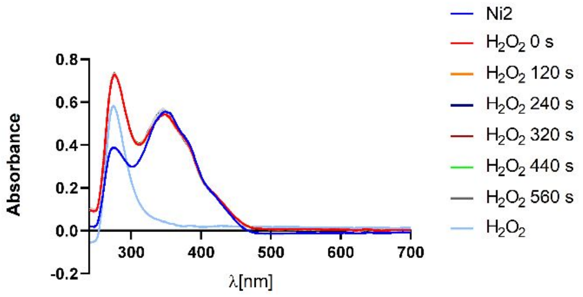

Ni2

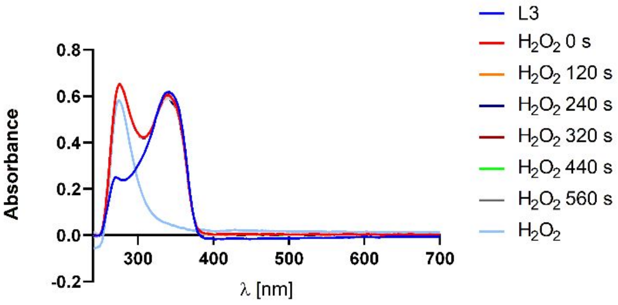

L3

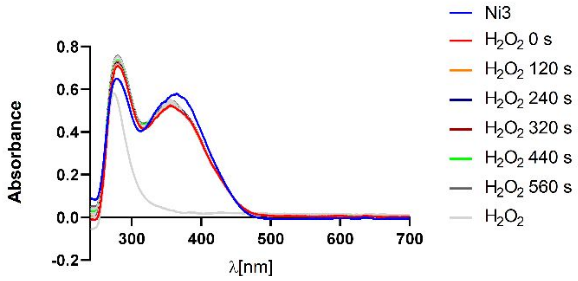

Ni3

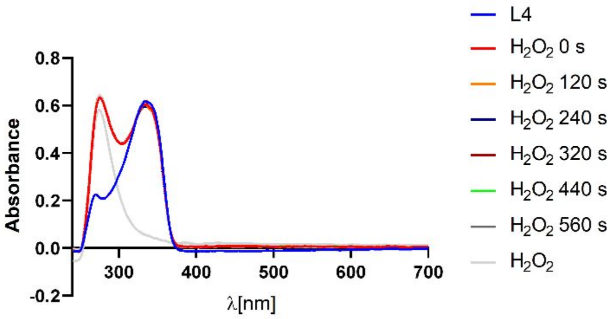

L4

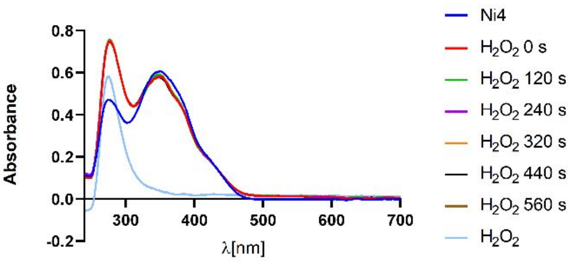

Ni4

Figure S36: H_2O_2 assay. Solutions of complexes were prepared in DMSO, while H_2O_2 was diluted in distilled water to obtain a 1 M

stock solution. 500 μL of 10 μM compound solution in DMSO was registered before and after addition of 100 μL of water or H_2O_2 at 1 M. UV-Visible spectra were registered in the 250-750 nm window every 12 seconds for 5 minutes.

2.5 Cyclic Voltammetry Characterization

Table S1: Peak potentials and currents of the complexes **Ni1-4**, shown in **Chart 2**. We add here the calculated potential values referenced to Ag-AgCl and to SHE electrodes, in order to compare the values to other values from literature.

Complex	Peak Label	Peak potential vs pseudoreference (V)	Peak potential vs Ag_AgCl 3M (V)	Peak potential vs Standard Hydrogen Electrode (SHE) (V)	Peak Current (μA)
**Ni1**	1	-1.16	-1.26	-1.063	28.6
	2	-0.47	-0.57	-0.373	31.7
	3	-1.08	-1.18	-0.983	43.8
**Ni2**	1	-1.31	-1.41	-1.213	14.6
	2	-0.60	-0.70	-0.503	52.7
	3	-1.20	-1.30	-1.103	43.6
	4	-1.69	-1.79	-1.593	18.0
**Ni3**	1	-0.60	-0.70	-0.503	95.7
	2	-1.27	-1.37	-1.173	22.0
**Ni4**	1	-1.16	-1.26	-1.063	14.1
	2	-0.55	-0.65	-0.453	46.6
	3	-1.13	-1.23	-1.033	42.8
	4	-1.62	-1.72	-1.523	36.9

2.6 Computational Study

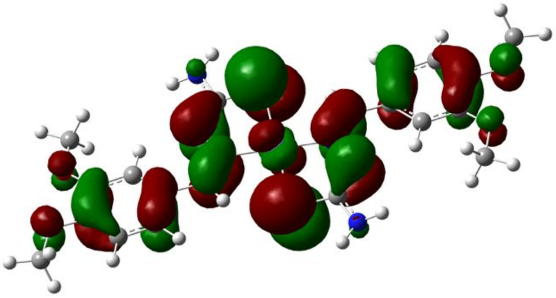

Ni1 HOMO

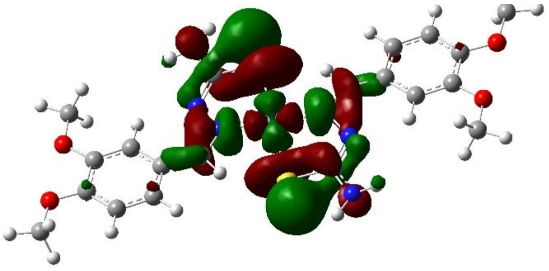

Ni1 LUMO

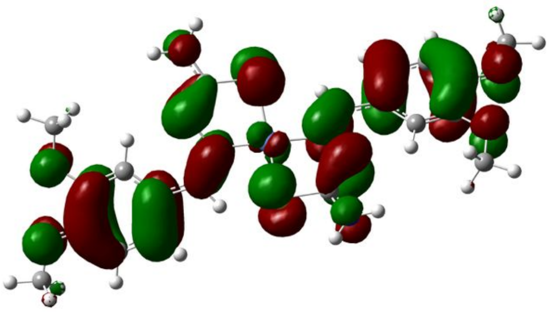

Ni1 (+1) HOMO

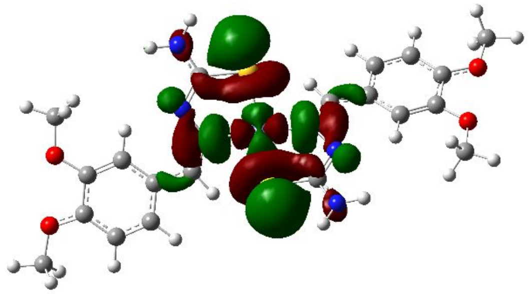

Ni1 (+1)LUMO

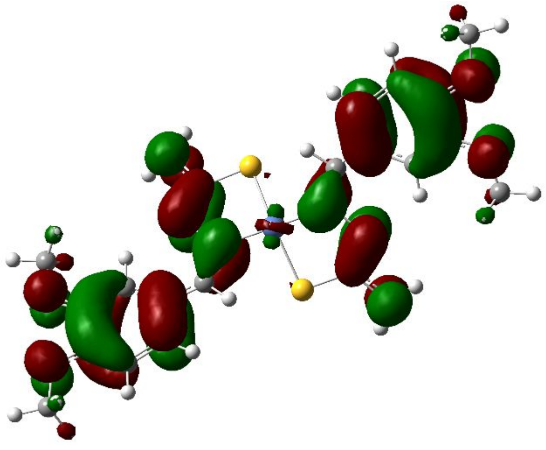

Ni1 (+2) HOMO

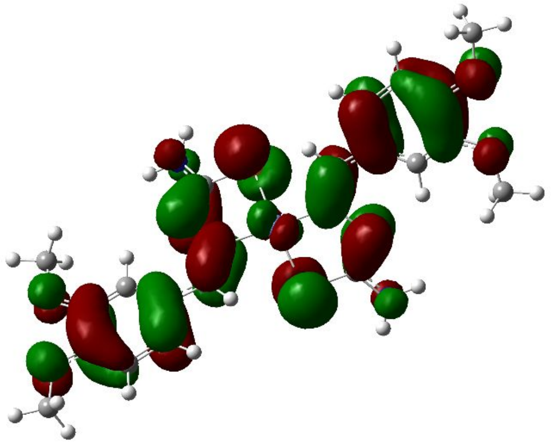

Ni1 (+2) LUMO

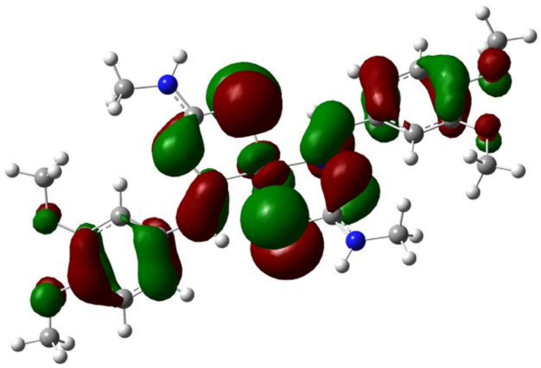

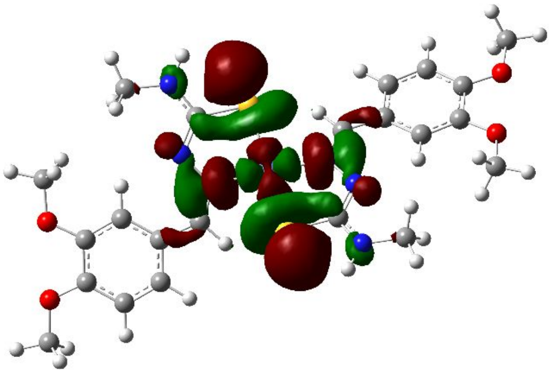

Ni2 HOMO

Ni2 LUMO

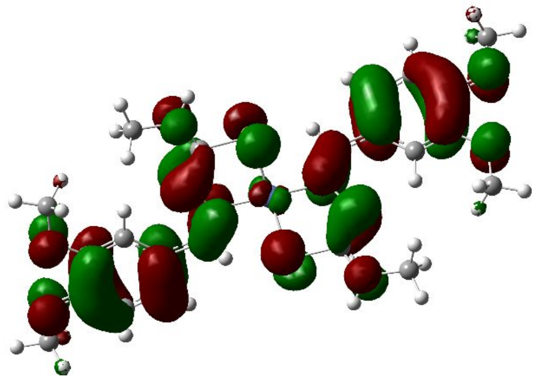

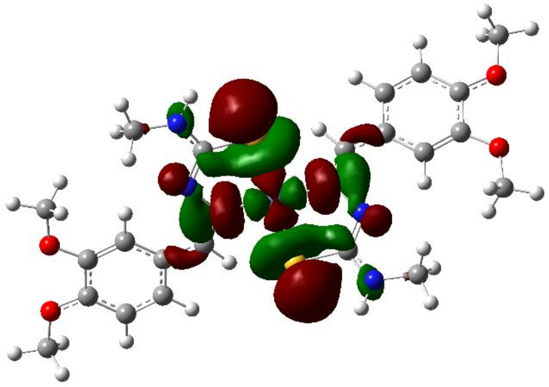

Ni2 (+1) HOMO

Ni2 (+1) LUMO

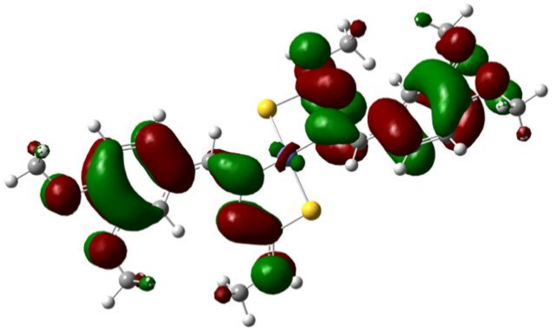

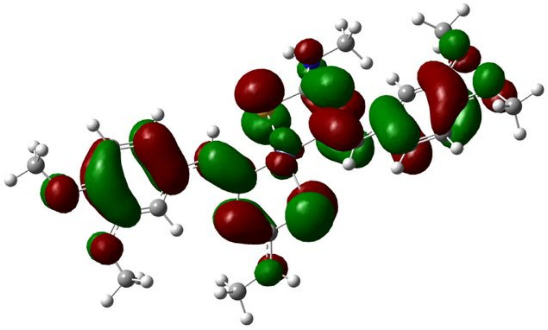

Ni2 (+2) HOMO

Ni2 (+2) LUMO

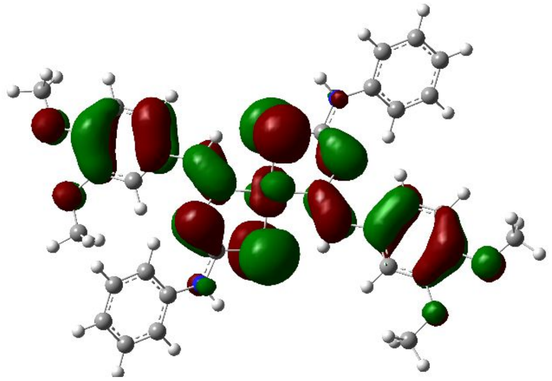

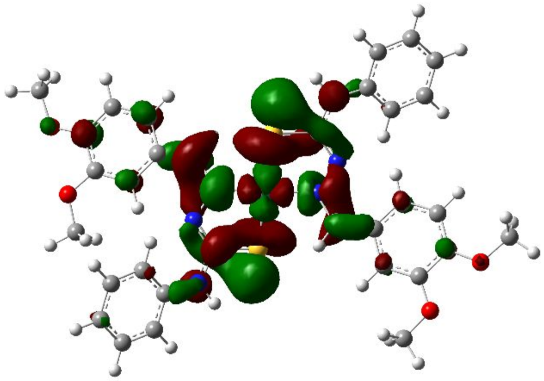

Ni3 HOMO

Ni3 LUMO

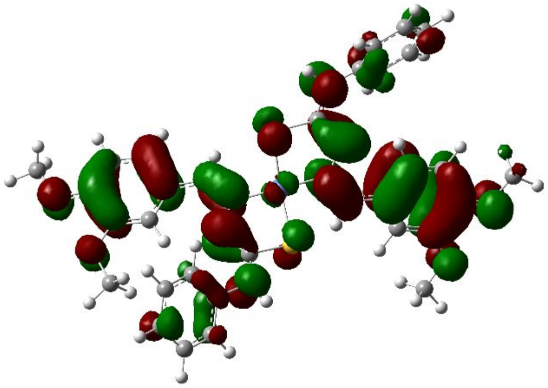

Ni3 (+1) HOMO

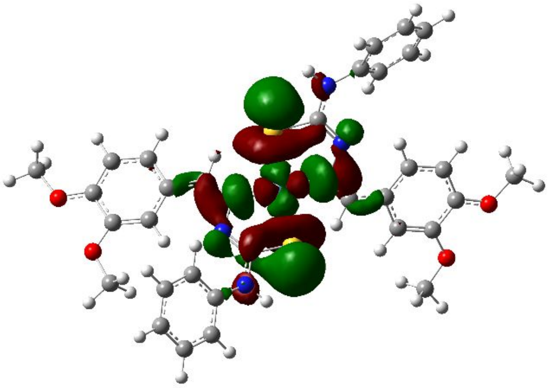

Ni3 (+1) LUMO

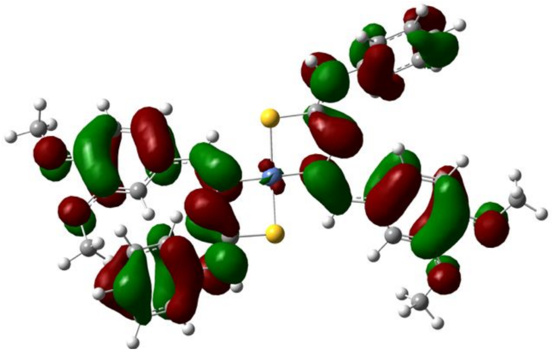

Ni3 (+2) HOMO

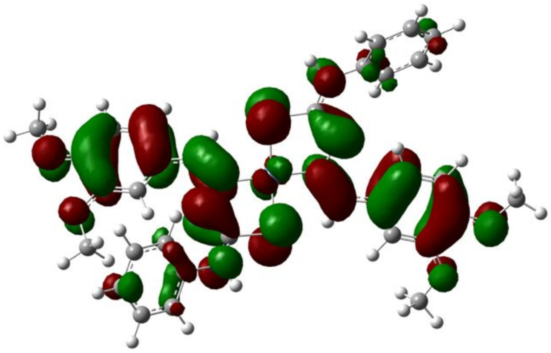

Ni3 (+2) LUMO

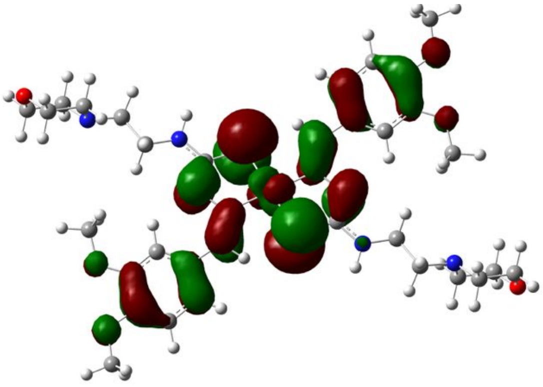

Ni4 HOMO

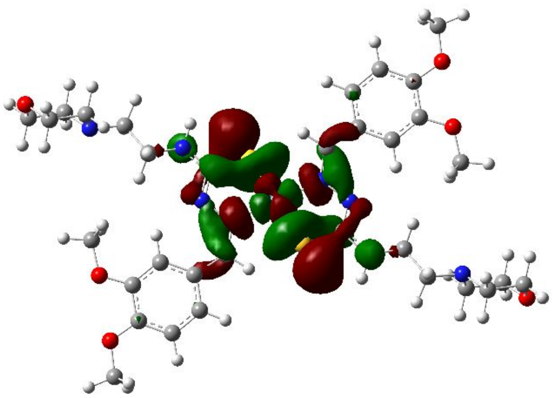

Ni4 LUMO

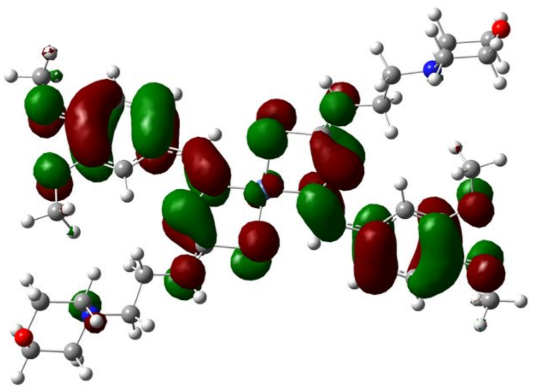

Ni4 (+1) HOMO

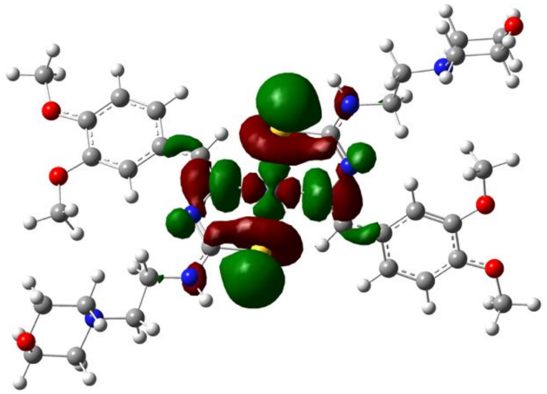

Ni4 (+1)LUMO

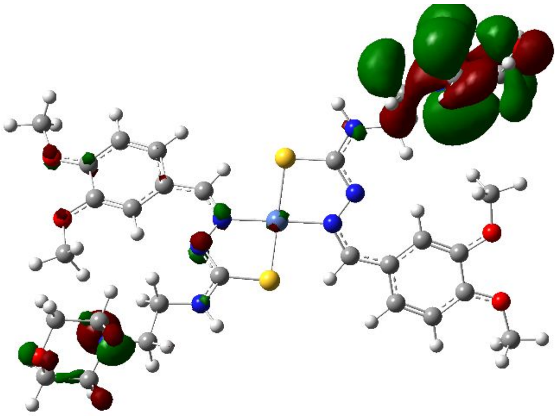

Ni4 (+2) HOMO

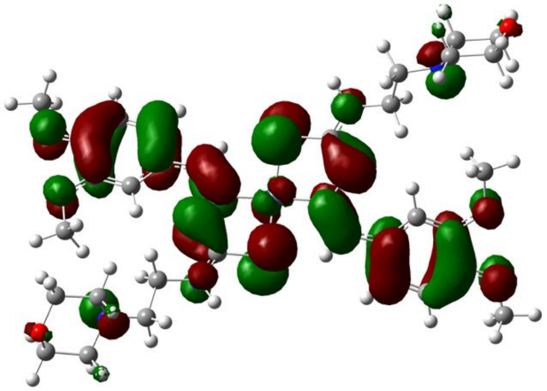

Ni4 (+2) LUMO

Figure S37: HOMO and LUMO plots (isovalue=0.02, density=0.004) for all the complex computed at B3LYP (6-31+g**) level of theory.

3. CYTOTOXICITY ASSAYS: IC_{50} fit curves

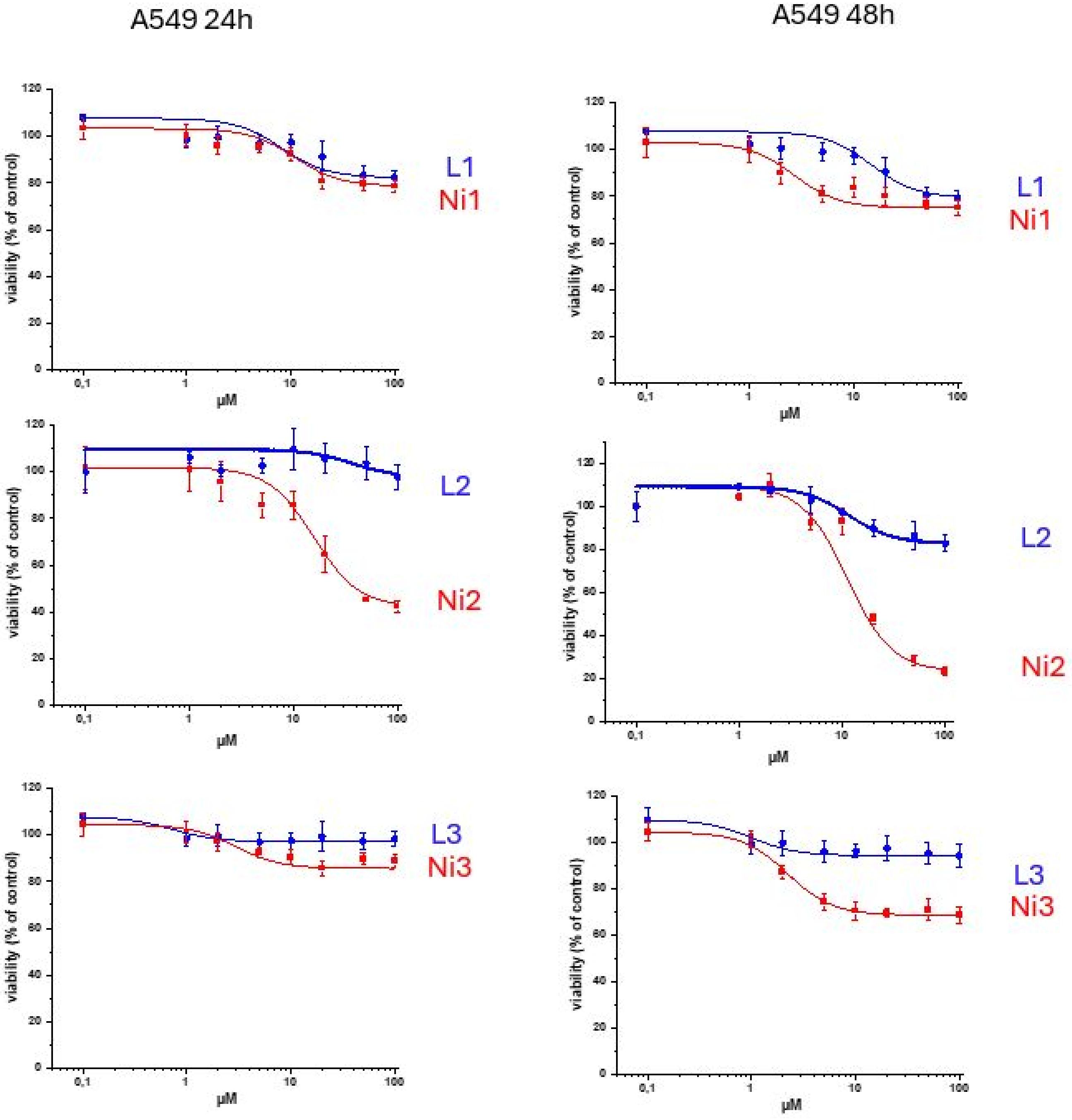
A549 24h
A549 48h
L1
Ni1
L2
Ni2
L3
Ni3
viability (% of control)
µM
0,1
1
10
100
0
20
40
60
80
100
120

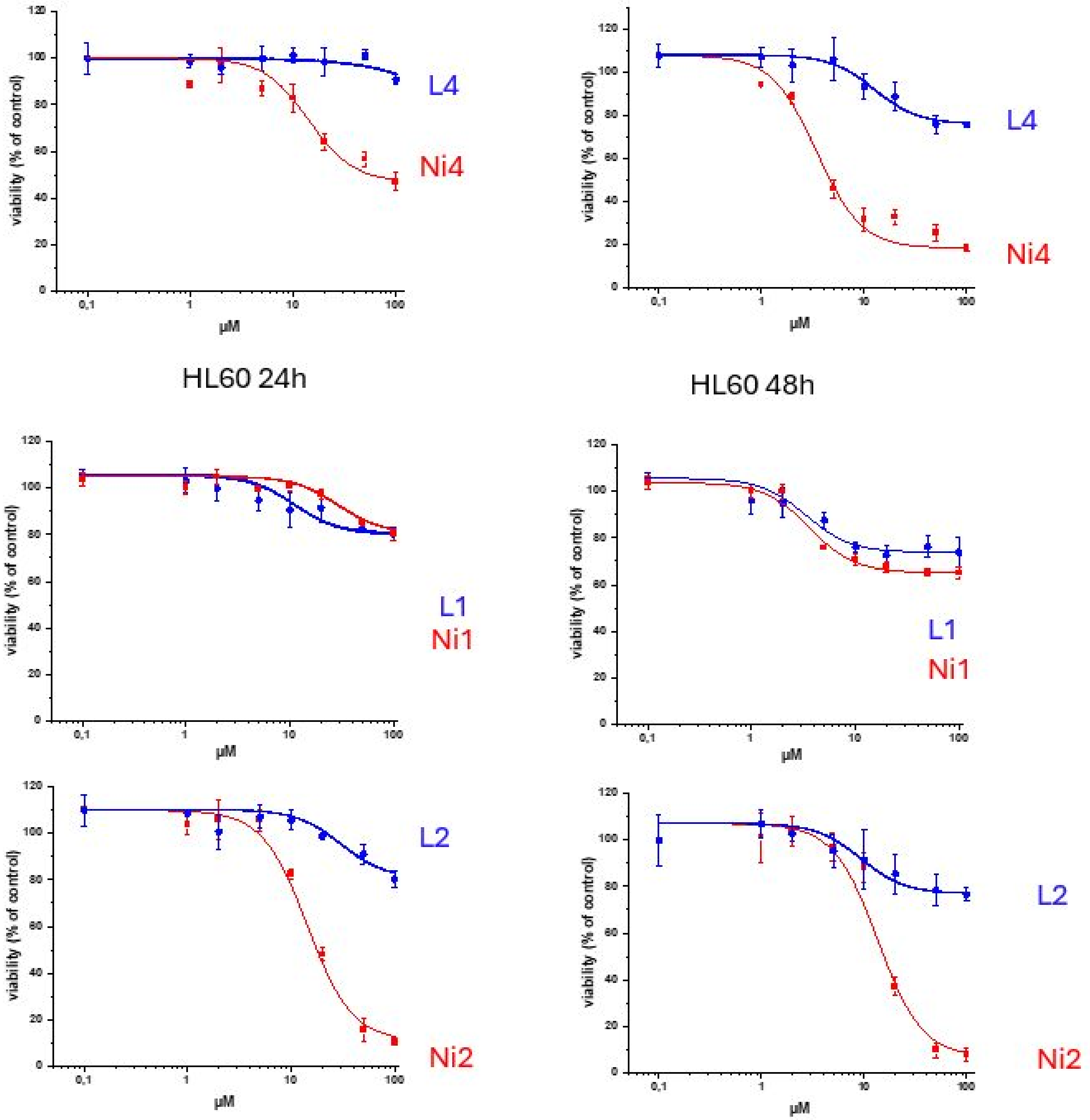
HL60 24h
HL60 48h
viability (% of control)
μM
0,1
1
10
100
L4
Ni4
L1
Ni1
L2
Ni2

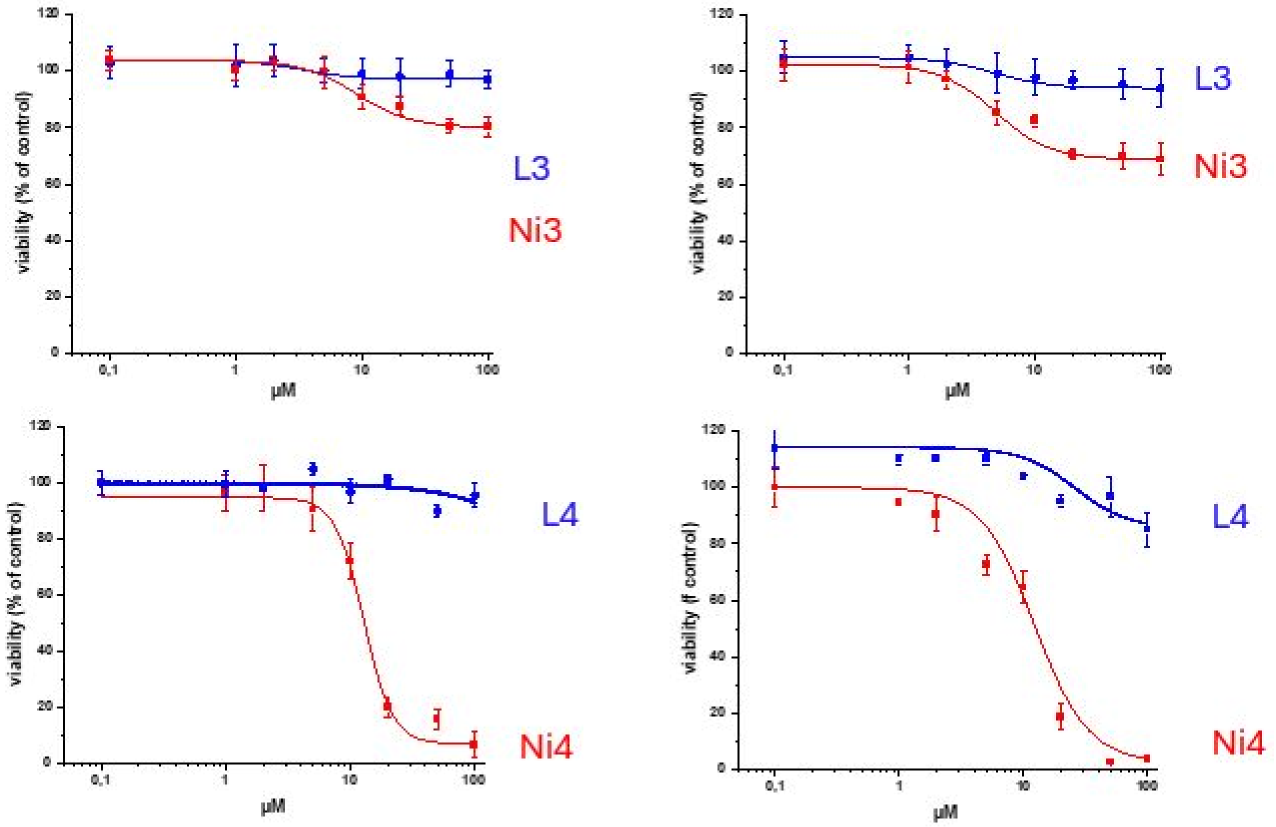

HT29 24h HT29 48h

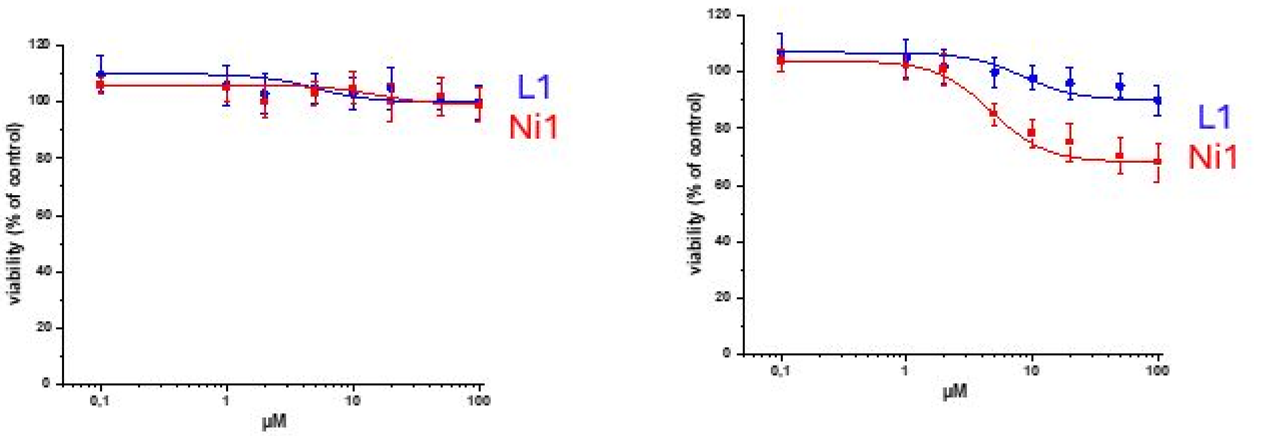

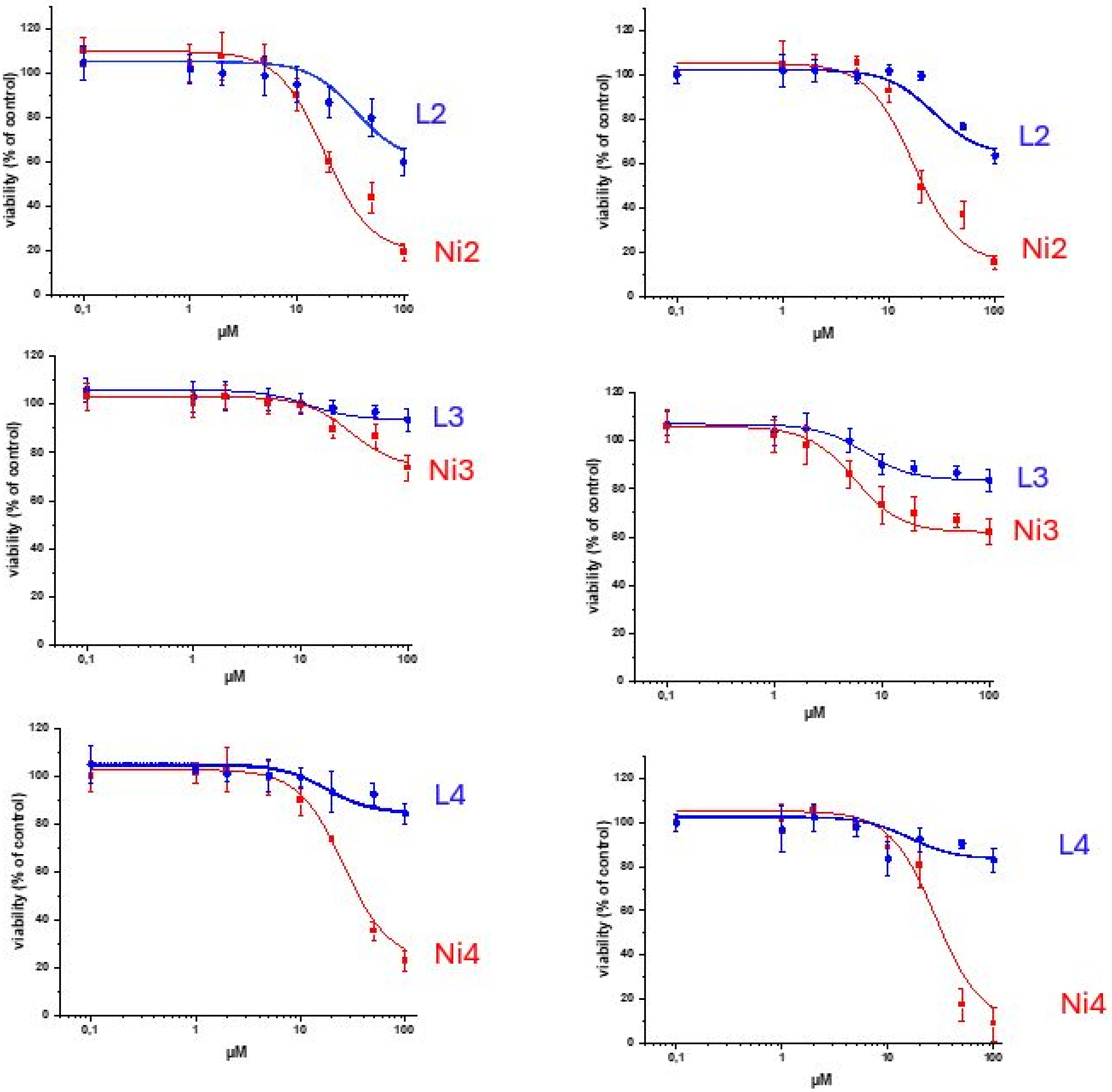
viability (% of control)
µM
0,1
1
10
100
0
20
40
60
80
100
120
L2
Ni2
L3
Ni3
L4
Ni4

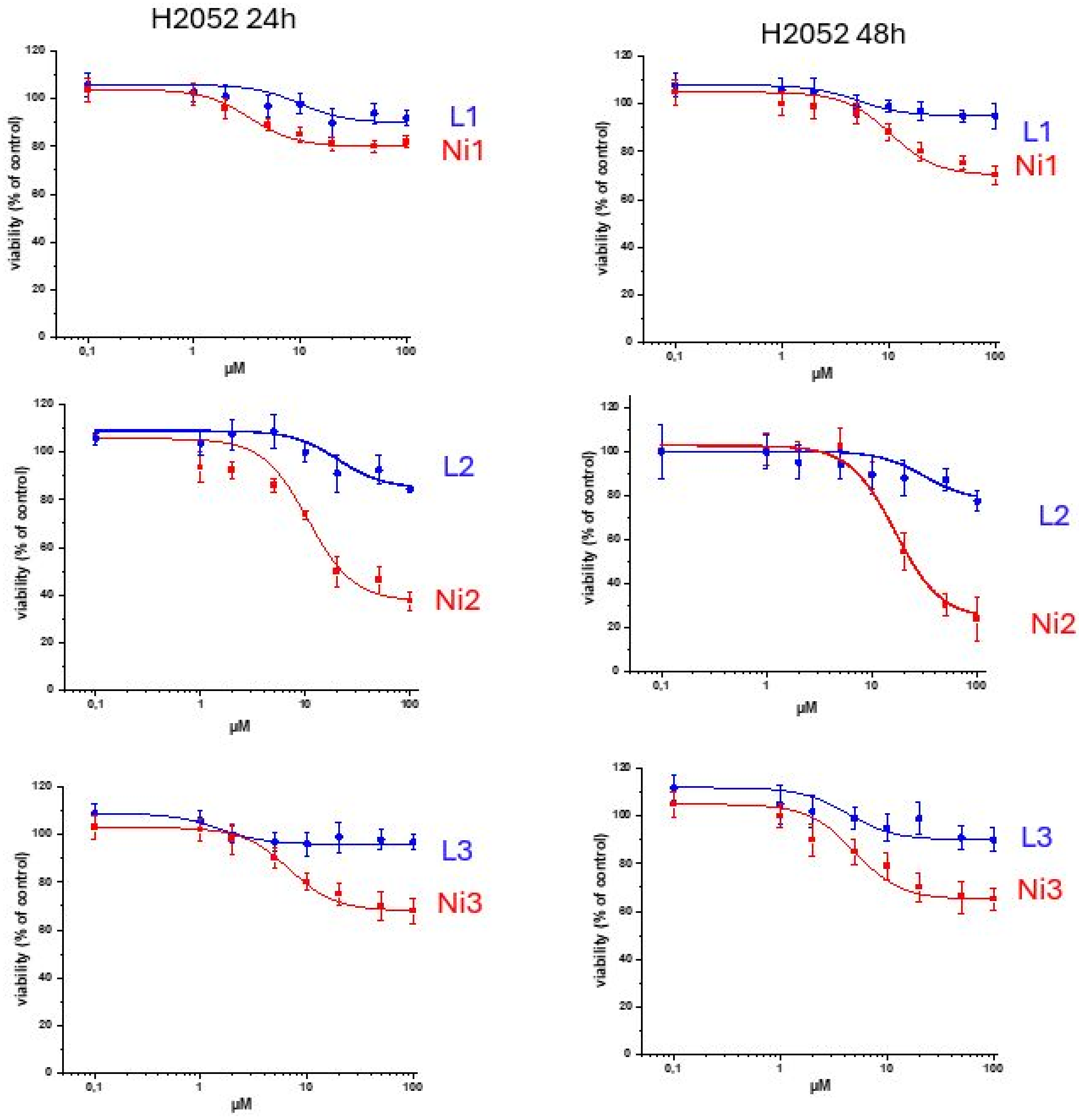
H2052 24h
H2052 48h
viability (% of control)
μM
0,1
1
10
100
0
20
40
60
80
100
120
L1
Ni1
L2
Ni2
L3
Ni3

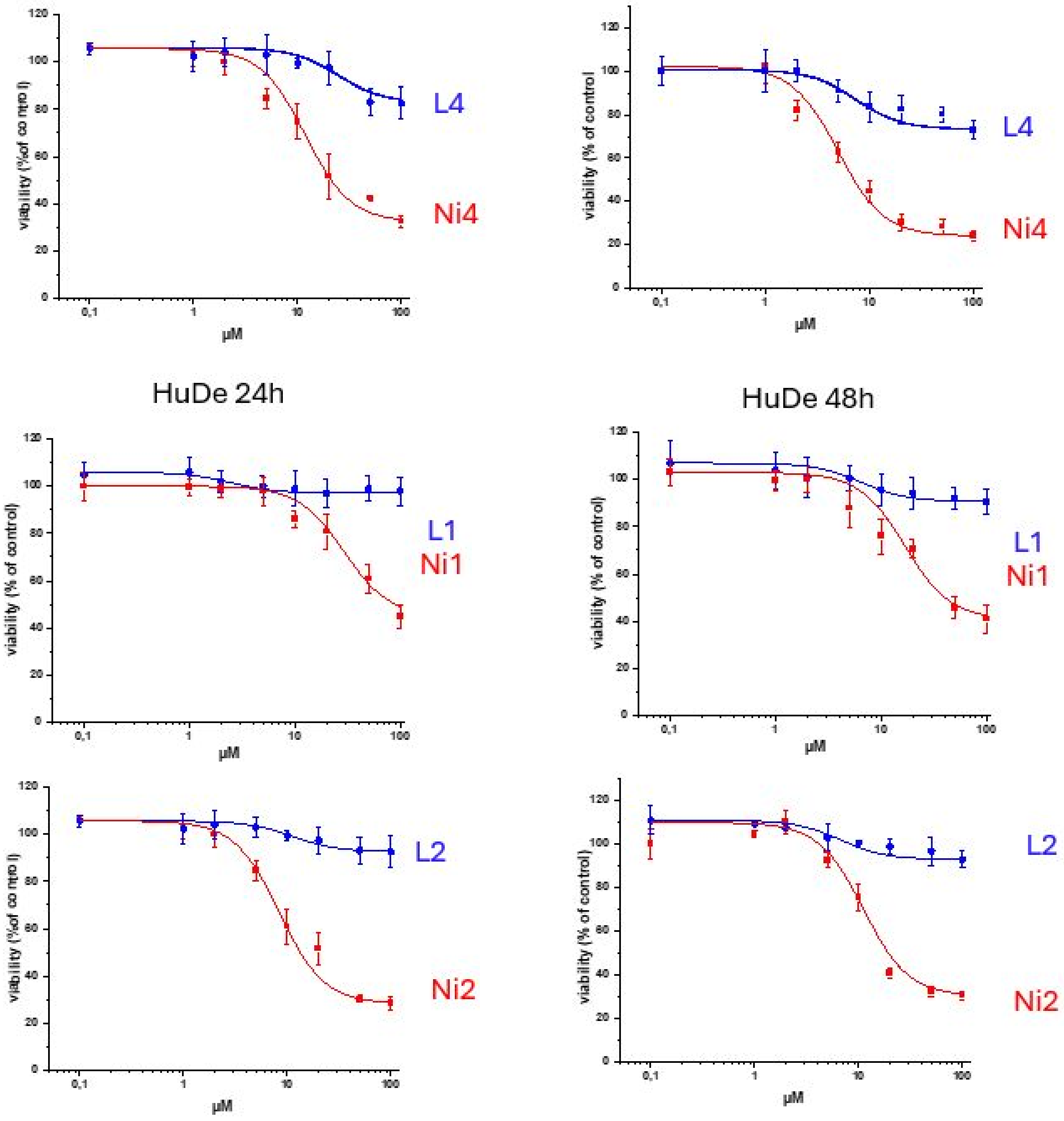
HuDe 24h
HuDe 48h
L4
Ni4
L1
Ni1
L2
Ni2
viability (% of control)
μM

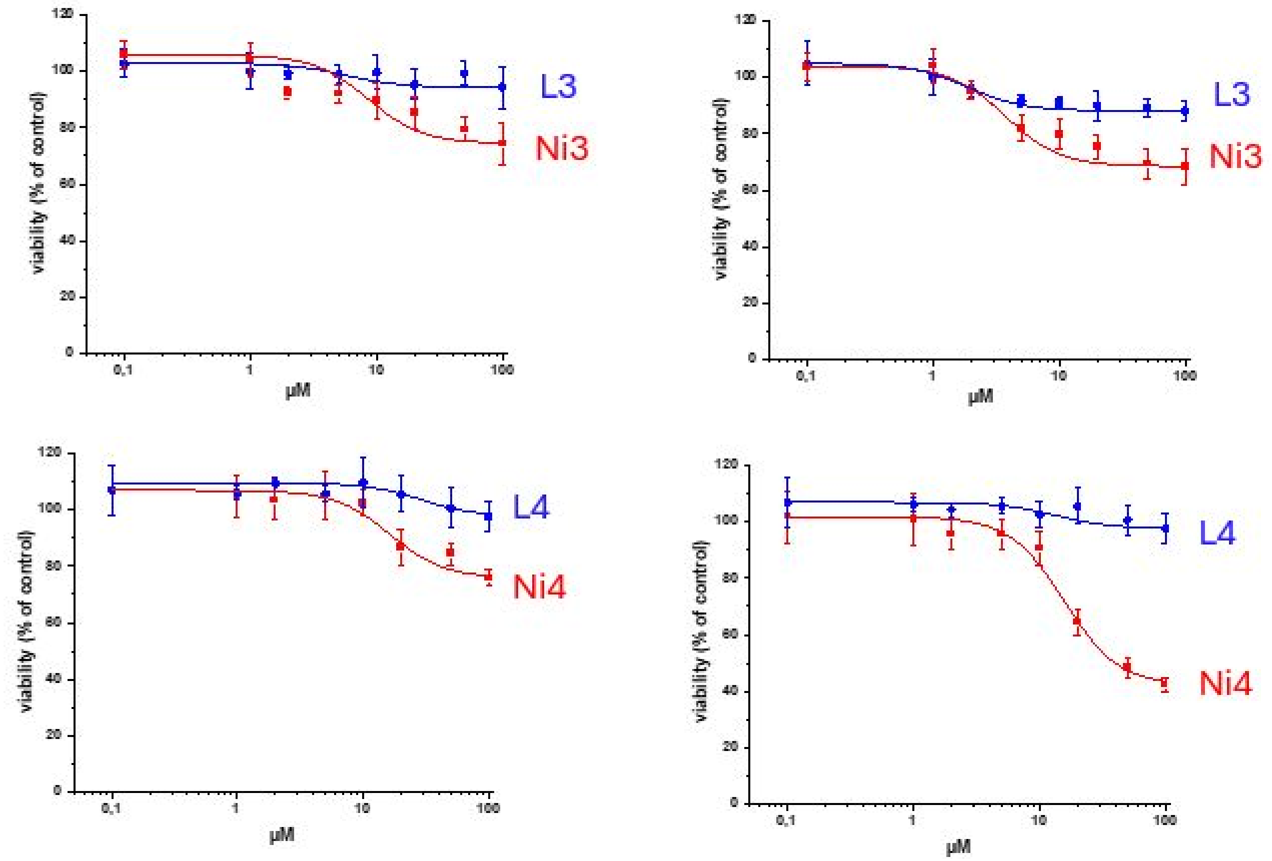

Figure S38: IC_{50} fit curves with cancer cell lines (A549, HL60, HT29, H2052) and healthy human dermal fibroblasts (HuDe) as a comparison (24 and 48 h).

4. ALBUMIN-MEDIATED DELIVERY

4.1 *Galleria mellonella* larval model

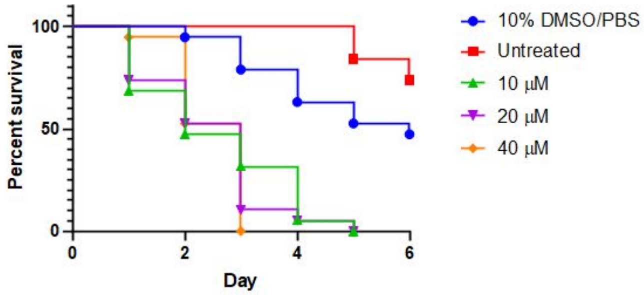

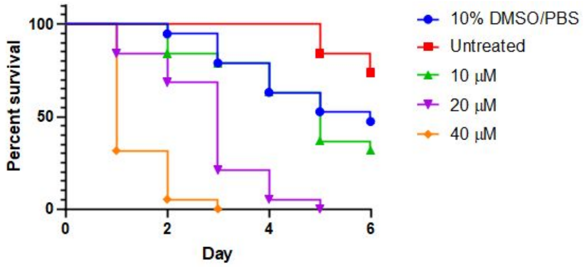

L1 **Ni1**

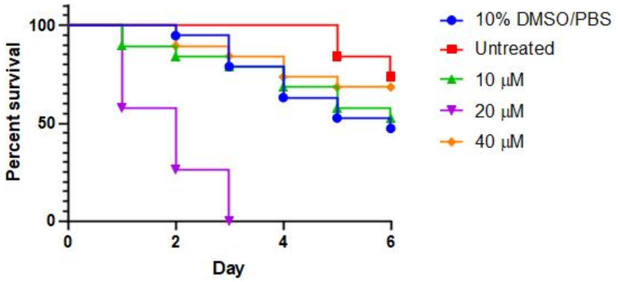

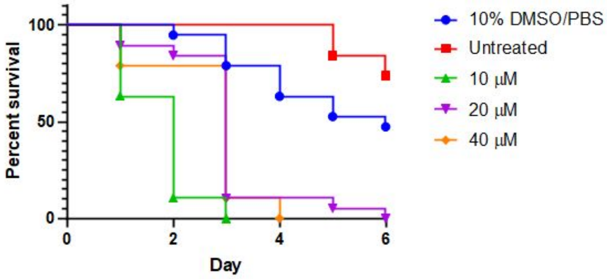

L2 **Ni2**

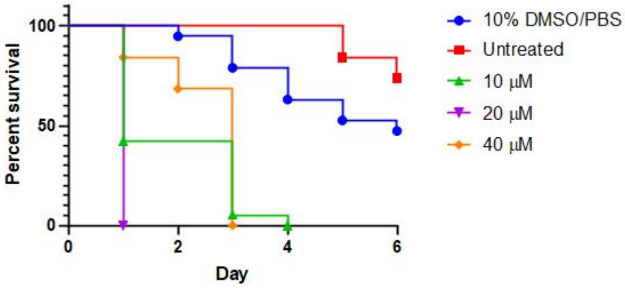

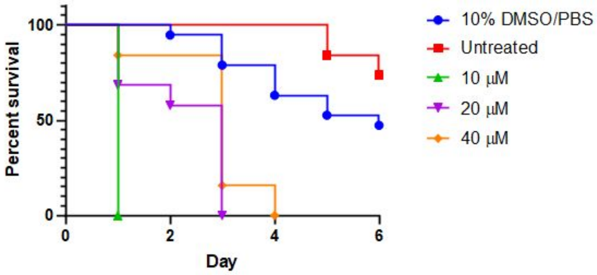

L3 **Ni3**

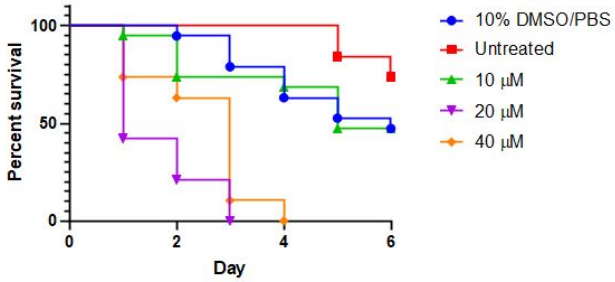

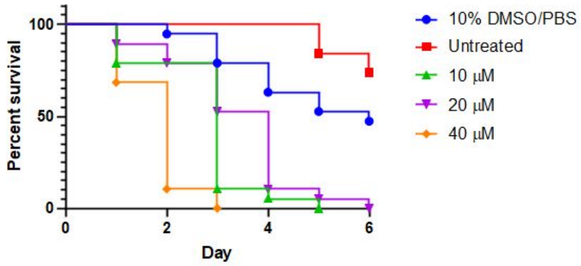

L4 **Ni4**

Figure S39: Survival of *Galleria mellonella* larvae in the 400-500 mg range divided into groups of 19 each and placed in large Petri plates recorded over a 6-Day period of incubation at 37.4 ˚C post-injection of 10 µL of concentrated solutions (1.1 mM, 0.57 mM, 0.28 mM*)* of the compounds in distilled water (10% DMSO)*,* diluted in larva to the reported concentrations.

This experiment reported a high mortality rate for the whole set of compounds, chelators included. It is important to acknowledge that even the control group, which was injected with the clean solvent, exhibited an unexpectedly high mortality rate, but nonetheless comparable to compounds. Furthermore, compounds that were previously reported as non-cytotoxic against cancer cell lines (such as **L2** or **L4**) also resulted in high mortality towards these larvae. Considering the importance albumin seems to have in delivering these compounds, we hypothesize that the absence of albumin in *G. mellonella* might have caused this high mortality rate[4,5].

4.2 Albumin affinity: circular dichroism

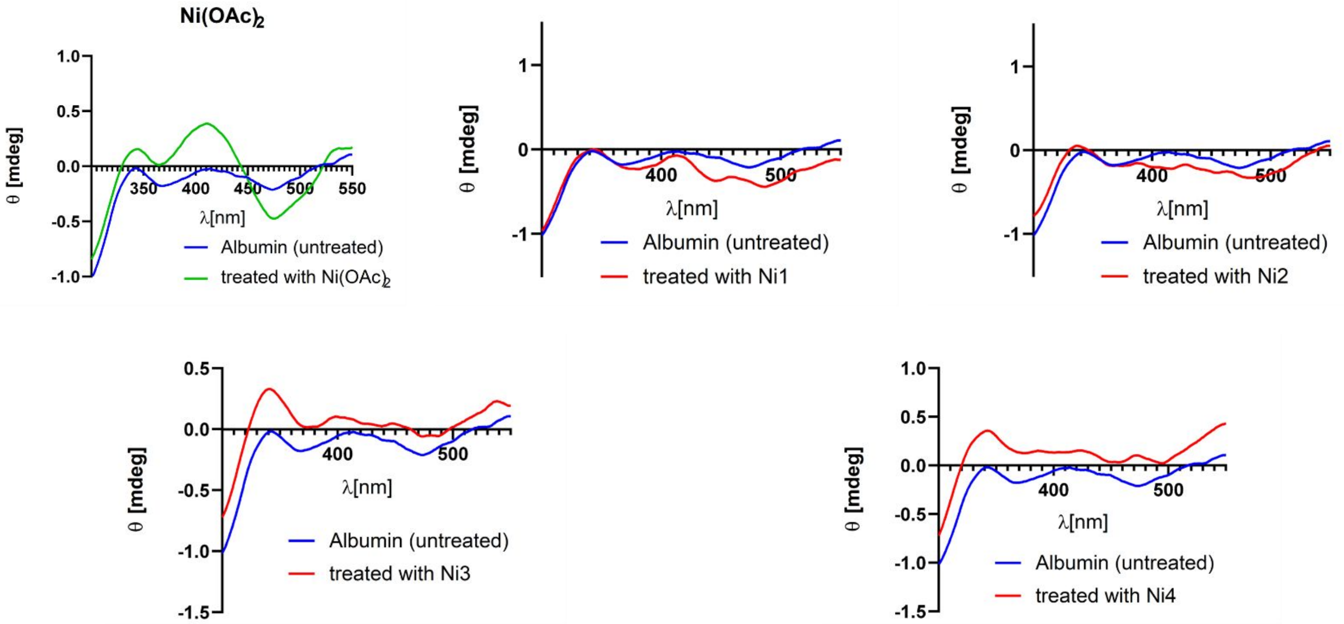

Figure S40: Comparison between the Cotton effect (417 nm positive and 473 nm negative) of the complex BSA-Ni(II) to the CD spectra from BSA and complexes **Ni1-4** mixed in pH 7.4 double distilled water (with DMSO to help dissolution below 5%).

Compounds and BSA were respectively diluted to 7 μM and 12 μM.

4.3 Albumin affinity: fluorescence titrations

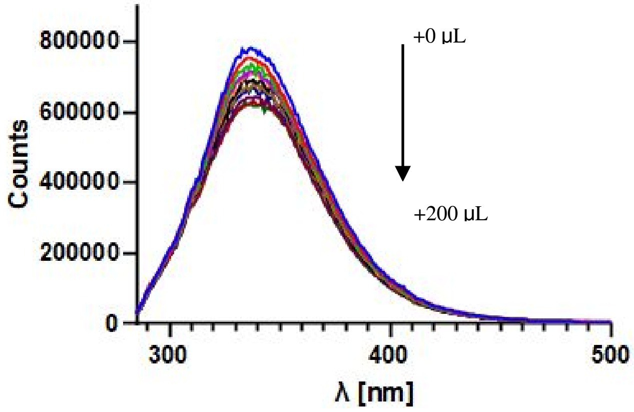

L1

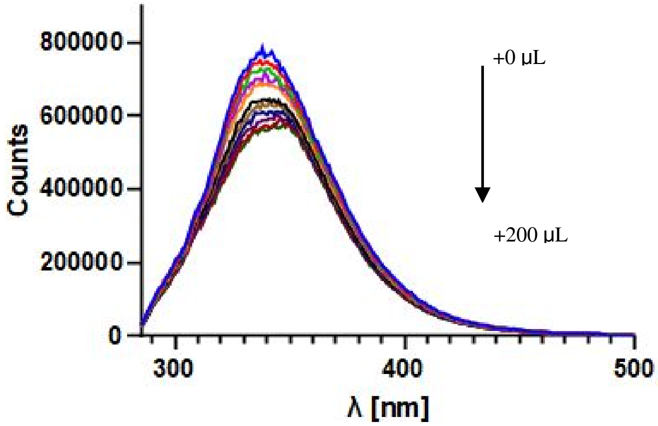

Ni1

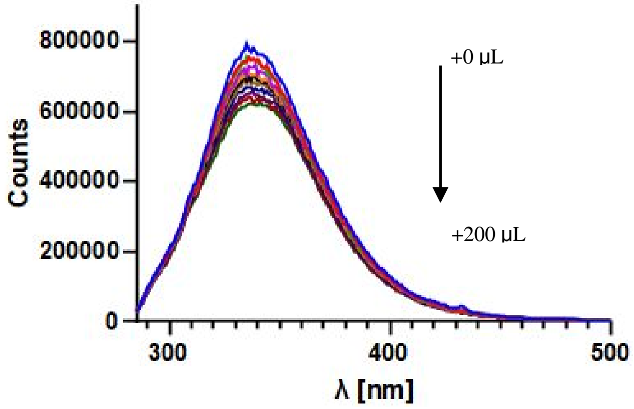

L2

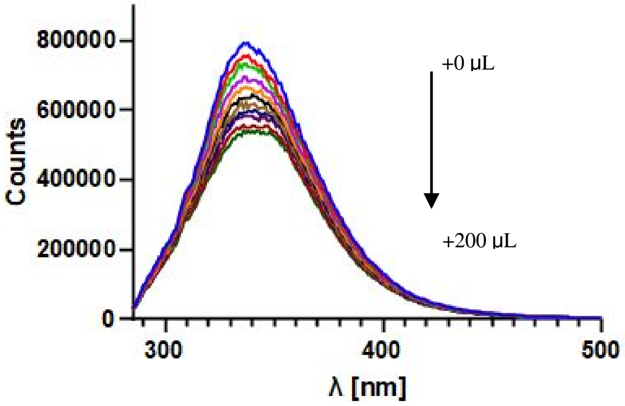

Ni2

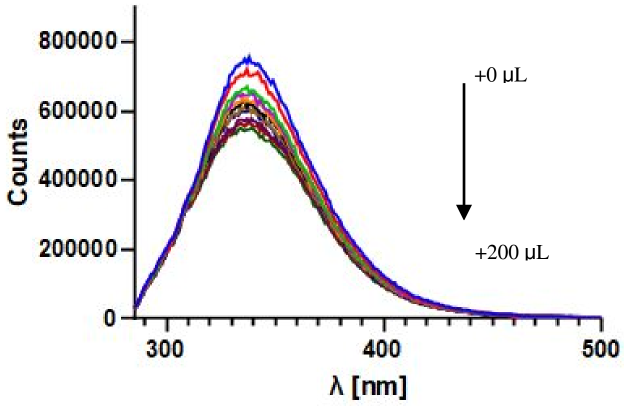

L3

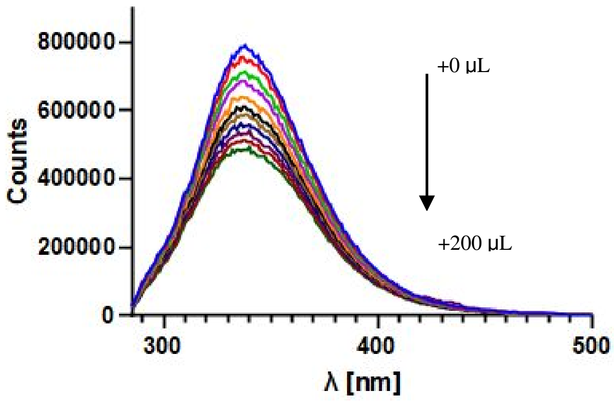

Ni3

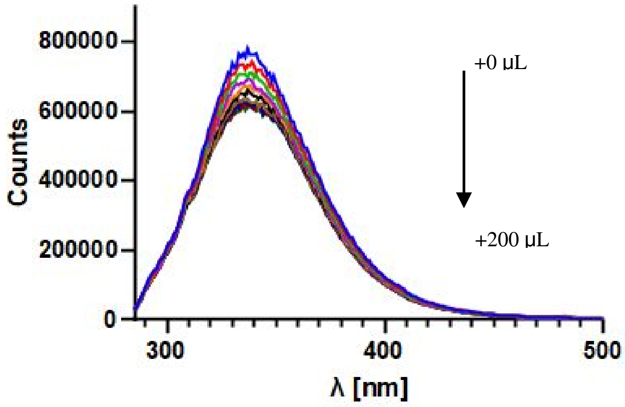

L4

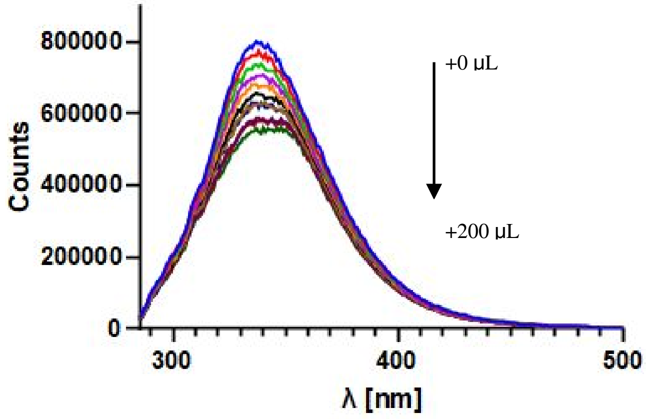

Ni4

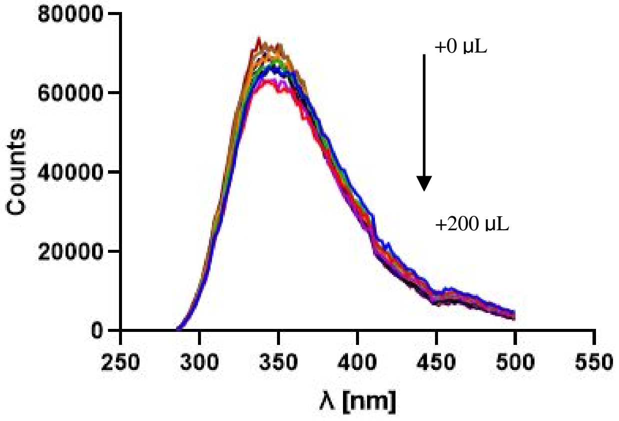

Ni(OAc)$_2$

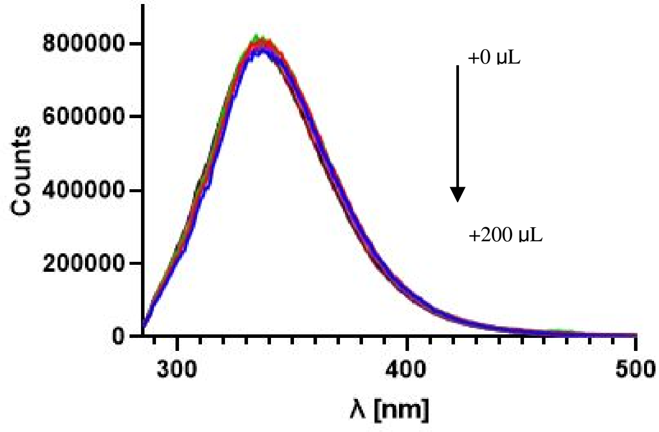

DMSO

Figure S41: BSA fluorescence titrations with target complexes, chelators, metallic salt and solvent.

5. DNA INTERACTION PATHWAY: ethidium bromide displacement titrations

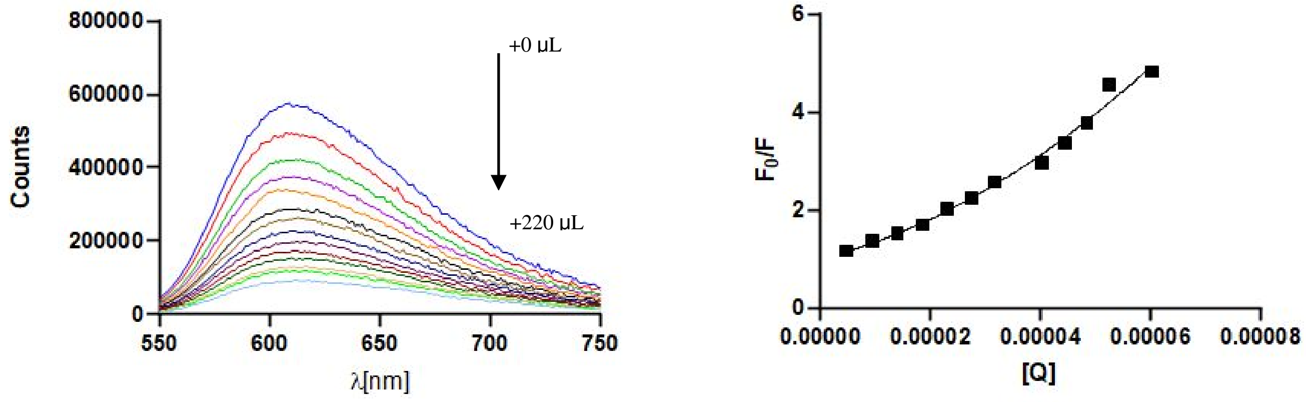

Ni1

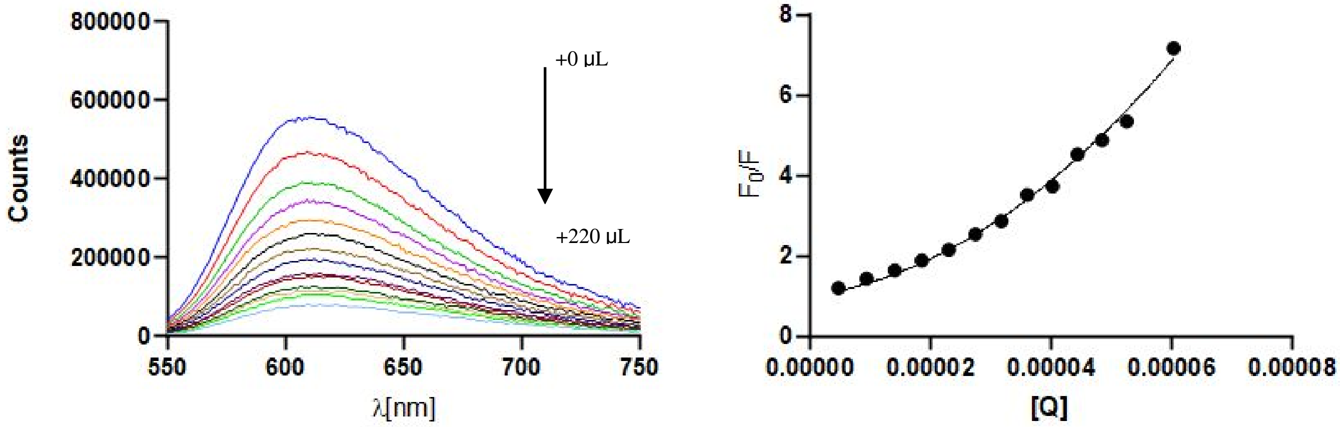

Ni2

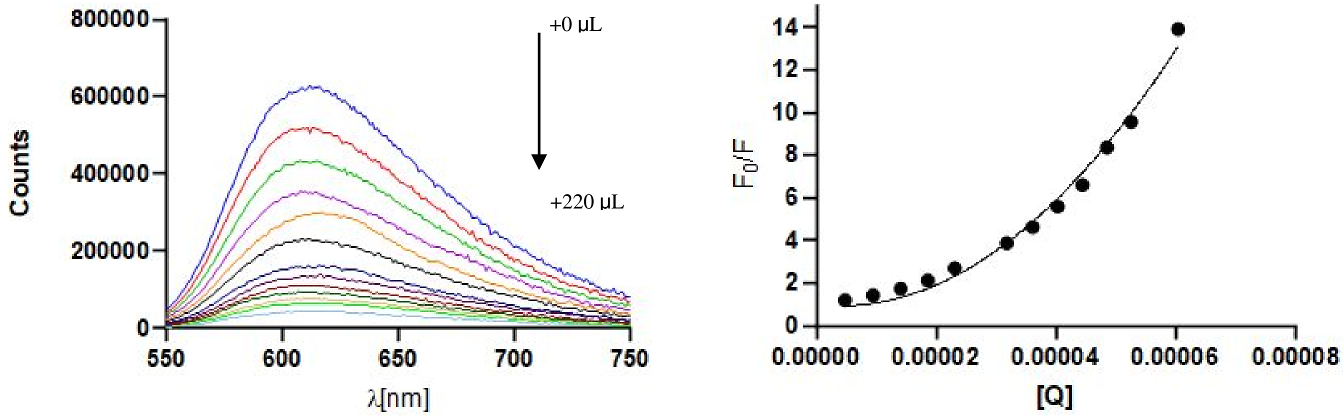

Ni3

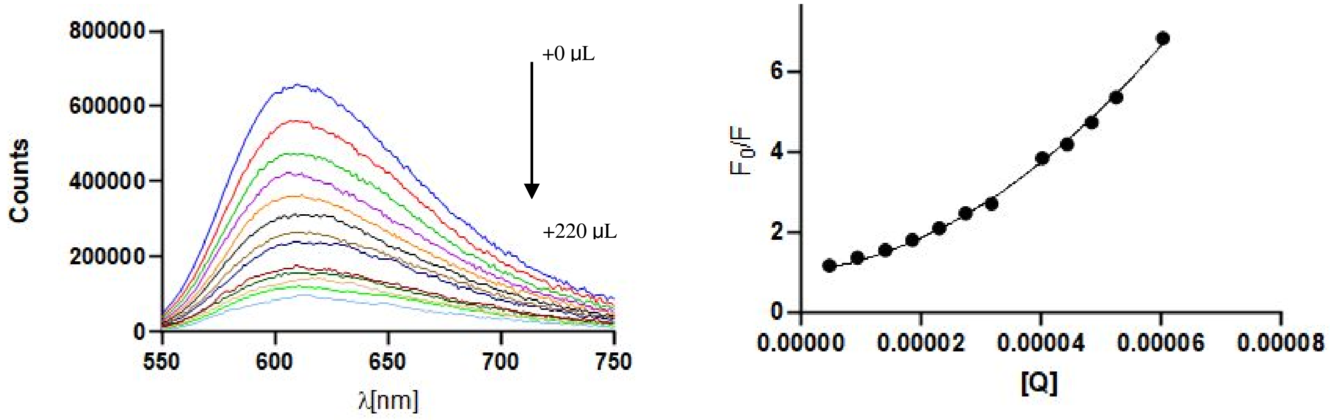
800000
600000
400000
200000
0
Counts
550
600
650
700
750
λ[nm]
+0 µL
+220 µL
6
4
2
0
F0/F
0.00000
0.00002
0.00004
0.00006
0.00008
[Q]

Ni4

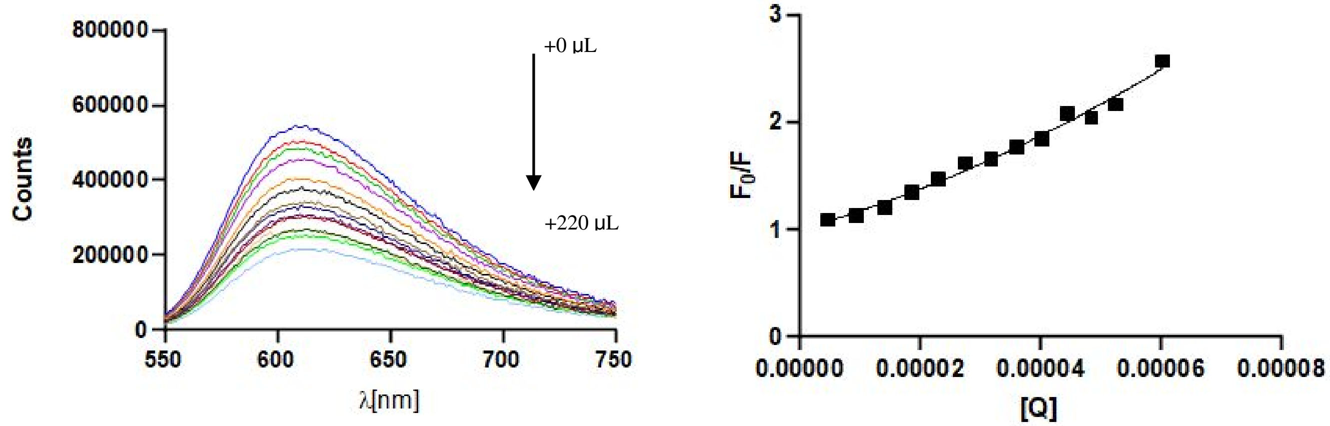
800000
600000
400000
200000
0
Counts
550
600
650
700
750
λ[nm]
+0 µL
+220 µL
3
2
1
0
F0/F
0.00000
0.00002
0.00004
0.00006
0.00008
[Q]

L1

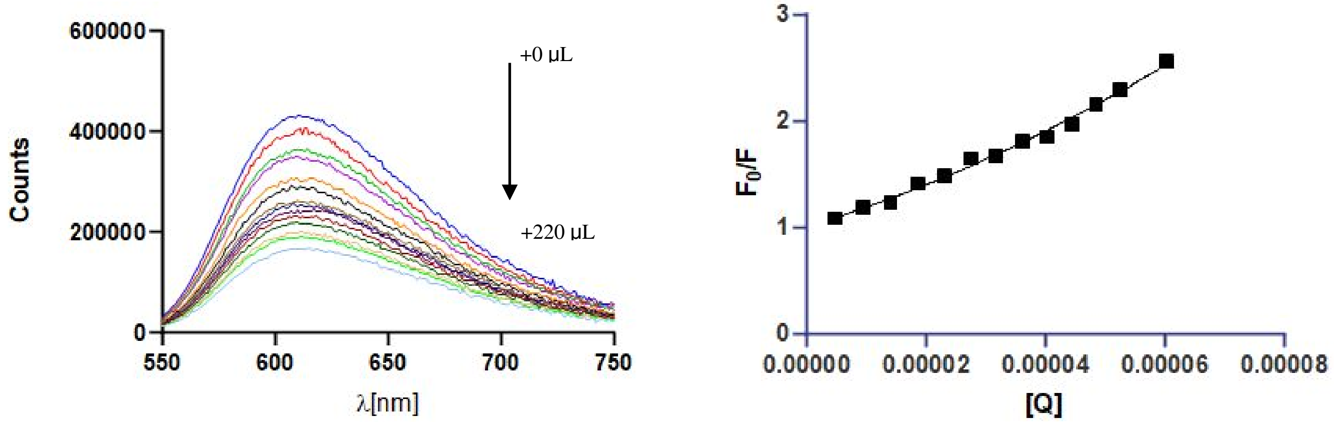
600000
400000
200000
0
Counts
550
600
650
700
750
λ[nm]
+0 µL
+220 µL
3
2
1
0
F0/F
0.00000
0.00002
0.00004
0.00006
0.00008
[Q]

L2

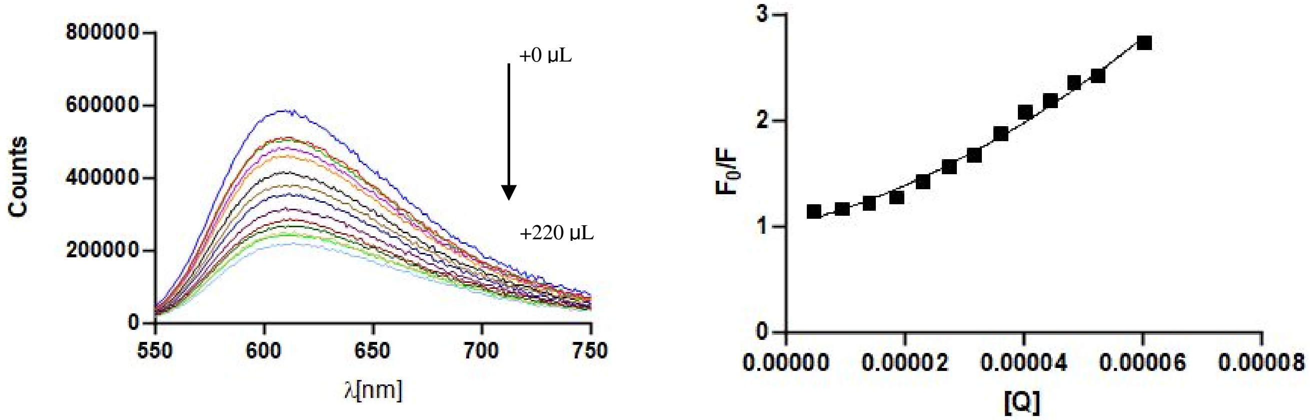
800000
600000
400000
200000
0
Counts
550
600
650
700
750
λ[nm]
+0 μL
+220 μL
3
2
1
0
F0/F
0.00000
0.00002
0.00004
0.00006
0.00008
[Q]

L3

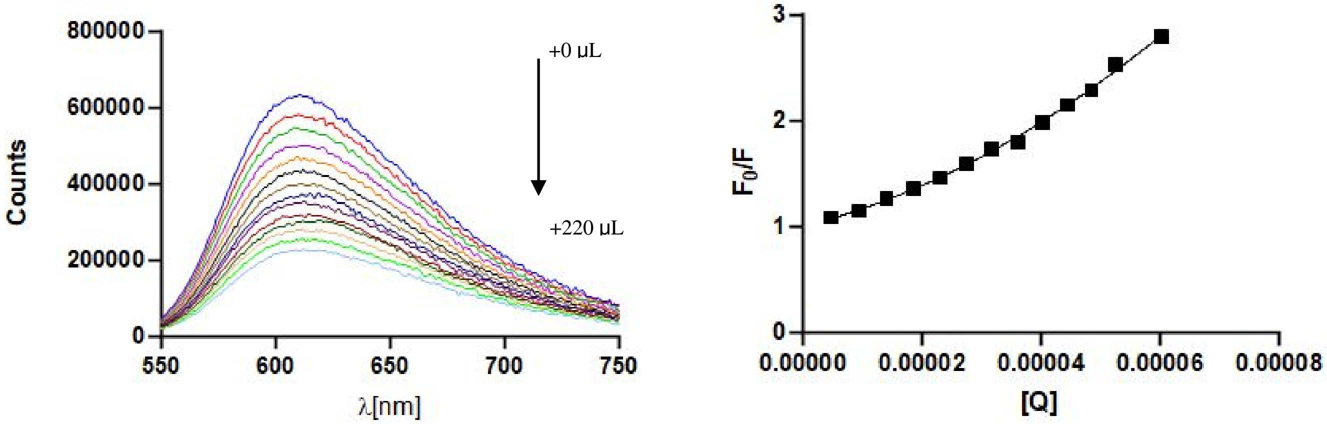
800000
600000
400000
200000
0
Counts
550
600
650
700
750
λ[nm]
+0 μL
+220 μL
3
2
1
0
F0/F
0.00000
0.00002
0.00004
0.00006
0.00008
[Q]

L4

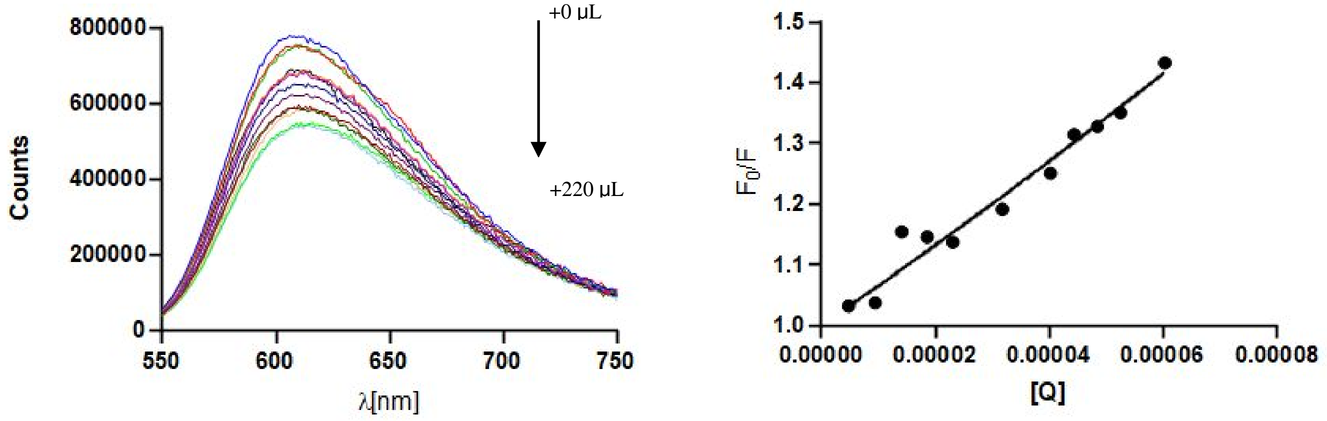
800000
600000
400000
200000
0
Counts
550
600
650
700
750
λ[nm]
+0 μL
+220 μL
1.5
1.4
1.3
1.2
1.1
1.0
F0/F
0.00000
0.00002
0.00004
0.00006
0.00008
[Q]

$Ni(OAc)_2$

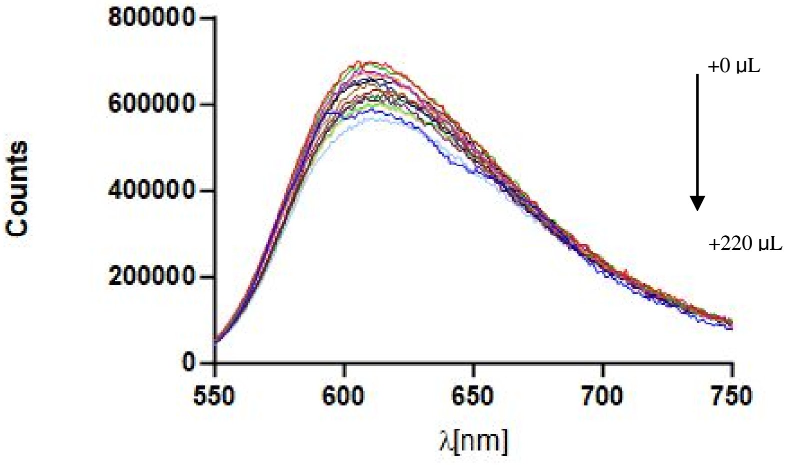

DMSO

Figure S42: Ethidium bromide displacement assay spectra (left) and plotted data to calculate K_{app} (right).

6. ROS-MEDIATED PATHWAY: Clark-type oximetry

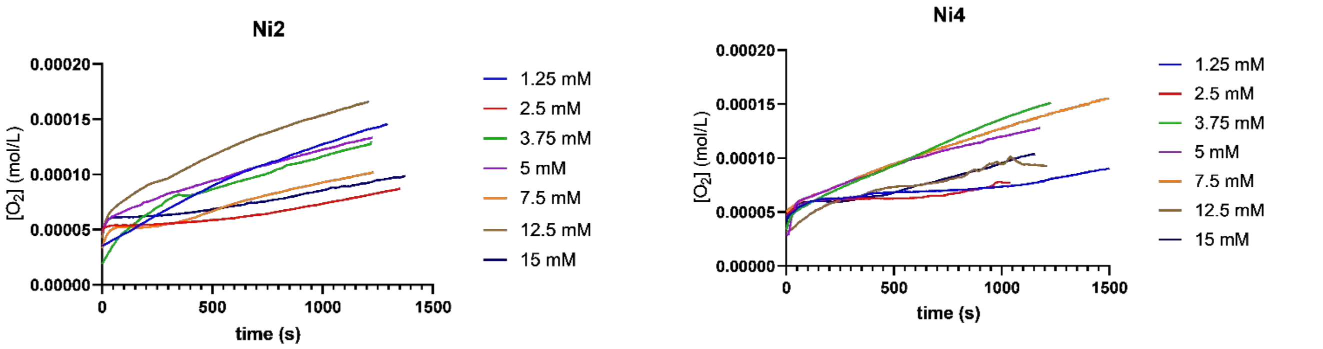

Figure S43: Dioxygen evolution monitored using a Clark-type electrode of target Ni(II) complexes at 50 µM in PBS buffer (pH 7.4, [PBS]=10 mM, [NaCl]=137 mM, [KCl]=2.7 mM) with 5% DMSO in presence of increasing concentration of [H_2O_2] (from 1.25 mM to 15 mM).

7. REFERENCES

(1) Rogolino, D.; Gatti, A.; Carcelli, M.; Pelosi, G.; Bisceglie, F.; Restivo, F. M.; Degola, F.; Buschini, A.; Montalbano, S.; Feretti, D.; Zani, C. Thiosemicarbazone Scaffold for the Design of Antifungal and Antiaflatoxigenic Agents: Evaluation of Ligands and Related

Copper Complexes. *Sci. Rep.* **2017**, *7* (1), 1–12. https://doi.org/10.1038/s41598-017-11716-w.

(2) Jaafar, A.; Fix-Tailler, A.; Mansour, N.; Allain, M.; Shebaby, W. N.; Faour, W. H.; Tokajian, S.; El-Ghayoury, A.; Naoufal, D.; Bouchara, J. P.; Larcher, G.; Ibrahim, G. Synthesis, Characterization, Antifungal and Antibacterial Activities Evaluation of Copper (II), Zinc (II) and Cadmium (II) Chloride and Bromide Complexes with New (E)-1-(3,4-Dimethoxybenzylidene)-4-Methylthiosemicarbazone Ligand. *Appl. Organomet. Chem.* **2020**, *34* (12), 1–13. https://doi.org/10.1002/aoc.5988.

(3) Pitucha, M.; Korga-Plewko, A.; Czylkowska, A.; Rogalewicz, B.; Drozd, M.; Iwan, M.; Kubik, J.; Humeniuk, E.; Adamczuk, G.; Karczmarzyk, Z.; Fornal, E.; Wysocki, W.; Bartnik, P. Influence of Complexation of Thiosemicarbazone Derivatives with Cu (II) Ions on Their Antitumor Activity against Melanoma Cells. *Int. J. Mol. Sci.* **2021**, *22* (6), 1–25. https://doi.org/10.3390/ijms22063104.

(4) Belinskaia, D. A.; Voronina, P. A.; Goncharov, N. V. Integrative Role of Albumin: Evolutionary, Biochemical and Pathophysiological Aspects. *J. Evol. Biochem. Physiol.* **2021**, *57* (6), 1419–1448. https://doi.org/10.1134/s002209302106020x.

(5) Li, S.; Cao, Y.; Geng, F. Genome-Wide Identification and Comparative Analysis of Albumin Family in Vertebrates. *Evol. Bioinforma.* **2017**, *13*. https://doi.org/10.1177/1176934317716089.
